# Randomized, placebo-controlled study on efficacy, safety and tolerability of drug-induced defibrinogenation for sudden sensorineural hearing loss: the lessons learned

**DOI:** 10.1007/s00405-023-07896-z

**Published:** 2023-03-07

**Authors:** Bernhard G. Weiss, Jennifer L. Spiegel, Sven Becker, Sebastian Strieth, Bernhard Olzowy, Mattis Bertlich, Tomáš Fořt, Jan Mejzlik, Thomas Lenarz, Friedrich Ihler, Martin Canis

**Affiliations:** 1 Department of Otorhinolaryngology, University Hospital, LMU Munich, Marchioninistr. 15, 81377 Munich, Germany; 2 Department of Otorhinolaryngology, Head and Neck Surgery, University of Tübingen, Elfriede-Aulhorn-Straße 5, 72076 Tübingen, Germany; 3 Department of Otorhinolaryngology, Head and Neck Surgery, University Hospital Bonn, Venusberg-Campus 1, 53127 Bonn, Germany; 4 HNO-Zentrum Landsberg am Lech, Ahornallee 2a, 86899 Landsberg am Lech, Germany; 5 FORTMEDICA s.r.o., ORL Modřany, Poliklinika Modřany, Soukalova 3355, 143 00 Prague 4, Czech Republic; 6 Department of Otorhinolaryngology and Head and Neck Surgery, Faculty of Medicine in Hradec Kralove, University Hospital Hradec Kralove, Charles University, Sokolska 581, 500 05 Hradec Kralove, Czech Republic; 7 Department of Otorhinolaryngology, Hannover Medical School, Carl-Neuberg-Str. 1, 30625 Hannover, Germany; 8 German Center for Vertigo and Balance Disorders (DSGZ), University Hospital, LMU Munich, Marchioninistr. 15, 81377 Munich, Germany; 9 Department of Dermatology and Allergy, University Hospital, LMU Munich, Marchioninistr. 15, Thalkirchner Str. 48, 80337 Munich, Germany; 10 Department of Otorhinolaryngology, Head and Neck Surgery, University Medical Center Greifswald, Ferdinand-Sauerbruch-Strasse, 17475 Greifswald, Germany

**Keywords:** Sudden sensorineural hearing loss, Cochlear microcirculation, Defibrinogenation, Ancrod, Fibrinogen

## Abstract

**Purpose:**

Disturbance of cochlear microcirculation is discussed as final common pathway of various inner ear diseases. Hyperfibrinogenemia causing increased plasma viscosity is a possible factor for a critical reduction of cochlear blood flow that might lead to sudden sensorineural hearing loss (SSHL). The aim was to determine the efficacy and safety of drug-induced defibrinogenation by ancrod for SSHL.

**Methods:**

Double-blind, randomized, placebo-controlled, multicenter, parallel group, phase II (proof-of-concept) study (planned enrollment: 99 patients). Patients received an infusion of ancrod or placebo (day 1) followed by subcutaneous administrations (day 2, 4, 6). Primary outcome was the change in pure tone audiogram air conduction average until day 8.

**Results:**

The study was terminated early due to slow recruiting (31 enrolled patients: 22 ancrod, 9 placebo). A significant improvement of hearing loss was registered in both groups (ancrod: − 14.3 dB ± 20.4 dB, − 39.9% ± 50.4%; placebo: − 22.3 dB ± 13.7 dB, − 59.1% ± 38.0%). A statistically significant group-difference was not detected (*p* = 0.374). Placebo response of 33.3% complete and 85.7% at least partial recovery was observed. Plasma fibrinogen levels were reduced significantly by ancrod (baseline: 325.2 mg/dL, day 2: 107.2 mg/dL). Ancrod was tolerated well, no adverse drug reaction was of severe intensity, no serious adverse events occurred.

**Conclusion:**

Ancrod reduced fibrinogen levels that support its mechanism of action. The safety profile can be rated positively. Since the planned number of patients could not be enrolled, no efficacy conclusion can be drawn. The high rate of placebo response challenges clinical trials for SSHL and needs to be considered in future investigations.

***Trial registrations***

This study was registered in the EU Clinical Trials Register, EudraCT-No. 2012-000066-37 at 2012-07-02.

**Supplementary Information:**

The online version contains supplementary material available at 10.1007/s00405-023-07896-z.

## Introduction

Sudden sensorineural hearing loss (SSHL) is a frequent inner ear disorder with a wide-ranging incidence (USA: 5-27/100,000; Germany: 160/100,000) probably due to a high rate of spontaneous recovery before seeking medical attention [1–3]. Corticosteroids constitute the standard therapy, even though level of evidence is low [4]. Etiology remains unclear in the majority of cases, 71% are ultimately classified as idiopathic. Moreover, the underlying pathomechanism is still not completely understood, proposed theories include infectious causes, autoimmune disorders, or microcirculatory disturbances [5]. Impairment of the cochlear microcirculation was discussed to be relevant due to its sudden and single sided occurrence similar to a central retina vein occlusion of obvious vascular origin [6]. A vascular origin has been postulated as either the primary cause or part of a multifactorial genesis. Moreover, impaired cochlear microcirculation may be the final common pathogenic pathway of other etiological factors. Hyperfibrinogenemia was identified as a risk factor for SSHL [7]. Fibrinogen, a large glycoprotein influences rheologic properties by increasing viscosity and promoting aggregation of erythrocytes and thrombocytes [7–10]. An increased blood viscosity in SSHL-patients was not only confirmed as a potential etiopathological factor, but also discussed as a promising therapeutic target [7, 11]. Reducing plasma-fibrinogen by fibrinogen/LDL-apheresis was demonstrated beneficial in clinical studies [12–14]. Inner ear microcirculation cannot be investigated in the living human. A guinea pig animal model demonstrated a reduction of cochlear blood flow caused by hyperfibrinogenemia leading to hearing loss. Conversely, drug-induced reduction of elevated fibrinogen levels by ancrod caused an increase in cochlear blood flow and recovery of acute hearing loss [15, 16]. The snake venom thrombin-like enzyme ancrod cleaves fibrinogen without activating factor XIII, generating soluble fibrin polymers rapidly digested by plasmin and thus eliminated from the circulation via the reticuloendothelial system [17, 18].

The aim of this randomized placebo-controlled trial was to evaluate the efficacy of drug-induced defibrinogenation by ancrod as primary treatment in patients with SSHL.

## Materials and methods

### Participants

The study was approved by the responsible Ethics Committees of the participating centers (approval number of the leading Ethics Committee of the University Medical Center Göttingen 14/7/12). It was funded and the investigational medical product (IMP) was provided by the sponsor of this clinical trial, the Nordmark Pharma GmbH. The study was performed in accordance with the Declaration of Helsinki, version 10/2008. The complete study protocol and statistical analysis plan is available in Supplemental Material 1. All participants gave written informed consent. This study was registered in the EU Clinical Trials Register, EudraCT-No. 2012-000066-37 at 2012-07-02.

Inclusion criteria comprised male or female patients aged 18–70 years presenting with acute (< 7d after onset), untreated unilateral idiopathic SSHL ≥ 30 dB in at least 2 consecutive frequencies or ≥ 20 dB in 3 consecutive frequencies and not greater than 90 dB based upon evaluation of 8 frequencies, 0.125, 0.25, 0.5, 1, 2, 4, 6, and 8 kHz, compared to the contralateral ear. Symmetric hearing according to patient’s recollection before onset was required. Amongst others, exclusion criteria were any pre-treatment of SSHL-related hearing loss within the preceding 30 days, e.g. with steroids, or current medication that interferes with coagulation, like anticoagulants or antiplatelet drugs. All in- and exclusion criteria are listed in detail in Supplemental Material 1, page 31–32.

### Study design

This was a randomized, double-blind, multi-center, placebo-controlled, parallel-group phase II proof-of-concept study on efficacy, safety, and tolerability comparing ancrod with placebo (2:1 randomization) as primary treatment in patients with unilateral SSHL. The study was initiated in 19 German and 5 Czech sites. Thereof, in 8 sites patients were enrolled. Treatment and assessments were performed in an outpatient setting including 7 visits and a study duration for each patient of 90 days.

The primary outcome measure was the change in pure tone audiogram air conduction thresholds in the affected ear from screening/day 1 until day 8. The pure tone average (PTA) was calculated as the arithmetic mean of air conduction thresholds at affected consecutive frequencies within 0.125, 0.25, 0.5, 1, 2, 4, 6, and 8 kHz. A non-affected frequency within two affected frequencies was included. Secondary outcome measures included: change in speech audiometry, fibrinogen concentration, biomarkers (TNF-α, CD38+ , CD40+ cells), patient and physician assessment of change in hearing impairment, and tinnitus severity.

### Assessments and interventions

Screening, enrollment, and randomization needed to be performed within a 36 h period. Audiometric tests at time of screening and after treatment (day 8) included pure tone and speech audiometry (specific national test per country to determine the dB hearing level (dB HL) value where 50% of single words will be understood; Germany: German language Freiburg Monosyllabic Test). Hearing impairment, and the occurrence and the degree of tinnitus was moreover assessed by means of a 11-item numeric rating scale (NRS) from 0 to 10. Laboratory tests before study drug administration included plasma fibrinogen concentration.

Patients received the initial infusion of the IMP at day 1 (0.167 IU ancrod/kg bw/h or placebo). The dose was adjusted based on the screening fibrinogen concentration: 2 h infusion if screening fibrinogen concentration was ≥ 180 and ≤ 360 mg/dl, corresponding to a total dose of 0.33 IU/kg bw; 3 h infusion if screening fibrinogen concentration was > 360 mg/dl, corresponding to a total dose of 0.50 IU/kg bw. On day 2, again fibrinogen concentration was assessed to decide on proceeding with defibrinogenation treatment. If the fibrinogen concentration was < 50 mg/dl, the patient was taken off treatment. If it was ≥ 50 mg/dl, treatment proceeded with subcutaneous administration of either ancrod (1 IU/kg bw) or placebo on day 2, 4, and 6. This regimen was shown to be effective in defibrinogenation and was accompanied with the lowest incidence of bleeding complications [19].

On day 8 the treatment effect was evaluated by above mentioned tests and during follow-up at day 30 and 90. Blood samples were taken for coagulation diagnostics, biomarkers, and neutralizing anti-ancrod antibodies at defined time points throughout the clinical trial.

### Statistical analysis

The statistical analysis plan is available in Supplemental Material 1.

Based on 2-sided *t* tests it was calculated that 87 evaluable patients should be treated to confirm a treatment difference of 15 dB PTA value of active treatment versus placebo with a 2:1 randomization, an α-level of 5%, and a statistical power of 90%, assuming a standard deviation of 20%. To compensate for dropouts, 99 patients were planned to be enrolled. Due to slow recruitment the study was terminated early after enrollment of 37 patients and the collected data was analyzed.

The primary analysis is based on the intention-to-treat population (full analysis set; FAS); in addition, a per-protocol analysis (excluding patients not fulfilling the evaluability criteria; per-protocol set, PPS) was performed. Evaluability criteria were: SSHL as defined per inclusion criterion; valid PTA data for day 1 and 8; adhere reasonably well to the study protocol without major protocol deviations.

Quantitative efficacy variables (including the primary efficacy variable) were analyzed by means of an analysis of covariance (ANCOVA) based on the generalized linear model with treatment group (fixed factor) and the covariates baseline value and country (study centers were pooled according to an external factor: country). For the primary efficacy variable, confirmative testing was performed aiming to show superiority in favor of ancrod over placebo. Qualitative parameters were analyzed with Fisher's exact test (2 × 2-tables) or the Freeman-Halton test (tables larger than 2 × 2). A two-sided 5% significance level was applied. All other variables were compared between treatment groups in an explorative manner.

Subgroups were evaluated descriptively for the primary efficacy endpoint. Subgroup analyses and a step-down ANCOVA were applied to identify risk or influential factors.

Safety data were analyzed descriptively by treatment group. Absolute and relative frequencies were calculated for adverse events (AEs) by system organ class and preferred term. AE rates were compared between the treatment groups by Fisher’s exact test.

Statistical analyses were performed using the SAS^®^ version 9.3 (Statistical Analysis System, SAS Institute, Cary, NC, USA).

## Results

### Patients, demographics, and baseline values

**Fig. 1:**
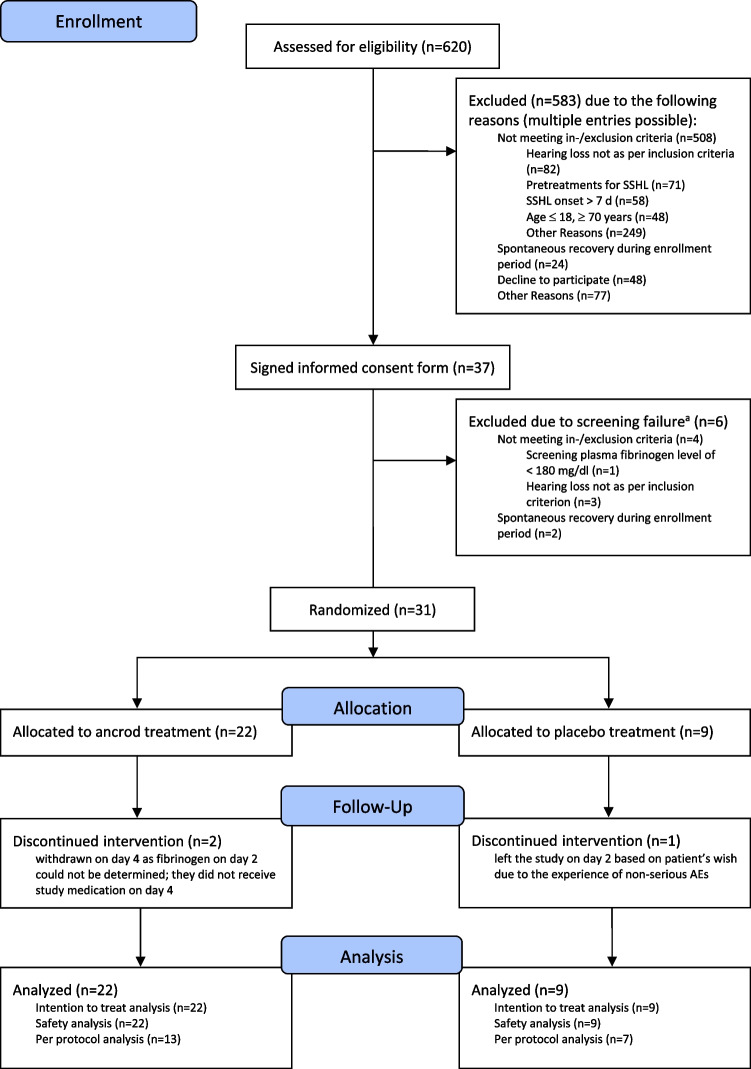
Consolidated Standards of Reporting Trials Flow Diagram. ^a^Screening failure was defined as a patient having signed the informed consent form, but discontinuing before being randomized

The progress of patients throughout the trial is reported by the consolidated standards of reporting trials diagram (Fig. 1). 620 patients were screened for eligibility from 08/2013 to 09/2018. The most frequent causes for exclusion were hearing loss not according to inclusion criteria (*n* = 82), pretreatment of SSHL (*n* = 71), and SSHL onset > 7d (*n* = 58). Eligibility assessment was accomplished within 36 h. During that period, 24 patients presented with spontaneous recovery the other day. Thirty-seven patients were enrolled into the trial. Of these, six patients were excluded after already having signed informed consent due to screening failure since they did not meet the in-/exclusion criteria (*n* = 4) or again presented with spontaneous recovery during enrollment period (*n* = 2). Thirty-one patients (83.8%) were randomized to study treatment. The study was terminated early as the planned 99 patients could not be enrolled in a reasonable time period.

Twenty-two patients (71.0%) received ancrod and 9 (29.0%) placebo. These 31 cases were analyzed according to the intention-to-treat approach (FAS). For demographic data and baseline characteristics see Table 1. With regard to the per-protocol analysis, 20 cases were included in the PPS since 11 were excluded due to at least one major protocol deviation. These were: in-/exclusion criteria violation (*n* = 2), deviation in the audiological measurement regarding primary outcome measure (*n* = 4), study medication not administered as stipulated by study protocol (*n* = 8). Multiple reasons were possible per patient. In total, 9/22 (40.9%) ancrod and 2/9 (22.2%) placebo patients had at least one major protocol deviation.

**Table 1:** Demographic data and baseline characteristics

	Ancrod (*n* = 22)	Placebo (*n* = 9)	All (*n* = 31)
Gender			
Male, *n* (%)	11 (50.0)	4 (44.4)	15 (48.4)
Female, *n* (%)	11 (50.0)	5 (55.6)	16 (51.6)
Age, mean ± SD (median), years	50.4 ± 11.3 (51.5)	54.1 ± 11.5 (58.0)	51.5 ± 11.3 (53.0)
BMI, mean ± SD (median), kg/m^2^	28.34 ± 5.85 (26.88)	26.32 ± 3.76 (25.20)	27.73 ± 5.33 (26.40)
Duration of SSHL before first presentation, mean ± SD (median), days	3.6 ± 1.8 (3.0)	4.3 ± 1.9 (5.0)	3.8 ± 1.8 (3.0)
Tinnitus at screening, *n* (%)	17 (77.3)	9 (100)	26 (83.9)
SSHL type, *n* (%)			
Low-tone	8 (36.4)	3 (33.3)	11 (35.5)
High-tone	7 (31.8)	3 (33.3)	10 (32.3)
Pantonal	5 (22.7)	2 (22.2)	7 (22.6)
Unspecific	0 (0.0)	1 (11.1)	1 (3.2)
Not assessable	2 (9.1)	0 (0.0)	2 (6.5)

*BMI* body mass index; *SSHL* sudden sensorineural hearing loss; *SD* standard deviation

### Efficacy

The PTA before treatment was 35.6 ± 15.0 dB HL in the ancrod and 38.6 ± 10.4 dB HL in the placebo group (FAS). From baseline to day 8 (end of treatment), a treatment effect was observed in both groups. When adjusting for the covariates baseline PTA and country, the estimates for the treatment effect (least square means) achieved values of − 14.92 dB (CI [− 23.37, − 6.46]) for ancrod and -22.25 dB (CI [− 36.82, − 7.67]) for placebo with statistical significance (*p* = 0.05). The difference between the two study groups (ancrod-placebo) was estimated at 7.33 dB with 95% CI [− 9.41, 24.06]. A statistically significant difference could not be detected (*p* = 0.37). The corresponding analyses on the PPS is in line with these results (Table 2). Subgroup analyses did not identify any risk or influential factor on the study result (data not shown). The PTA over time of the intention-to-treat and PPS are shown in Table 3. Categorical change of SSHL from baseline to day 8 revealed a response rate of 50.0% (PPS: 66.7%) within ancrod and 85.7% (PPS: 85.7%) within placebo treated patients (placebo response).

**Table 2:** Changes of pure tone average from screening to day 8 (end of treatment)

	Full analysis set (*n* = 31)		Per protocol set (*n* = 20)	
Treatment	Ancrod (*n* = 20)^b^	Placebo (*n* = 7)^b^	Ancrod (*n* = 13)^b^	Placebo (*n* = 7)^b^
Screening (Baseline)^a^, Mean ± SD [Median], dB HL	35.61 ± 15.03 [35.75]	38.59 ± 10.40 [39.00]	40.57 ± 13.16 [41.25]	38.59 ± 10.40 [39.00]
Absolute change from Screening to Day 8 (End of Treatment)^b^, Mean ± SD [Median], dB	− 14.25 ± 20.37 [− 7.00]	− 22.29 ± 13.68 [− 26.71]	− 19.83 ± 20.28 [− 20.00]	− 22.29 ± 13.68 [− 26.71]
Relative change from Screening to Day 8 (End of Treatment), Mean ± SD [Median], %	− 39.94 ± 50.39 [− 37.99]	− 59.07 ± 38.04 [− 68.50]	− 47.32 ± 47.73 [− 47.73]	− 59.07 ± 38.04 [− 68.50]

*HL* hearing level

^a^Pure tone average (PTA) of the affected frequencies

^b^Changes of the PTA values from Screening to day 8/end of Treatment for those patients where all data are available (“complete cases”)

**Table 3:** Pure tone average in the affected ear over time

	Full analysis set (*n* = 31)		Per protocol set (*n* = 20)	
Treatment	Ancrod (*n* = 22)	Placebo (*n* = 9)	Ancrod (*n* = 13)	Placebo (*n* = 7)
Screening (Baseline)^a^, Mean ± SD, dB HL	35.80 ± 14.37 (*n* = 22)	36.57 ± 9.86 (*n* = 9)	40.57 ± 13.16 (*n* = 13)	38.59 ± 10.40 (*n* = 7)
Day 8 (End of Treatment)^a^, Mean ± SD, dB HL	21.36 ± 19.63 (*n* = 20)	16.30 ± 16.72 (*n* = 7)	20.74 ± 19.12 (*n* = 13)	16.30 ± 16.72 (*n* = 7)
Day 30^a^, Mean ± SD, dB HL	18.21 ± 21.01 (*n* = 21)	11.92 ± 16.12 (*n* = 8)	17.16 ± 22.48 (*n* = 13)	13.34 ± 16.87 (*n* = 7)
Day 90^a^, Mean ± SD, dB HL	15.97 ± 20.53 (*n* = 21)	10.08 ± 13.85 (*n* = 8)	14.93 ± 21.34 (*n* = 13)	11.37 ± 14.43 (*n* = 7)

*HL* hearing level

^a^Pure tone average (PTA) of the affected frequencies

Plasma fibrinogen concentration was reduced significantly by ancrod, whereas placebo treatment did not show a comparable effect (*p* < 0.0001). With regard to biomarkers (TNF-α, CD38+ , CD40+ cells), there was no relevant difference between the study groups and no correlation of a clinical effect of IMP (data not shown). Assessment of hearing impairment via NRS revealed a significant treatment effect from baseline to day 8 in both groups without any significant difference between the two study groups. Results of this assessment rated by the physician revealed comparable results (data not shown). The results of the secondary efficacy endpoints, including speech audiometry are depicted in Table 4.

**Table 4:** Categorical Change of SSHL and Secondary Efficacy Endpoints

	Full analysis set (*n* = 31)		Per protocol set (*n* = 20)	
Treatment	Ancrod (*n* = 22)	Placebo (*n* = 9)	Ancrod (*n* = 13)	Placebo (*n* = 7)
Categorical change of SSHL^a^				
Recovery, n (%)	4 (18.2)	3 (33.3)	4 (30.8)	3 (42.9)
Good improvement	3 (13.6)	2 (22.2)	2 (15.4)	2 (28.6)
Fair improvement	2 (9.1)	1 (11.1)	2 (15.4)	1 (14.3)
No change	3 (13.6)	1 (11.1)	0 (0.0)	1 (14.3)
Worsened hearing	6 (27.3)	0 (0.0)	4 (30.8)	0 (0.0)
N/A	4 (18.2)	2 (22.2)	1 (7.7)	0 (0.0)
Freeman-Halton test	*p* = 0.4654		*p* = 0.4382	
Response rate (N/A cases excluded), *n* (%)^b^	9/18 (50.0)	6/7 (85.7)	8/12 (66.7)	6/7 (85.7)
Changes in fibrinogen level				
Screening (Baseline), Mean ± SD [Median], mg/dl	325.3 ± 59.2 [330.5], (*n* = 20)	360.6 ± 102.8 [368.0], (*n* = 8)	N/A	N/A
Change from Screening to Day 8 (End of Treatment), Mean ± SD [Median], mg/dl	− 180.2 ± 62.9 [− 174.5], (*n* = 20)	− 52.8 ± 70.4 [− 33.5], (*n* = 8)	N/A	N/A
Difference in estimates for treatment effect (ancrod-placebo)	− 143.99 mg/dL, 95% CI [− 193.55, − 94.44]			
Fibrinogen level over time				
Screening (Baseline), Mean ± SD	325.2 ± 56.3 (*n* = 22)	367.8 ± 98.5 (*n* = 9)	N/A	N/A
Day 2, Mean ± SD	107.2 ± 59.1 (*n* = 19)	331.6 ± 83.4 (*n* = 9)	N/A	N/A
Day 8 (End of Treatment), Mean ± SD	145.0 ± 58.4 (*n* = 20)	307.9 ± 83.7 (*n* = 8)	N/A	N/A
Patient assessment of hearing impairment (NRS)				
Screening (Baseline), Mean ± SD	5.9 ± 2.0 (*n* = 22)	5.9 ± 2.0 (*n* = 9)	N/A	N/A
Day 8 (End of Treatment), Mean ± SD	3.3 ± 2.7 (*n* = 21)	2.5 ± 1.9 (*n* = 8)	N/A	N/A
Day 30, Mean ± SD	2.1 ± 2.6 (*n* = 21)	2.0 ± 1.5 (*n* = 8)	N/A	N/A
Day 90, Mean ± SD	2.1 ± 2.7 (*n* = 21)	2.3 ± 2.9 (*n* = 8)	N/A	N/A
Patient assessment of tinnitus severity (NRS)				
Screening (Baseline), Mean ± SD	4.3 ± 3.2 (*n* = 22)	5.1 ± 1.5 (*n* = 9)	N/A	N/A
Day 8 (End of Treatment), Mean ± SD	2.9 ± 3.0 (*n* = 21)	2.6 ± 1.8 (*n* = 8)	N/A	N/A
Day 30, Mean ± SD	2.2 ± 3.2 (*n* = 21)	1.3 ± 1.6 (*n* = 8)	N/A	N/A
Day 90, Mean ± SD	1.5 ± 2.7 (*n* = 21)	1.0 ± 2.1 (*n *= 8)	N/A	N/A

Change in speech audiometry (German cohort only)^c^				
Treatment	Ancrod (*n* = 13)	Placebo (*n* = 6)	Ancrod (*n* = 6)	Placebo (*n* = 5)
Percentage of words understood out of 40 (affected ear) at 60 dB HL				
Screening (Baseline), Mean ± SD [Median], %	60.45 ± 33.43 [70.00], (*n* = 11)	1.00 ± 2.24 [0.00], (*n* = 5)	53.33 ± 41.19 [52.50], (*n* = 6)	1.00 ± 2.24 [0.00], (*n* = 5)
Change from Screening to Day 8 (End of Treatment), Mean ± SD [Median], %	19.09 ± 33.75 [5.00], (*n* = 11)	65.00 ± 37.91 [80.00], (*n* = 5)	29.17 ± 44.54 [10.00], (*n* = 6)	65.00 ± 37.91 [80.00], (*n* = 5)
Percentage of words understood out of 40 (affected ear) at 80 dB HL				
Screening (Baseline), Mean ± SD [Median], %	78.89 ± 31.50 [85.00], (*n* = 9)	45.00 ± 35.88 [45.00], (*n* = 5)	66.25 ± 44.98 [82.50], (*n* = 4)	45.00 ± 35.88 [45.00], (*n* = 5)
Change from Screening to Day 8 (End of Treatment), Mean ± SD [Median], %	20.00 ± 30.41 [15.00], (*n* = 9)	37.00 ± 24.14 [30.00], (*n* = 5)	31.25 ± 43.85 [17.50], (*n* = 4)	37.00 ± 24.14 [30.00], (*n* = 5)
Loudness with which 50% of digits are understood (affected ear)				
Screening (Baseline), Mean ± SD [Median], dB	29.73 ± 23.02 [20.00], (*n* = 11)	45.80 ± 12.50 [42.00], (*n* = 5)	37.33 ± 29.87 [36.00], (*n* = 6)	45.80 ± 12.50 [42.00], (*n* = 5)
Change from Screening to Day 8 (End of Treatment), Mean ± SD [Median], dB	− 12.82 ± 24.06 [− 3.00], (*n* = 11)	− 26.60 ± 10.85 [− 25.00], (*n* = 5)	− 20.83 ± 31.28 [− 7.50], (*n* = 6)	− 26.60 ± 10.85 [− 25.00], (*n* = 5)

*HL* hearing level; *NRI* numeric rating scale; *SSHL* Sudden sensorineural hearing loss; *SD* standard deviation

^a^Values from Screening to Day 8/End of Treatment. Only the frequencies affected by SSHL among the frequencies 250 Hz, 500 Hz, 1 kHz, 2 kHz and 4 kHz are included for assessment of improvement (as the best of following categories): Recovery = Hearing level recovers within 30 dB at 250, 500, 1000, 2000 Hz and within 25 dB at 4000 Hz, or hearing level recovers to that of contralateral intact ear if this is stable, i.e. value for day 8 differs not more than 10 dB from value for screening. Good improvement = Average hearing improvement is ≥ 30 dB. Fair improvement = Average hearing improvement is ≥ 10 dB and ≤ 30 dB. No change = Average hearing improvement is ≤ 10 dB. Worsened hearing = In average, higher threshold values are seen at day 8 than at screening. (Hearing recovery as defined by the Ad Hoc Committee of the Ministry of Health and Welfare in Japan). N/A = No SSHL at study entry, or none of the five frequencies were affected by SSHL at screening, or no PTA at day 8. The Freeman-Halton test is a multidimensional extension of Fisher's Exact test. Values N/A were ignored for testing

^b^post-hoc analysis. Calculation by summing up the cases with “recovery”, “good improvement”, and “fair improvement” divided by the total number of cases with data available (“N/A” cases excluded)

^c^Values from Screening to day 8/end of Treatment for those patients where all data are available (“complete cases”)

Taken together, pure tone and speech audiometry, and assessments on hearing impairment and tinnitus showed a treatment effect in both study groups without revealing superiority of the ancrod treatment.

### Safety

The frequency of observed AEs by patient was 15/22 (68.2%) in the ancrod and 5/9 (55.6%) in the placebo group with no significant difference (*p* = 0.683). The number of patients with AEs causally related to IMP is low: 6 ancrod (27.3%) and 2 (22.1%) placebo patients (*p* = 1.00). AEs causally related to IMP occurred at most for one patient in each treatment group. The AEs with a causal link to IMP and occurring in more than one patient is “Feeling hot” (*n* = 2), one in each study group. Moreover, due to its mode of action, a higher occurrence of bleedings was expected in the ancrod group. Accordingly, two cases of mild bleeding (hematoma, vaginal hemorrhage) were reported. These AEs were rated causally related to IMP. The patient with hematoma discontinued treatment with ancrod but completed the study. One patient in the placebo group discontinued the study due to non-serious AEs. A summary of AEs with absolute and relative frequencies by system organ class and preferred term is provided in the Supplemental Material 2.

No serious adverse event was reported. Just one AE was rated severe: this patient had received ancrod and the AE (unilateral deafness) was classified unrelated to effects of the study medication by the investigator. This was most likely progression of the disease under study, but was classified as AE. All other AEs were rated with mild or moderate intensity.

Except fibrinogen no clinically significant changes occurred in results of routine laboratory analyses, physical examination, and vital signs during treatment. One ancrod patient was tested positive for neutralizing anti-ancrod antibodies at screening and subsequently tested negative at day 8 and 30. At day 30, two patients were tested positive that had been tested negative at screening and day 8.

## Discussion

This randomized controlled trial on drug-induced defibrinogenation for SSHL did not demonstrate a significant difference between ancrod and placebo with respect to the primary outcome measure. Unfortunately, no conclusion regarding efficacy can be drawn, since the study was terminated ahead of schedule.

The safety results show that ancrod was well tolerated. No serious adverse event was reported. Adverse drug reactions are within the expected range. Laboratory parameters show an expected decline in plasma fibrinogen in the ancrod group while remaining on a stable level in the placebo group. This observation supports ancrod’s mechanism of action and the dosing schedule. Ancrod was already marketed in Europe and Canada for indications like peripheral arterial occlusive disease, deep vein thrombosis, and prophylaxis for thromboembolism. It is easy to monitor by routine laboratory tests measuring plasma fibrinogen.

The observed reduction of plasma fibrinogen by ancrod is in line with in vivo guinea pig studies, in which this drug-induced reduction of elevated fibrinogen levels resulted in an increase of cochlear blood flow accompanied by recovery of acute hearing loss [15]. The effect of defibrinogenation therapy on cochlear microcirculation cannot be investigated in patients. However, the strategy of reducing plasma-fibrinogen as a therapeutic target in SSHL was supported by former studies investigating the treatment with the snake venom thrombin-like enzyme batroxobin compared to systemic corticosteroid application: Kubo et al*.* reported a higher recovery rate (57.3% versus 38.7%) and greater improvement of other symptoms closely related to recovery of the inner ear, such as tinnitus and aural fullness [20]. Suzuki et al*.* found the overall hearing outcomes similar to high-dose steroid therapy, but according to subgroup analysis they recommended steroids for patients with moderate and defibrinogenation therapy for those with severe hearing loss [21]. In line, post-hoc analysis of high-dose steroid versus batroxobin treatment led to the conclusion that defibrinogenation therapy should be chosen specifically for patients with profound hearing loss and initial high fibrinogen [22]. An open study, investigating batroxobin in combination with low-molecular dextran, vasodilators, and vitamins found a correlation between hearing recovery and onset of treatment [23]. This poses the question of a relevance of timely administration within the course of defibrinogenation treatment versus the effect of spontaneous recovery.

Drug-induced enzymatic defibrinogenation has the advantage of an easy, cost-effective, and widely accessible treatment. Previous studies investigated more elaborate extracorporeal techniques to prove the value of defibrinogenation in SSHL-treatment. A superior beneficial effect of fibrinogen/LDL-apheresis in comparison to the standard therapy of high-dose steroids was demonstrated in clinical studies [13, 14]. Moreover, non-responders of standard therapies significantly improved after receiving fibrinogen/LDL-apheresis as second-line therapy, even though the timeframe of spontaneous remissions was exceeded [12]. Likewise, rheopheresis, which reduces a defined spectrum of rheologically relevant high molecular weight proteins from plasma, including fibrinogen, showed to be effective in a case series of SSHL patients [24]. Additionally, a large study comparing this procedure to i.v. corticosteroids or hemodilution demonstrated an equal efficacy [25], and in cases of recurrent SSHL that was refractory to infusion therapy rheopheresis still achieved improvement [26]. Lastly, in an uncontrolled pilot study a single procedure of specific fibrinogen apheresis achieved complete remission of hearing loss in 60% and in another 20% at 4 weeks post-onset [27]. In summary, lowering plasma fibrinogen levels displays a promising therapeutic target and fibrinogen is of potential importance in the pathophysiology of SSHL.

However, conducting and interpreting clinical studies on SSHL is challenging, especially under the terms of inherent high rates of spontaneous recovery. Rates of complete recovery range from 35 to 68% [28, 29], even though these derive from studies which were performed 40 years ago. More recent studies report placebo response rates of 26% complete recovery [30, 31]. Rates of partial recovery were stated between 51 and 89% (placebo or untreated patients) [28, 29, 32, 33]. Spontaneous recovery may be more pronounced in mild-to-moderate affected SSHL patients than in patients with SSHL > 60 dB HL [31]. In line, a high placebo response of 33.3% complete and 85.7% at least partial recovery was observed in the present study. Moreover, 4.2% of the 620 screened patients recovered during the 36 h enrollment period. Considering the placebo-controlled design the present study might have had better prerequisites than other randomized trials that tested against e.g., the therapeutic standard, systemic steroids. These results challenge any study design regarding SSHL and consequently, a considerably stronger effect is required to reach a significant difference in a randomized controlled trial. This needs to be preconceived in future clinical trials.

The study was terminated early due to insufficient recruitment. This is probably caused by (1) demanding in-/exclusion criteria: After 5 years and eligibility screening of 620 patients with acute hearing loss, only 37 of the initially planned 99 patients were enrolled, of which 31 were randomized (5%). Furthermore, considering these highly selective criteria an adequate representation of “common” SSHL can be called into question. (2) Involvement of predominantly tertiary referral centers: The most frequently constituted exclusion criteria were “pretreated SSHL” (*n* = 71) and “onset > 7 days ago” (*n* = 58), which lead to presumption of potentially higher recruitment numbers, if rather primary and secondary care providers have been involved. (3) High complexity of study protocol and logistics: This is reflected by the observation of a high rate of major protocol deviations (35.5%) despite several investigator meetings and continuous monitoring. In order to increase inclusion numbers several efforts were made e.g., reports in local press, advertisement in public transport, online presence, Google AdWords.

### Strengths and limitations

The strength of this study is the placebo-controlled design displaying the high placebo response rate. The major limitation is the early study termination due to patient recruitment difficulties that does not allow drawing conclusions on efficacy. Consequently, other study limitations are the demanding in- and exclusion criteria, and the complex study protocol and logistics that resulted in the involvement of predominantly tertiary referral centers. In these limitations, on the contrary, lies a strength of the study: the lessons learned allow investigators to draw conclusions for designing future study protocols on SSHL.

## Conclusions

Ancrod reduced fibrinogen levels supporting its mechanism of action. The safety profile can be rated positive. The planned number of patients could not be enrolled; therefore, no efficacy conclusion can be drawn. A distinct improvement of SSHL occurred in both study arms. The high placebo response rate in SSHL challenges clinical trials and needs to be considered in future investigations. Conclusions about in- and exclusion criteria, high complexity of the study protocol and logistics, and involvement of primary and secondary care providers may provide benefit for future studies.

## Supplementary Information

Below is the link to the electronic supplementary material.

Supplementary file1 Study protocol and statistical analysis plan (PDF 2315 KB)

Supplementary file2 Adverse events with absolute and relative frequencies by system organ class and preferred term (PDF 120 KB)

## Data Availability

Underlying research materials, the full trial protocol, and raw data related to this work can be obtained on reasonable request from the corresponding author.
