# Supplementary material for: Randomized, placebo-controlled study on efficacy, safety and tolerability of drug-induced defibrinogenation for sudden sensorineural hearing loss: the lessons learned
Source: Eur Arch Otorhinolaryngol. 2023 Mar 7;280(9):4009–18. doi: 10.1007/s00405-023-07896-z (PMC10382375; doi:10.1007/s00405-023-07896-z)
Supplement: Supplementary file 1 — Supplementary file1 Study protocol and statistical analysis plan (PDF 2315 KB) [file 405_2023_7896_MOESM1_ESM.pdf]

Clinical Study Protocol

Double-blind, randomized, placebo-controlled study on efficacy, safety and tolerability of ancrod in patients with sudden sensorineural hearing loss (SSHL)

Nordmark Study No: NM-V-101

EudraCT No: 2012-000066-37

Version Number and Date including Amendments:

Protocol Amendment # 7.1

15.02.2018

Including Protocol Amendment # 7.0	19 July 2017
Including Protocol Amendment # 6.0	12 January 2017
Including Protocol Amendment # 5.0	28 July 2016
Including Protocol Amendment # 4.2 (CR)	28 April 2015
Including Protocol Amendment # 4.1	10 February 2015
Including Protocol Amendment # 3.2	19 June 2013
Including Protocol Amendment # 3.1	20 March 2013
Including Protocol Amendment # 2.0	08 January 2013
Including Protocol Amendment # 1.0	15 October 2012
Including Protocol final # 2.0	08 May 2012

Sponsor:

xxxxx

Confidential
The information contained in this document is the property of Nordmark Arzneimittel GmbH & Co. KG. The content of this document may not be disclosed to others than the investigator(s) and duly staff, applicable EC/IRB and regulatory authorities without written approval from Nordmark Arzneimittel GmbH & Co. KG, except to the extent necessary to obtain informed consent from those persons to whom the medication may be administered.

Statement of Compliance:

This study will be conducted in compliance with the protocol, International Conference on Harmonization Good Clinical Practice E6 (ICH-GCP), and the applicable laws and regulatory requirements.

Signature page 1

The signatures below constitute the approval of this protocol and the attachments, and provide the necessary assurances that this study will be conducted according to all stipulations of the protocol, including all statements regarding confidentiality, and according to local legal and regulatory requirements and ICH guidelines.

Signature page 2

Study Center

Additional Study Personnel / Site Information:

**Pharmacovigilance Nordmark:**	**IP Production Nordmark (Qualified Person):**
Name, degree, title:	Name, degree, title:
Institution Name:	Institution Name:
Address:	Address:
Phone Number:	Phone Number:
Fax Number:	Fax Number:
E-Mail:	E-Mail:
	**CRO (Clinical Operations) – Czech Republic:**
Name, degree, title:	Name, degree, title:
Institution Name:	Institution Name:
Address:	Address:
Phone Number:	Phone Number:
Fax Number:	Fax Number:
E-Mail:	E-Mail:
	**Central Laboratory**
Name, degree, title:	Name, degree, title:
Institution Name:	Institution Name:
Address:	Address:
Phone Number:	Phone Number:
Fax Number:	Fax Number:
E-Mail:	E-Mail:

Additional Study Personnel / Site Information (*continued*):

**CRO (Randomization and Blinding Procedures):**	
Name, degree, title:	
Institution Name:	
Address:	
Phone Number:	
Fax Number:	
E-Mail:	

Table of Contents:

**Protocol Synopsis**		**11**
**1**	**Abbreviations and Definition of Terms**	**17**
**2**	**Background Information**	**19**
2.1	Introduction	19
2.2	Study Rationale	20
2.3	Dosing Rationale	22
2.4	Potential Risks and Benefits	22
2.4.1	Potential Risks	22
2.4.2	Potential Benefits	23
**3**	**Design, Objectives and Endpoints**	**24**
3.1	Study Design	24
3.2	Objectives and Endpoints	26
3.2.1	Primary Outcome Measure	26
3.2.2	Secondary Outcome Measures	26
**4**	**Schedule of Events and Study Duration**	**27**
4.1	Screening (Day 1, Visit 1)	27
4.2	Visits of the Double-Blind Treatment Phase (Days 2, 4, 6 and 8)	28
4.3	Follow-up Visits at Day 30 and Day 90 (± one week)	29
4.4	Final Study Visit at Day 90 (± one week)	30
4.5	Early Termination Visit	30
4.6	Unscheduled Visit	30
4.7	Duration of the study	30
**5**	**Patients**	**30**
5.1	Study Population	30
5.1.1	Patients and Characteristics	30
5.1.2	Inclusion Criteria	31
5.1.3	Exclusion Criteria	32
5.1.4	Withdrawal of Patients	34
5.1.5	Patient Log	34
**6**	**Study Drug and Related Procedures**	**34**
6.1	Study Product	35
6.1.1	Formulation, Packaging, and Labelling	35
6.1.2	Preparation, Administration, Storage, and Dosage of Study Product	36

6.2	Reference Product	38
6.3	Concomitant Medication	38
6.4	Precautionary and Prohibited Medications and Procedures	38
6.4.1	Prohibited Medications and Procedures	38
6.4.2	Precautionary Medications and Procedures	39
6.4.3	Prophylactic Medications and Procedures	39
6.5	Rescue Medications	39
6.6	Randomization and Blinding Procedures	39
6.6.1	Maintenance of Study Treatment Randomization Codes	40
6.6.2	Additional Measures to Minimize/Avoid Bias	41
6.7	Drug Accountability	41
**7**	**Therapy**	**41**
7.1	Treatment Schedule	41
7.2	Compliance	42
7.3	Continuation of Therapy after the End of the Study	42
**8**	**Assessments, Materials and Methods**	**42**
8.1	Clinical Assessments	42
8.1.1	Efficacy Assessments	42
8.1.2	Safety Assessments	43
8.2	Laboratory Assessments	45
8.2.1	Efficacy Assessments	45
8.2.2	Safety Assessments	45
8.2.3	Special Assays or Procedures	46
8.2.4	Biohazard Containment	46
8.2.5	Specimen Preparation, Handling and Shipping	46
8.2.6	Instructions for Specimen Storage and Shipment	46
**9**	**Safety Assessments**	**47**
9.1	Adverse Events	47
9.1.1	Definition of an Adverse Event (AE) and Adverse Drug Reaction (ADR)	47
9.1.2	Definition of a Serious Adverse Event (SAE)	48
9.1.3	Adverse Event Reporting Period	48
9.1.4	Seriousness and Intensity	48
9.1.5	Causality (Relationship) Assessment	49
9.1.6	Eliciting Adverse Event Information	49
9.1.7	Reporting	50
9.1.8	Recording Instructions	50
9.1.9	Specific Serious Adverse Event Requirements	51

9.1.10 Record keeping by the Sponsor 52
9.1.11 Regulatory Reporting to the Competent Authorities and the Ethics Committee (EC) 52
9.1.12 Exposure in Utero (EIU) 52
9.1.13 Follow-Up of Adverse Events 54
9.2 Halting Rules for the Protocol 54
9.3 Stopping Rules for an Individual Participant/Cohort 55
10 Statistics 56
10.1 Sample Size Determination 56
10.2 Statistical Methods 56
10.2.1 Method to Evaluate the Primary Efficacy Measure 57
10.2.2 Expected Side Effects 59
10.2.3 Interim Analysis 59
11 Quality Control (QC) and Quality Assurance (QA) 59
11.1 Study Monitoring and Auditing 59
11.1.1 Study Monitoring and Source Data Verification 59
11.1.2 On-Site Audits 60
12 Ethical and Regulatory Considerations 60
12.1 Declaration of Helsinki and other regulations 60
12.2 Obtaining an EudraCT Number 60
12.3 Institutional Review Board (IRB) / Independent Ethics Committee (IEC) 60
12.4 Patient Information and Informed Consent 60
12.5 Liability and Insurance 61
12.6 Approval of competent authorities and Announcement to Local Authority 61
12.7 Curriculum Vitae and other Documentation 61
13 Data Handling and Record Keeping 61
13.1 Protocol Review 61
13.2 Changes to Final Study Protocol 62
13.3 Case Report Forms (CRFs) 62
13.4 Patient Data Protection 63
13.5 Record Retention 63
14 Publications 64
15 Financial Support 64
16 Reference List 65

17 List of Appendices 68

17.1 Patient Log/Identification List 69

17.2 Patient Screening and Enrollment Log 70

17.3 Signature and Delegation List/Log 71

17.4 Dosing Worksheet 73

17.5 Drug Accountability Form 74

17.6 Study Schedule 76

17.7 Key data elements for inclusion in expedited reports of serious adverse drug reactions (taken from attachment 1 of ICH Guideline E2A) 78

17.8 World Medical Association Declaration of Helsinki 80

Protocol Synopsis

TITLE:	Double-blind, randomized, placebo-controlled study on efficacy, safety and tolerability of ancrod in patients with sudden sensorineural hearing loss (SSHL)
PROTOCOL NUMBER:	NM-V-101
EUDRACT NUMBER:	2012-000066-37
IND #:	Not applicable
STUDY PHASE:	Phase II
DESIGN:	This is a randomized, double-blind, multicenter, placebo-controlled, parallel-group phase II proof-of-concept study comparing ancrod treatment versus placebo in patients with unilateral sudden sensorineural hearing loss (SSHL). Patients presenting with unilateral SSHL within 7 days after onset and meeting all in- and exclusion criteria will be randomized into 2 cohorts in a ratio of 2:1 between active treatment and placebo.<br>Study treatment and all study assessments will be performed in an outpatient setting at designated clinical study sites.
OBJECTIVES/AIM OF THE STUDY:	The aim of this study is to evaluate the efficacy of ancrod treatment on hearing ability in patients suffering from SSHL.
PRIMARY OBJECTIVE:	The primary objective of this study is to determine the efficacy of ancrod as primary treatment of SSHL compared to placebo in patients with unilateral sudden sensorineural hearing loss as determined by audiometry.
PRIMARY EFFICACY VARIABLE:	The primary outcome measure for this study is the change in pure tone audiogram (PTA) in the affected ear from screening until Day 8. The PTA will be calculated as the arithmetic mean of air conduction thresholds at the affected consecutive frequencies in the frequency range of 0.125 to 8 kHz. A non-affected frequency within two affected frequencies will be included.
SECONDARY OBJECTIVES:	The secondary objectives of this study are to evaluate:<br>– The relationship between biomarkers and clinical efficacy.<br>– The safety and tolerability of ancrod.
SECONDARY OUTCOME MEASURE	Secondary outcome measures will include:<br>– Change in word recognition score from screening to Day 8, Day 30 and Day 90.

	– Change of audiogram in the affected ear from screening to Day 30 and Day 90.<br>– Change in fibrinogen concentration from screening to Day 2 and Day 8.<br>– Change in biomarkers from screening to Day 8.<br>– Patient assessment of change in hearing impairment.<br>– Physician assessment of change in hearing impairment.<br>– Change in tinnitus severity.
STUDY POPULATION:	Male and female patients with SSHL aged 18 to 70 years, both inclusive.
NUMBER OF PATIENTS:	Up to 115 patients will be screened in order to randomize 99 patients to either ancrod or placebo in a 2:1 ratio, i.e. 66 patients treated with ancrod and 33 patients receiving placebo.
INCLUSION CRITERIA:	Patients must meet all of the following inclusion criteria in order to be eligible for this study:<br>1. Unilateral idiopathic sudden sensorineural hearing loss ≥30 dB in at least 2 consecutive frequencies in the affected ear or ≥20 dB in 3 consecutive frequencies based upon evaluation of 8 frequencies, 0.125, 0.25, 0.5, 1, 2, 4, 6, and 8 kHz, compared to the contralateral ear.<br>2. Symmetric hearing prior to onset of SSHL, according to patient's recollection.<br>3. Patients with hearing loss not greater than 90dB at 0.125, 0.25, 0.5, 1, 2, 4, 6, and 8 kHz.<br>4. Enrollment has to be accomplished within 7 days after SSHL onset.<br>5. Male or female aged ≥18 to ≤70 years.<br>6. Women of childbearing potential who are sexually active with opposite partners have to perform adequate contraception with a combination of a highly effective method of birth control and additional barrier contraception.<br>[Highly effective method of birth control is defined as those, alone or in combination, that result in a low failure rate (i.e. less than 1% per year) when used consistently and correctly for the entire study duration: combined (oestrogen and gestagen) oral contraceptives, hormone implants, hormone injectables, or hormone containing intrauterine device that needed to be in place for a period of at least 2 months prior to screening. Additional barrier contraception (at least the following methods are

	allowed: condom of the male, diaphragm with spermicide, portio cap with spermicide) has to be used for the duration of the trial, defined as from the time of screening to at least 10 days after Day 6 (Visit 4) of the double-blind treatment phase. A single barrier method is not acceptable. Women of non-childbearing potential can be included if surgically sterile (documented complete hysterectomy or bi-tubal ligations) or post-menopausal >1 year.]<br>7. Men of reproductive potential must use condoms. In addition, the female partner should also be on a safe hormonal contraception (e.g. combined oral contraceptives, hormone implants, hormone injectables or hormone containing intrauterine device) or use a barrier contraception (e.g. intrauterine device, diaphragm or portio cap with spermicide), if she is of childbearing potential.<br>8. Ability to understand and to follow the study protocol.
EXCLUSION CRITERIA:	The presence of any of the following will exclude a patient from study enrolment:<br>1. Pregnant or breast-feeding female.<br>2. positive pregnancy test before receiving study drug<br>3. Body weight >140 kg.<br>4. Bilateral SSHL.<br>5. Incomplete recovery after previous SSHL, when symmetric hearing was not reached again, according to patient's recollection.<br>6. Due to history of physical efforts suspected perilymph fistula or membrane rupture.<br>7. Previously existing, known retrocochlear hearing loss.<br>8. Any history of any ear operation or local inflammatory disease in the past one year<br>9. History of blunt or penetrating ear trauma, head trauma, barotrauma, or acoustic trauma immediately preceding SSHL.<br>10. History of Menière's disease, autoimmune hearing loss, radiation-induced hearing loss, endolymphatic hydrops.<br>11. Any pre-treatment within the preceding 30 days prior to screening, ongoing treatment or planned treatment of SSHL-related hearing loss.<br>12. History of chronic inflammatory diseases or autoimmune diseases e.g. rheumatic disease, including rheumatoid arthritis, scleroderma, lupus erythematosus, polymyalgia rheumatica, polyarteritis nodosa, temporal/giant cell arteritis, Sjögren's

	syndrome, or ulcerative colitis and Crohn's disease.<br>13. History of unstable angina, coronary artery stenting or bypass grafting within three months of enrolment, transient ischemic attacks or stroke within four weeks of enrolment.<br>14. Prior chemotherapy or treatment with immunosuppressant drugs (azathioprine, chlorambucil, cyclophosphamide, or other alkylating agents), cyclosporine, etanercept, infliximab, interferon or any therapy with drugs known as ototoxic (e.g. aminoglycosides, cisplatin, loop diuretics, quinine etc.) in the past 6 months.<br>15. Gastrointestinal pathology: lesions liable to bleed such as active peptic ulcer disease, inflammatory bowel disease, history of gastrointestinal bleeding, or recent hemorrhage at any anatomical site requiring medical intervention.<br>16. Screening plasma fibrinogen level of <180 mg/dL.<br>17. Known disorder of platelet function or coagulation abnormality.<br>18. Platelet count of $<100{,}000/mm^3$.<br>19. Severe liver disease.<br>20. Hepatic failure (e.g., aspartate aminotransferase (SGOT) or alanine aminotransferase (SGPT) ≥3 × upper limit of normal [ULN])<br>21. Treatment with hyperbaric oxygen within one week prior to study drug.<br>22. Renal insufficiency (e.g., blood urea nitrogen or creatinine ≥2 × ULN) or patients on renal dialysis.<br>23. Use of another investigational drug within 30 days prior to SSHL onset.<br>24. Any other serious medical condition that might interfere with subject evaluation over the 90-day study period, based on the investigator's judgment, e.g. planned surgical intervention or any other condition which might give doubt for non-completing the study e.g. planned holiday, distance between investigational center and place of origin.<br>25. Known hypersensitivity to the drug substance or excipients.<br>26. Blood or blood product infusion within one week prior to study drug.<br>27. Use of any prohibited drug listed in chapter 6.4.1 and 6.4.2 of this study protocol.<br>28. Immunoglobulin infusion 4 weeks prior to study drug.<br>29. Planned treatment using intramuscular injections

	(e.g. vaccinations, vitamin B therapy)<br>30. Conditions associated with an increased risk of hemorrhaging such as major surgery, history of hemorrhagic stroke, intracranial hematoma, subarachnoid hemorrhage, diabetic retinopathy, retinopathy grade 3 or worse, known hemostatic disorders.<br>31. Uremia and renal colic with calculus, cerebrovascular accident, and history of neurosurgery<br>32. History of malignant hypertension and/or diastolic pressure > 105 mmHg<br>33. Acute pericarditis<br>34. Subacute bacterial endocarditis<br>35. Septicemic states with or without evidence of diffuse intravascular coagulation<br>36. History of clinically relevant immediate-type allergy (type I) with respect to e.g. mildew, (house dust) mite, cockroach, hymenoptera venom, dog, cow and honeydew melon<br>37. Hearing loss of infectious or oncology origin
COORDINATING INVESTIGATOR:	
PARTICIPATING COUNTRIES:	Germany, Czech Republic,
STUDY DRUG/TEST MEDICATION:	Vials, each containing an extractable volume of at least 1 ml<br>- solution for injection for s. c.-administration or<br>- concentrate for solution for infusion<br>corresponding to 70 IU / ml ancrod-fibrinogenase or placebo
DURATION OF TREATMENT:	1 week treatment, 3 months follow-up
RECRUITMENT PERIOD:	Actual first patient in: August 2013<br>Recruitment period: 57 months<br>Planned last patient, last treatment: Juli 2018<br>Planned last patient out: October 2018
REFERENCE DRUG:	Placebo (saline solution containing 10 mM sodium phosphates and 0.9 % sodium chloride, pH 6.8)
TREATMENT:	Day 1: Intravenous infusion of 0.167 IU/kg/h with the duration of study drug infusion determined by the patient's baseline fibrinogen level as follows:

	– 2-hour infusion at a baseline fibrinogen concentration of ≥ 180 to ≤360 mg/dL.<br>– 3-hour infusion at a baseline fibrinogen concentration of >360 mg/dL.<br>Days 2, 4, and 6: Subcutaneous injections of 1 IU/kg
SCHEDULE OF EVENTS:	See Schedule of Events, Appendix 17.6 Protocol

1 Abbreviations and Definition of Terms

Give a list of all abbreviations used in the protocol in a table. Examples below, to be adapted (study specific)

AE	Adverse Event
ADR	Adverse Drug Reaction, an AE with a reasonable possibility of a causal relationship with the (investigational) medicinal product
AMG	Arzneimittelgesetz (German Drug Law)
ANCOVA	Analysis of Covariance
ANOVA	Analysis of Variance
aPTT	Activated Partial Thromboplastin Time
AUC	Area Under the Curve
BfArM	Bundesinstitut für Arzneimittel und Medizinprodukte
BUN	Blood Urea Nitrogen
CRF	Case Record Form
CRO	Clinical Research Organization
dB	Decibel
EC	Ethics Committee
ECG	Electrocardiogram
EPC	Endothelial Progenitor Cell
GCP	Good Clinical Practice
GLM	General Linear Model
γ-GT	Gamma-Glutamyltransferase
HAES	Hydroxyethyl Starch
HDL	High Density Lipoprotein
IB	Investigator's Brochure
ICH	International Conference of Harmonization
IMP	Investigational Medicinal Product
INN	International Non-Proprietary Name
IRB /IEC	Institutional Review Board also known as an Independent Ethics Committee
i.v.	Intravenous(ly)
LDH	Lactate Dehydrogenase
LDL	Low Density Lipoprotein
LOCF	Last Observation Carried Forward

MCV	Mean Corpuscular Volume
MedDRA	Medical Dictionary for Regulatory Activities
MWI	Medizinisches Wirtschaftsinstitut GmbH (CRO)
NCR	No Carbon Required
NIH	National Institute of Health
NRS	Numerical Rating Scale
QA	Quality Assurance
QC	Quality Control
PT	Prothrombin Time
PTA	Pure Tone Audiometry
RBC	Red Blood Cell Count
rt-PA	Recombinant Tissue-Type Plasminogen Activator
SAE	Serious Adverse Event
SAP	Statistical Analysis Plan
s.c.	Subcutaneous(ly)
SGOT	Aspartate Aminotransferase
SGPT	Alanine Aminotransferase
SPC	Summary of Product Characteristics
SSHL	Sudden Sensorineural Hearing Loss
SUSAR	Suspected Unexpected Serious Adverse Reaction
TEAE	Treatment Emergent Adverse Event
TT	Thrombin Time
ULN	Upper Limit of Normal
VLDL	Very Low Density Lipoprotein
WBC	White Blood Cell Count
WHO	World Health Organization
X-act	X-act Cologne Clinical Research GmbH (CRO)

2 Background Information

2.1 Introduction

Sudden sensorineural hearing loss (SSHL) is a disease, which severely affects the patient's social and relational life. It is a dysfunction of the inner ear characterized by sudden onset and rapid progression of hearing impairment over a short period of time (hours or days), often occurring in advanced age. Hearing impairment is mostly unilateral with varying severity but potentially leading to complete deafness. It can be accompanied by tinnitus, ear fullness and/or vertigo. SSHL is a sub-entity of the general condition of hearing loss occurring at a much lower incidence than chronic manifestations of hearing loss. According to National Institute of Health (NIH) definition, SSHL is defined as hearing loss of ≥30 dB within 3 contiguous frequencies with sudden onset, developing over up to 72 hours (NIH publication, 2000).

At the time of onset, the etiology of SSHL is mostly unknown. A systematic analysis of 23 research papers performed by Chau et al (2010) revealed that the diagnosis for 71.0% of SSHL patients was idiopathic; only 12.8% of cases were attributed to infectious disease, 4.7% to otologic etiology, 4.2%, to trauma, 2.8% vascular/hematologic, 2.3% to neoplastic, and 2.2% to other causes. SSHL patients thus have to be seen as a diverse group who just happen to suffer from the same symptomatology (Finger and Gostian, 2006). However, a number of experimental and clinical studies indicated that vascular factors significantly contribute to the development of SSHL.

A variety of divergent treatment options have been employed in the treatment of SSHL, although most of them did not gain regulatory approval. Since in most cases the etiology of the condition cannot be determined, a causal therapy is not possible (Suckfüll, 2009). Currently, pentoxifylline, poly (O-2-hydroxyethyl) starch and naftidrofuryl are approved medicinal products for the treatment of sudden hearing loss within a wider indication for use. For all 3 products a comparison of Summary of Product Characteristics (SPCs) among European Union countries showed that the indication pertaining to sudden hearing loss only had been granted in Germany and Austria, whereas this indication was not listed in SPCs of other European countries. The conclusion reached in multiple publications and the current Consensus Guidelines of the German Society of Otorhinolaryngology - Head and Neck Surgery is that all current therapeutic approaches for the treatment of SSHL are considered empiric in nature, and that none of the currently used therapies has shown unequivocal efficacy. Based on a systematic review of randomized clinical studies it has further been concluded that no valid randomized clinical study exist to determine an effective treatment of SSHL (Conlin and Parnes, 2007a, 2007b). Furthermore, the authors stated that systemic steroids cannot be considered the gold standard of treatment, given the severe limitations

of the landmark study of Wilson et al. (1980) suggesting their use. This statement is further supported by a review of the Cochrane Collaboration concluding that the value of steroids in the treatment of idiopathic SSHL remains unclear since the evidence obtained from randomized controlled studies is contradictory in outcome, in part because the studies are based upon a too small number of patients (Wei et al., 2006).

Ancrod (international non-proprietary name, INN) is an enzyme isolated from the venom of the Malayan pit viper *Calloselasma rhodostoma*. It causes a dose-dependent long-lasting anticoagulant effect by decreasing the blood fibrinogen concentration. Therapeutically induced and controlled hypofibrinogenemia diminishes the plasma viscosity and tendency of erythrocytes to aggregate so far that the flow properties of the blood in the microcirculation are crucially improved (Ehrly, 1973). Ancrod had been approved and marketed for more than 15 years in Canada and Europe as treatment for peripheral vascular disease, deep vein thrombosis, central retinal vein thrombosis, and as prophylaxis in subjects at risk for thromboembolism. Due to commercial reasons the owners of the marketing allowance, Knoll/BASF Pharma AG, withdrew ancrod from the market in June 2000.

2.2 Study Rationale

The rationale for the use of ancrod in SSHL is the observation that vascular factors affecting microcirculation and their interaction with the coagulation system significantly contribute to the development of SSHL (Chau et al., 2010; Michel, 1994; Suckfüll et al., 2002). Although genetic predispositions and degenerative processes cannot be ruled out as a determining factor in SSHL, the sudden onset indicates the involvement of an acute ischemic event on the cellular structures of sound perception and neural transmission. Significantly reduced circulating levels of endothelial progenitor cells (Quaranta et al., 2011) associated with activation of lymphocytes (Kassner et al., 2011) and an increased level of circulating adhesion molecules such as ICAM-1 and VCAM-1 (Quaranta et al., 2008) further support the hypothesis of an endothelial dysfunction in patients with SSHL.

The importance of regenerative processes after noxious influences has been shown by Ryals and Rubel (1988). Reactivation of hair cells from a dormant or dedifferentiated stem cell population may be a mechanism explaining rapid recovery after sudden hearing loss, particularly if the treatment starts early enough.

Enhancement of regenerative processes may be attributed to mobilization of circulating endothelial progenitor cells (Zhang et al., 2011) as observed in patients with deep vein thrombosis treated with

batroxobin a defibrinogenating snake venom with similar pharmacodynamic properties as ancrod (Bell 1987).

The hypothesis of improved microcirculation by regenerative processes appears to be confirmed by clinical data obtained from a study with batroxobin (Kubo et al., 1988). In this study, the overall improvement rate in 144 patients with moderate to severe hearing loss was significantly better in patients receiving defibrinogenation therapy than in patients receiving betamethasone. In addition, other symptoms closely related to hearing recovery, such as tinnitus and aural fullness, also showed greater improvement in patients receiving defibrinogenation therapy.

In another clinical study the efficacy of defibrinogenation therapy with batroxobin for SSHL was compared with high-dose steroid therapy in 88 patients who had suffered hearing loss for ≤30 days (Suzuki *e*t al., 2003). The overall results of the 2 treatments were roughly equivalent. However, with regard to patients with initial hearing levels <80 dB, the hearing improvement rate of the batroxobin group was significantly worse than that of the high-dose steroid group, whereas in patients with initial hearing levels ≥80 dB, the hearing outcomes did not differ between the 2 treatment groups. The authors therefore recommend high-dose steroid therapy for patients with moderate hearing loss and defibrinogenation therapy for patients with severe hearing loss.

Batroxobin in combination with low molecular dextran, vasodilators and vitamins was also found effective in 64% of 93 patients with idiopathic sudden deafness (Shiraishi et al., 1993). In this study the interval between onset of hearing loss and start of treatment but not the concentration of fibrinogen was significantly correlated with the degree of hearing recovery, indicating that early onset of defibrinogenation is an important factor for the clinical outcome.

Defibrinogenating agents such as ancrod or batroxobin interfering with impaired microcirculation by either mechanism are expected to support this regenerative process as it was shown by Zhang et al., (2011) in patients with deep vein thrombosis. In these patients mobilization of circulating endothelial progenitor cells contributing to vascular repair has been shown after treatment with batroxobin.

Ancrod had been approved for use and marketed in Canada and Europe for more than 15 years as treatment for peripheral arterial occlusive disease, deep vein thrombosis, and for the prophylaxis of thromboembolism. In addition, clinical research was conducted in patients with intolerance against heparin, as prophylaxis for thromboembolism, and as treatment of acute ischemic stroke. In most of these conditions, the treatment paradigm consisted of several days of intravenous or subcutaneous ancrod administration designed to maintain a target level of hypofibrinogenemia.

Based on clinical results using defibrinogenation in patients with SSHL and the extensive clinical experience with ancrod in various other indications, a short-term, controlled hypofibrinogenemia

appears to be a safe and promising approach for the treatment of SSHL. In this double-blind, randomized, placebo-controlled parallel group proof of concept study in patients diagnosed with unilateral SSHL, ancrod has to confirm that the proposed treatment is both safe and effective if initiated within 7 days after SSHL onset.

2.3 Dosing Rationale

The dosing paradigm for this study is a total ancrod dose of 0.33 IU/kg (or placebo equivalent) administered over 2 hours for subjects with an initial fibrinogen level of ≥ 180 to ≤ 360 mg/dL and a total ancrod dose of 0.50 IU/kg (or placebo equivalent) administered over 3 hours for subjects with an initial fibrinogen level of >360 mg/dL.

The pharmacodynamics, safety and tolerability of this ancrod dosing regimen has been evaluated in 38 healthy subjects who received a total dose of ancrod ranging from 0.125 IU/kg to 0.75 IU/kg over 3 hours (study AN-006, for details see investigator’s brochure). Eighteen subjects received a total dose of 0.5 IU/kg. For those 18, the median fibrinogen level prior to ancrod administration was 235 mg/dL. At the end of the 3-hour infusion, the median fibrinogen level for the group decreased to 162 mg/dL. Median fibrinogen level continued to decrease, to 91 mg/dL at 6 hours after initiation of ancrod infusion and to a nadir of 82 mg/dL at 12 hours. Median fibrinogen level had increased to 88 mg/dL at 16 hours and to 117 mg/dL at 24 hours. Fourteen of the 18 subjects had fibrinogen values over 100 mg/dL at 24 hours; 3 of the remaining 4 subjects had values over 70 mg/dL, and one subject had a level of 68 mg/dL. Thereafter, median values were 147 mg/dL at 48 hours, 163 mg/dL at 72 hours, and 199 mg/dL at 7 days.

Three maintenance doses are planned to be administered subcutaneously following dose recommendations developed for the prevention of deep vein thrombosis after hip surgery (Lowe et al., 1978) and for patients with peripheral arterial circulation disorders (Wolf, 1976). This regimen was shown to be effective and was accompanied with the lowest incidence of bleeding complications (Latallo, 1983).

2.4 Potential Risks and Benefits

2.4.1 Potential Risks

Due to its mode of action, bleeding may occur during ancrod treatment. The direct effect of ancrod on the coagulation cascade and on platelet function may cause microscopic hematuria, bleeding at puncture sites, bruising and, in rare cases, melena, epistaxis, and hematemesis (Latallo, 1983). Many bleeding adverse events are related to bruising and are of limited clinical significance. The

bleeding risk appears to be increased when fibrinogen levels remained below 40 mg/dL for longer periods.

The relatively high incidence of adverse events observed in clinical studies in patients with acute ischemic stroke treated with ancrod may be attributed to advanced age, predisposition, and vulnerability of this patient population and cannot be extrapolated to patients with sudden hearing loss who will be treated with a different, moderate dosing regimen. Further, the incidence of adverse events observed in the direct comparison of defibrinogenation therapy with corticosteroid revealed no minor or major bleeding. Side effects were observed in 84.9% of patients who received defibrinogenation therapy and in 85.9% of patients who received corticosteroid treatment (Kubo et al., 1988).

Local reactions, such as urticaria and exanthema, are rare but may occur after subcutaneous use. Some patients who had received ancrod subcutaneously developed local irritation or itching. These skin reactions have responded to antihistamine therapy. There was no evidence of an allergic reaction in healthy subjects or in patients treated with ancrod repeatedly. Nevertheless, special attention should be paid to patients receiving a second course of ancrod because neutralizing antibodies may occur particularly after long-term subcutaneous administration.

In this placebo-controlled study one third of patients are receiving inactive drug which may be a reason for the lack of efficacy. In such cases where symptoms of the disease are unchanged or aggravated, the patient is allowed to switch on day 8 to corticosteroid treatment, vasodilators, vitamin C, vitamin B12 or antihistamines if deemed necessary at the discretion of the investigator.

2.4.2 Potential Benefits

The therapeutic value of the frequently used oral or intratympanal steroids for the treatment of sudden hearing loss remains unclear (Wei et al., 2006). Other presently used therapies have not been confirmed in state-of-the-art double-blind randomized clinical trials and did therefore not obtain regulatory approval. The existing treatment options for SSHL may thus be comparable to the placebo treatment arm in the planned clinical study.

In contrast, clinical data obtained from a study with batroxobin, a serine protease derived from another snake venom and with similar mode of action as ancrod revealed a good treatment effect in patients with moderate to severe hearing loss. The improvement was significantly better in patients on batroxobin than in patients receiving corticosteroid treatment.

Ancrod’s therapeutic benefit lies in its fibrinogenolytic action which causes reduced plasma fibrinogen concentration, associated with a progressively reduced blood viscosity by 30-40% of the pretreatment levels (Ernst and Dormandy, 1981). The diminished viscosity leads to an

improvement in microcirculation. These rheological changes are sustained and viscosity approaches pretreatment values approximately 10 days after termination of ancrod treatment.

The treatment of SSHL with ancrod represents a novel approach which is expected to exhibit:

- better efficacy than existing therapies
- favorable safety profile
- good patient compliance due to simple dosing regimen

Thus, for each subject, the possibility of a positive therapeutic effect and his or her contribution to the scientific understanding of the properties and actions of ancrod are the only benefits.

3 Design, Objectives and Endpoints

3.1 Study Design

This is a randomized, double-blind, multi-center, placebo-controlled, parallel-group phase II proof-of-concept study comparing ancrod treatment versus placebo in patients with unilateral SSHL. The study will be conducted in approximately 20 sites in Germany, Czech Republic, Serbia and Macedonia. Study treatment and all study assessments will be performed in an outpatient setting. After obtaining informed consent, patients presenting with unilateral sudden hearing loss within 7 days after onset will be screened for eligibility. Patients meeting all in- and exclusion criteria including the screening audiometric assessments will be randomized into 2 cohorts in a ratio of 2:1 between active treatment and placebo according to a randomization code generated prior to start of patient recruitment by the data management/statistics group.

At screening, prior to administration of study drug infusion, standard air and bone pure tone audiometry (PTA) will be measured. In addition, tympanometry, stapedius reflex measurements, otoacustic emissions and speech intelligibility test (specific national test per country to determine the dB value where 50 % of single words will be understood) will be performed. For assessment of vertigo, oculomotor function will be measured using video- or electronystagmography over 30 seconds. Frenzel goggles for the assessment of vertigo are possible as well. The occurrence and the degree of tinnitus will be determined using a numerical rating scale (NRS).

Patients will be screened and enrolled at designated clinical study sites where all test procedures are performed. All screening assessments including randomization and patient enrollment are to be performed within 36 hours. In total, 87 evaluable patients randomized in a 2:1 ratio will be needed to confirm a treatment difference of 15 dB PTA with an α-level of 5% and a statistical power of 90%, assuming a standard deviation of 20%. To compensate for dropouts, a total of

99 patients are planned to be enrolled, 66 patients on ancrod and 33 on placebo. The recruitment period will be approximately 57 months. The study duration for each patient will be 90 days.

Screening measurements (for details see Section 4.1) prior to initiation of study drug administration on Day 1 (Visit 1) will include an assessment of plasma fibrinogen concentration in the local laboratory. Immediately after randomization on Day 1 (Visit 1), patients will receive the initial infusion of investigational product. The initial dose of ancrod will be adjusted according to the screening fibrinogen concentration by using different infusion durations at a constant infusion rate (see Section 7.1).

On the following day (Day 2, Visit 2), a venous blood sample will be taken for coagulation assays (fibrinogen, thrombin time [TT], prothrombin time [PT], activated partial thromboplastin time [aPTT]) to be performed in the selected responsible laboratory (local or central) of the site within 36 hours. The investigator must announce the taking of the blood sample for fibrinogen per fax to Nordmark (Pharmaceutical Development, unblinded personnel). Thereafter, on Day 2, a weight-adjusted ancrod dose of 1 IU/kg will be administered subcutaneously. Nordmark Pharmaceutical Development has to be informed immediately in writing (fax) from the responsible laboratory or designee on the result of the coagulation assays. The result of the coagulation assay may in principle not be communicated from the laboratory to the investigator or any other blinded study personnel. If the fibrinogen concentration on Day 2 is lower than 50 mg/dl the investigator will be informed by Nordmark Pharmaceutical Development that this patient may not receive further injections of ancrod on Day 4 and Day 6. This deviation has to be documented in the dosing worksheet. All other study related activities will be performed as planned. If the fibrinogen concentration on Day 2 is ≥ 50 mg/dl Nordmark Pharmaceutical Development will inform the investigator that the patient may receive further subcutaneous injections of 1 IU/kg ancrod on Day 4 and Day 6 (Visits 3 and 4) (Figure 1). Regardless of the result, Nordmark PharmaceuticalDevelopment may not communicate any values to the investigator or any other blinded study personnel.

On Day 8 (Visit 5) the treatment effect will be evaluated by means of audiometry and speech recognition, and the presence and intensity of tinnitus and vertigo will be determined. In addition, a blood sample will be taken for coagulation assays and biomarkers will be measured. The result of the coagulation assay may in principle not be communicated from the laboratory to the investigator or any other blinded study personnel. If, on Day 8 (Visit 5), no improvement of clinical signs and symptoms compared to the baseline value has been achieved, the patient will be allowed to switch to corticosteroid treatment, vasodilators, vitamin C, vitamin B12 or antihistamines if deemed necessary at the discretion of the investigator.

During the follow-up visits on Day 30 and Day 90 (Visits 6 and 7) the treatment effect will again be determined by audiometric evaluation and tinnitus intensity to monitor for signs of relapse in the affected ear. Comparison of this audiogram to the audiogram at enrollment will determine the stability of the hearing improvement after the end of treatment.

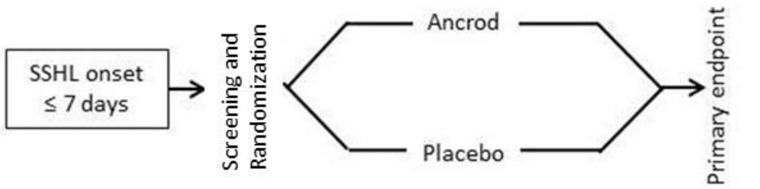

Day	1	2	3	4	5	6	7	8	30	90
Visit	1	2		3		4		5	6	7
Dosing	i.v.	s.c.		s.c.		s.c.				
Audiometry	X							X	X	X

Figure 1: Study Flow Chart

i.v. =intravenous, s.c. =subcutaneous, SSHL = sudden sensorineural hearing loss.

3.2 Objectives and Endpoints

The primary objective of this study is to evaluate the efficacy of ancrod as primary treatment of SSHL compared to placebo in patients with unilateral sudden sensorineural hearing loss as determined by audiometry.

The secondary objectives of this study are to:

- Evaluate the relationship between biomarkers and clinical efficacy.
- Investigate the safety and tolerability of ancrod.

3.2.1 Primary Outcome Measure

The primary outcome measure of the study will be the change in PTA in the affected ear from screening (Day 1; Visit 1) until Day 8 (Visit 5). The PTA will be calculated as the arithmetic mean of air conduction thresholds at the affected consecutive frequencies in the frequency range of 0.125 to 8 kHz. A non-affected frequency within two affected frequencies will be included.

3.2.2 Secondary Outcome Measures

Secondary outcome measures will include:

- Change in word recognition score from screening to Day 8, Day 30 and Day 90.

- Change of audiogram in the affected ear from screening to Day 30 and Day 90.
- Change in fibrinogen concentration from screening to Day 2 and Day 8.
- Change in biomarkers from screening to Day 8.
- Patient assessment of change in hearing impairment.
- Physician assessment of change in hearing impairment.
- Change in tinnitus severity.

4 Schedule of Events and Study Duration

This section provides a detailed description of the study procedures to be performed at each visit. For an overview on study procedures please refer to the study schedule in Appendix 17.6.

4.1 Screening (Day 1, Visit 1)

All screening assessments including randomization and patient enrollment are to be performed within 36 hours. Informed consent is to be obtained from each patient prior to any study-related procedures. After obtaining informed consent, the following assessments will be performed prior to initiation of study drug infusion on Day 1 (Visit 1):

- Review of inclusion/exclusion criteria.
- Review of demographic and baseline characteristics.
- General physical and otolaryngological examination including otoacustic emissions, tympanometry, stapedius reflex measurements
- Vital signs.
- Review of medical history as well as previous medication (all medication taken within the last 30 days prior to Visit 1) and concomitant medication.
- Pregnancy test.
- Audiometric evaluation (PTA and speech intelligibility test inclusive word recognition).
- Occurrence and severity of tinnitus
- Vertigo evaluated using video- or electronystagmography or Frenzel goggles.
- Patient assessment of change in hearing impairment assessed using NRS
- Physician assessment of change in hearing impairment assessed using NRS
- Biomarkers (see Section 8.2.1).

- Blood sample for neutralizing anti-ancrod-antibodies
- Safety laboratory assessments including coagulation, hematology, clinical chemistry, and urinalysis (for details see Section 8.2.2).

Immediately after randomization, patients will receive the initial administration of study drug (active or placebo) as intravenous infusion on the first day of treatment. The initial dose of study drug will be adjusted according to the screening fibrinogen concentration by using different infusion durations at a constant infusion rate (see Section 7.1).

All screening assessments including randomization and patient enrollment are to be completed within 36 hours.

AEs will be recorded starting from initiation of study drug infusion.

4.2 Visits of the Double-Blind Treatment Phase (Days 2, 4, 6 and 8)

Day 2 (Visit 2)

On the following day (Day 2, visit 2), a venous blood sample will be drawn for the determination of fibrinogen, TT, PT, and aPTT. This assay will be performed within 36 hours in the selected laboratory (local or central) of the site for reason of safety. The result of this coagulation analysis will be immediately sent to Nordmark Pharmaceutical Development. If the fibrinogen concentration on Day 2 is lower than 50 mg/dl the investigator will be informed by Nordmark Pharmaceutical Development that this patient may not receive further injections of ancrod on Day 4 and Day 6. This deviation has to be documented in the dosing worksheet. All other study related activities will be performed as planned. If the fibrinogen concentration on Day 2 is ≥ 50 mg/dl, Nordmark Pharmaceutical Development will inform the investigator that the patient may receive further weight adjusted subcutaneous injections of study drug at a dose of 1 IU/kg ancrod-fibrinogenase or placebo. Regardless of the result, Nordmark Pharmaceutical Development may not communicate any values to the investigators.

AEs and concomitant medication will be recorded.

Day 4 (Visit 3) and Day 6 (Visit 4)

The following assessments and treatments will be done on Days 4 and 6 of the double-blind treatment phase:

- Review of concomitant medication.
- Weight adjusted subcutaneous administration of study drug (1 IU/kg ancrod-fibrinogenase or placebo).
- Recording of AEs.

Treatments and assessments of day 4 and day 6 may be shifted to the following day, however, it must be ascertained that between the two visits there is one day without treatment.

Day 8 (Visit 5)

Day 8 will be the last day of the double-blind treatment phase. The assessments of day 8 may be shifted to the previous or the following day, however, visit 4 and visit 5 are to be performed on different days.

On day 8 no study drug will be administered and the following assessments will be performed:

- General physical and otolaryngological examination including otoacustic emissions, tympanometry, stapedius reflex measurements.
- Vital signs.
- Review of concomitant medication.
- Audiometric evaluation (PTA and word recognition).
- Presence and severity of tinnitus.
- Vertigo assessed by video- or electronystagmography or Frenzel goggles.
- Patient assessment of change in hearing impairment.
- Physician assessment of change in hearing impairment.
- Safety laboratory assessments including coagulation, hematology, clinical chemistry, and urinalysis.
- Biomarkers.
- Blood sample for neutralizing anti-ancrod-antibodies
- Recording of AEs.

4.3 Follow-up Visits at Day 30 and Day 90 (± one week)

The following assessments will be done at the follow-up visits on Day 30 and Day 90:

- Review of concomitant medication.
- Audiometric evaluation (PTA and word recognition).
- Presence and severity of tinnitus.
- Patient assessment of change in hearing impairment.
- Physician assessment of change in hearing impairment.
- Recording of AEs.

– Blood sample for neutralizing anti-ancrod-antibodies (only on Day 30)

4.4 Final Study Visit at Day 90 (± one week)

The follow-up visit on Day 90 will be the final study visit.

4.5 Early Termination Visit

If a patient prematurely terminates the study, a complete final examination as detailed in Section 4.2 is to be performed at the time of withdrawal. Furthermore the blood sample for determination of the neutralizing anti-ancrod antibody should be taken on day 30 (+/- one week) if possible.

4.6 Unscheduled Visit

Not applicable.

4.7 Duration of the study

This study was initiated (first patient in) in August 2013. The recruitment period is planned to be approximately 57 months. The study duration for each patient will be 90 days. The overall duration of the study is planned to be 57 + 3 = 60 months, with the last patient out in August 2018.

<u>5 Patients</u>

5.1 Study Population

This study will include patients presenting with unilateral idiopathic sudden hearing loss. The criteria for defining the SSHL diagnosis, hearing loss severity, and hearing improvement will be based on pure tone air and bone conduction thresholds collected during standardized audiometric tests including standard air and bone conduction PTA, tympanometry, stapedius reflex measurements, otoacustic emissions and speech intelligibility test (specific national test per country to determine the dB value where 50 % of single words will be understood).

It is planned to randomize a total of 99 patients to either ancrod or placebo in a 2:1 ratio (i.e. 66 patients receiving ancrod and 33 patients receiving placebo. For details on sample size determination see Section 10.1.

5.1.1 Patients and Characteristics

Patients must meet all of the inclusion and exclusion criteria as outlined in Section 5.1.2 and Section 5.1.3, to be eligible for randomization into the double-blind treatment period at the screening visit (Visit 1).

5.1.2 Inclusion Criteria

The criteria for defining the SSHL diagnosis are based on pure tone air and bone conduction thresholds collected during standardized audiometric tests. Patients must meet all of the following inclusion criteria in order to be eligible for this study:

1. Unilateral idiopathic sudden sensorineural hearing loss ≥30 dB in at least 2 consecutive frequencies in the affected ear or ≥20 dB in 3 consecutive frequencies based upon evaluation of 8 frequencies, 0.125, 0.25, 0.5, 1, 2, 4, 6, and 8 kHz, compared to the contralateral ear.
2. Symmetric hearing prior to onset of SSHL, according to patient's recollection.
3. Patients with hearing loss not greater than 90dB at 0.125, 0.25, 0.5, 1, 2, 4, 6, and 8 kHz.
4. Enrollment has to be accomplished within 7 days after SSHL onset.
5. Male or female aged ≥18 to ≤70 years.
6. Women of childbearing potential who are sexually active with opposite partners have to perform adequate contraception with a combination of a highly effective method of birth control and additional barrier contraception.
   [Highly effective method of birth control is defined as those, alone or in combination, that result in a low failure rate (i.e. less than 1% per year) when used consistently and correctly for the entire study duration: combined (oestrogen and gestagen) oral contraceptives, hormone implants, hormone injectables, or hormone containing intrauterine device that needed to be in place for a period of at least 2 months prior to screening. Additional barrier contraception (at least the following methods are allowed: condom of the male, diaphragm with spermicide, portio cap with spermicide) has to be used for the duration of the trial, defined as from the time of screening to at least 10 days after Day 6 (Visit 4) of the double-blind treatment phase. A single barrier method is not acceptable. Women of non-childbearing potential can be included if surgically sterile (documented complete hysterectomy or bi-tubal ligations) or post-menopausal >1 year.]
7. Men of reproductive potential must use condoms. In addition, the female partner should also be on a safe hormonal contraception (e.g. combined oral contraceptives, hormone implants, hormone injectables or hormone containing intrauterine device) or use a barrier contraception (e.g. intrauterine device, diaphragm or portio cap with spermicide), if she is of childbearing potential.
8. Ability to understand and to follow the study protocol.

5.1.3 Exclusion Criteria

The presence of any of the following will exclude a patient from study enrolment:

1. Pregnant or breast-feeding female.
2. positive pregnancy test before receiving study drug
3. Body weight > 140 kg
4. Bilateral SSHL.
5. Incomplete recovery after previous SSHL, when symmetric hearing was not reached again, according to patient's recollection.
6. Due to history of physical efforts suspected perilymph fistula or membrane rupture.
7. Previously existing, known retrocochlear hearing loss.
8. Any history of any ear operation or local inflammatory disease in the past one year.
9. History of blunt or penetrating ear trauma, head trauma, barotrauma, or acoustic trauma immediately preceding SSHL.
10. History of Menière's disease, autoimmune hearing loss, radiation-induced hearing loss, endolymphatic hydrops.
11. Any pre-treatment within the preceding 30 days prior to screening, ongoing treatment or planned treatment of SSHL-related hearing loss.
12. History of chronic inflammatory diseases or autoimmune diseases e.g. rheumatic disease, including rheumatoid arthritis, scleroderma, lupus erythematosus, polymyalgia rheumatica, polyarteritis nodosa, temporal/giant cell arteritis, Sjögren's syndrome, or ulcerative colitis and Crohn's disease.
13. History of unstable angina, coronary artery stenting or bypass grafting within three months of enrolment, transient ischemic attacks or stroke within four weeks of enrolment.
14. Prior chemotherapy or treatment with immunosuppressant drugs (azathioprine, chlorambucil, cyclophosphamide, or other alkylating agents), cyclosporine, etanercept, infliximab, interferon or any therapy with drugs known as ototoxic (e.g. aminoglycosides, cisplatin, loop diuretics, quinine etc.) in the past 6 months.
15. Gastrointestinal pathology: lesions liable to bleed such as active peptic ulcer disease, inflammatory bowel disease, history of gastrointestinal bleeding, or recent hemorrhage at any anatomical site requiring medical intervention.
16. Screening plasma fibrinogen level of <180 mg/dL.

17. Known disorder of platelet function or coagulation abnormality.
18. Platelet count of <100,000/mm^3.
19. Severe liver disease.
20. Hepatic failure (e.g., aspartate aminotransferase (SGOT) or alanine aminotransferase (SGPT) ≥3 × upper limit of normal [ULN]).
21. Treatment with hyperbaric oxygen within one week prior to study drug.
22. Renal insufficiency (e.g., blood urea nitrogen or creatinine ≥2 × ULN) or patients on renal dialysis.
23. Use of another investigational drug within 30 days prior to SSHL onset.
24. Any other serious medical condition that might interfere with subject evaluation over the 90-day study period, based on the investigator’s judgment, e.g. planned surgical intervention or any other condition which might give doubt for non-completing the study e.g. planned holiday, distance between investigational center and place of origin.
25. Known hypersensitivity to the drug substance or excipients
26. Blood or blood product infusion within one week prior to study drug.
27. Use of any prohibited drug listed in chapter 6.4.1 and 6.4.2 of this study protocol
28. Immunoglobulin infusion 4 weeks prior to study drug
29. Planned treatment using intramuscular injections (e.g. vaccinations, vitamin B therapy)
30. Conditions associated with an increased risk of hemorrhaging such as major surgery, history of hemorrhagic stroke, intracranial hematoma, subarachnoid hemorrhage, diabetic retinopathy, retinopathy grade 3 or worse, known hemostatic disorders.
31. Uremia and renal colic with calculus, cerebrovascular accident, and history of neurosurgery
32. History of malignant hypertension and/or diastolic pressure > 105 mmHg
33. Acute pericarditis
34. Subacute bacterial endocarditis
35. Septicemic states with or without evidence of diffuse intravascular coagulation
36. History of clinically relevant immediate-type allergy (type I) with respect to e.g. mildew, (house dust) mite, cockroach, hymenoptera venom, dog, cow and honeydew melon
37. Hearing loss of infectious or oncology origin

5.1.4 Withdrawal of Patients

Patients may be withdrawn from further participation in the study for any the following reasons:

- At their own request without giving reasons (including drop-outs and patients lost to follow-up).
- At the discretion of the investigator, at any time during the study period also in case the investigator considers the patient is not treated appropriately during the course of the study.
- Development of AE, laboratory abnormality, inter-current illness, or other medical condition or situation such that continued participation in the study would not be in the best interest of the patient.
- Meeting any exclusion criteria (either newly developed or not previously recognized).

IMP administration should be interrupted in case of worsening of the health status; medical conditions to be taken into consideration may include but not be limited to pain, gastrointestinal disorder, skin reactions, bleeding of the skin, excretion of blood from intestine or urinary tract or profuse bruises.

If a patient is prematurely withdrawn, a complete final examination as detailed in Section 4.2 is to be performed at the time of withdrawal and the reason for withdrawal has to be documented. Withdrawn patients will not be replaced.

5.1.5 Patient Log

A patient screening log will be used to document the characteristics of all patients initially considered for inclusion into the study to ensure that the included sample does not differ significantly from the available patient population. The reason for exclusion should be given for all patients who are screened but not included.

For patients who are included, a separate patient log / identification list will be used to document name, birth date, gender and date of informed consent.

An example of the patient log/Identification list and the patient screening and enrolment log is provided in Appendix 17.1 and 17.2.

6 Study Drug and Related Procedures

Each approved study site will receive the treatment kits from Nordmark Arzneimittel GmbH & Co. KG. The kits will be shipped refrigerated (+ 2 °C to + 8 °C).

One treatment kit contains 2 cardboard boxes. One cardboard box contains 6 clear glass vials, the second cardboard box contains 3 clear glass vials. Each Vial contains an extractable volume of at least 1 ml
- solution for injection for s. c.-administration or
- concentrate for solution for infusion
corresponding to 70 IU / ml ancrod-fibrinogenase or placebo.

Upon arrival of the shipment at the study site:

- Inform Nordmark Arzneimittel GmbH & Co. KG immediately about the receipt of the treatment kits by fax or e-mail.
- Store the treatment kits immediately in a refrigerator according to the protocol. All treatment kits must be kept refrigerated until use and between all treatments at + 2 °C to + 8 °C but may not be frozen.
- Proceed with the thermometer and / or temperature logger according to the corresponding standard operating procedure document provided by Nordmark Arzneimittel GmbH & Co. KG.

  In case of (potential) temperature excursions:

  - the enclosed temperature logger should be read out immediately
  - the potential impact on the quality of the study medication should be evaluated by Nordmark Arzneimittel GmbH & Co. KG.

  All procedures to be fulfilled in case of potential temperature excursions by the clinical center, including e.g. temperature logger handling, are described in the corresponding standard operating procedure document provided by Nordmark Arzneimittel GmbH & Co. KG.

All used and unused vials of study medication must be maintained until the conclusion of the study. Diluted infusion solutions may be discarded after use. Unused vials should remain refrigerated (+ 2 °C to + 8 °C).

6.1 Study Product

6.1.1 Formulation, Packaging, and Labelling

Each vial of the study medication contains an extractable volume of at least 1 ml
- solution for subcutaneous injection or
- concentrate for solution for infusion.

The clear, colorless aqueous solution contains 70 IU / ml Ancrod-Fibrinogenase or placebo (see point 6.2). It will be distributed as clear 2-mL glass vials, closed with a rubber stopper, which in turn is covered with a red flip cap.

Table 1: Active Formulation

**Ingredient**	**Unit/weight**
Active substance	70 IU/mL ancrod-fibrinogenase (63 – 77 IU/mL)
Buffer	10 mM sodium phosphates, 0.9% sodium chloride
pH	6.5 – 7.1

One treatment kit contains 2 cardboard boxes. One cardboard box contains 6 clear glass vials, the second cardboard box contains 3 clear glass vials.

- 1 of these 9 vials is intended to be used for intravenous administration on Day 1 as described under point 6.1.2.
- 6 of these 9 vials are intended to be used for subcutaneous administration on Day 2, 4 and 6, corresponding to 2 vials for each of these treatment days, as described under point 6.1.2.
- 2 of these 9 vials are intended for backup reasons.

The vials and the treatment kits are labeled in compliance with legal and regulatory requirements of the respective country.

6.1.2 Preparation, Administration, Storage, and Dosage of Study Product

Administration on Day 1

The solution prepared for intravenous administration on Day 1 will contain a concentration of 1.4 IU/ml of ancrod-fibrinogenase or placebo. Therefore 1,0 mL concentrate for solution for infusion (containing 70 IU/mL ancrod-fibrinogenase or placebo) is to be taken out of one vial by a 1 ml syringe and transferred through a three-way-stopcock into a 50 mL perfusor syringe which will subsequently be filled with saline to achieve a final volume of 50 ml.

Solutions for infusions shall be freshly prepared and must be administered within one hour after preparation.

The infusion on Day 1 is to be administered at a rate of 0.167 IU/kg/hr for a maximum of 3 hours. A single 50-mL syringe will thus be sufficient for a patient weighing up to 140 kg.

The individual infusion rate is calculated according to the formula:

$$\textit{Infusion Rate} = \frac{\textit{Dose x Body Weight}}{\textit{Concentration}}$$

with the following dimensions:	*Infusion rate:*	*ml/h*
	Dose:	*IU/kg/h*
	Body weight:	*kg*
	Concentration:	*IU/ml*

Example for a 75 kg patient:

$$\textit{Infusion Rate} = \frac{0.167\ x75}{1.4} = 8.95\ ml/h$$

Since the ancrod dose and concentration of the infusion solution are fixed in this study, the formula can be simplified as follows:

Infusion Rate (ml/h) = 0.1193 x Body weight (kg)

The calculated infusion rate has to be documented in the dosing worksheet (Appendix 17.4).

The maximum single dose applied intravenously is 70 IE.

It would apply for a 140 kg patient having a screening fibrinogen concentration of > 360 mg/dL and receiving a 3-hour infusion of 0.167 IU/kg/h ancrod-fibrinogenase.

Administration on Days 2, 4, and 6

For the subcutaneous injections on Days 2, 4, and 6 the concentrate solution containing 70 IU/mL ancrod-fibrinogenase or placebo will be used. The contents of one or two vials will be drawn into a 2 ml syringe, the volume adjusted according to the patient's weight. Injections shall be freshly prepared and must be administered within one hour after preparation.

The volume to be administered is calculated as follows:

Injection volume (ml) = 0.0143 x Body weight (kg)

The calculated injection volume has to be documented in the dosing worksheet (Appendix 17.4).

The maximum single dose applied subcutaneously is 140 IE.

It would apply for a patient on treatment day 2, and optionally on day 4 and day 6.

Storage

The treatment kits must be stored refrigerated at + 2 °C to + 8 ºC. After vial opening the vial the solution should be used immediately either as a concentrate for solution for infusion or as a solution for s. c. injection.

6.2 Reference Product

Table 2: Placebo formulation

Ingredient	Unit/weight
Buffer (aqueous)	10 mM sodium phosphates, 0.9 % sodium chloride
pH	6.5 – 7.1

6.3 Concomitant Medication

Paracetamol may be taken for pain relief.

6.4 Precautionary and Prohibited Medications and Procedures

6.4.1 Prohibited Medications and Procedures

Subjects who received thrombolytic agents (e.g. rt-PA, urokinase, streptokinase), anticoagulants (e.g. warfarin, phenprocoumon, heparin, low molecular weight heparin, factor Xa inhibitors such as rivaroxaban, apixaban, dabigatranetexilat), antiplatelet drugs (e.g. pentoxifylline, naftidrofuryl, prostacyclin, prostaglandin, nicotinic acid, acetylsalicylic acid, abciximab) within 72 hours or systemic available corticosteroids (tablets, capsules, injections or infusions) within 30 days prior to screening are not to be enrolled in the study. A previously existing topic corticosteroid therapy with ointments, inhalativa or nasal sprays can be discontinued, reduced or continued in a stable dose during the treatment phase.

Thrombolytic agents should not be given within 5 days after initiating study drug. Anticoagulants should not be given within 72 hours after initiating study drug infusion.

In addition, the following procedures and medications are not allowed throughout the study with the exception of the rescue medication (see section 6.5):

- Hyperbaric oxygen
- Plasmapheresis
- Anticoagulants (e.g. warfarin, phenprocoumon, heparin, low molecular weight heparin, factor Xa inhibitors such as rivaroxaban, apixaban, dabigatranetexilat)
- Antiplatelet drugs (e.g. pentoxifylline, naftidrofuryl, prostacyclin, prostaglandin, nicotinic acid, acetylsalicylic acid)

- Thrombolytic agents (e.g. rt-PA, urokinase, streptokinase)
- Hydroxyethyl starch (HAES), low-molecular-weight dextran, local anesthetics (lidocain, procain)
- Blood or blood products
- Systemic available corticosteroids and start of therapy with topic corticosteroids
- NSAIDs

6.4.2 Precautionary Medications and Procedures

Ancrod should not be used in patients receiving drugs that block the reticuloendothelial system (e.g., dextran) or interfere with the fibrinolytic system such as ε-aminocaproic acid or tranexamic acid.

6.4.3 Prophylactic Medications and Procedures

Not applicable.

6.5 Rescue Medications

Corticosteroids, vasodilators, vitamin C, vitamin B12 or antihistamines are allowed if deemed necessary at the discretion of the investigator, if, on day 8, no improvement or worsening of symptoms has been observed.

6.6 Randomization and Blinding Procedures

The present study is carried out in a double-blind, placebo-controlled study design, i.e. neither patient nor investigator have been informed whether the test substance or a placebo have been administered. The randomization is done in a 2:1 ratio verum to placebo.

The randomization was carried out by Medizinisches Wirtschaftsinstitut GmbH (MWI), München, using commercial software (RANCODE Professional, Version 3.6). A randomization list was generated in ascending order with the block randomization method in the above-mentioned ratio between verum and placebo. Further characteristics of the block randomization carried out are not mentioned until being cited in the Biometric Report or the Clinical Study Report at the end of the study once it has been unblinded. The randomization list is kept by MWI.

All investigators have to follow and ensure the trial's randomization procedure. To this end, each center has to treat the patients meeting the inclusion criteria with the investigational products provided in ascending order, taking care of the chronological order of the patients' inclusion. The

first patient included in a study center receives the investigational product with the lowest number given to the center. The second patient in a center receives the product one number higher.

The inclusion has to be exactly documented stating the precise date and time. Furthermore, the investigators have to document immediately every premature unblinding of a patient (e.g. unblinding because of a SUSAR or accidental unblinding) including prompt information of the monitor and stating the reasons to the Sponsor.

6.6.1 Maintenance of Study Treatment Randomization Codes

MWI prepares emergency envelopes corresponding to the labeled test substances, so that an individual unblinding will be possible if needed. The numbered emergency envelopes have to be dispatched together with the identically numbered test substances.

The randomization list and the allocation to treatment groups will not be known to the investigator, sponsor, or any other person involved in the conduct of the study except staff involved in IMP (Investigational Medicinal Product) production, until completion of the study, except in the case of an emergency. Each investigator will be provided with emergency code break envelopes containing the information to which treatment an individual patient number was allocated. This information will only be eligible after opening the envelope. An envelope may only be opened in the case of emergency when it is necessary for medical reasons to know which study treatment a specific patient has received. The investigator has to document the reason for breaking the code. The signed and dated letter will be archived in the investigator’s file.

Furthermore, the drug safety officer of the sponsor will be provided with emergency code break envelopes containing the information to which treatment an individual patient number was allocated. This information will only be eligible after opening the envelope. An envelope may only be opened in the case of a suspected unexpected serious adverse reaction (SUSAR) to fulfill the reporting obligations to the authorities and ethics committee according to chapter 7.11 of European Commissions guidance (CT-3). The drug safety officer will not inform other sponsor’s staff or investigator about the unblinded information unless unblinded information is judged necessary for safety reasons. The drug safety officer has to document the reason for breaking the code. The signed and dated letter will be archived in a closed safe until the study is completed.

The entire study is not unblinded until its completion with closure of the data base. Upon completion of the study, all distributed emergency envelopes will be collected by the monitor, each envelope being examined for having been opened.

6.6.2 Additional Measures to Minimize/Avoid Bias

Not applicable

6.7 Drug Accountability

The investigator will record and acknowledge receipt of all shipments of study drug. The study drug must be kept in a locked area with restricted access, stored and handled in accordance with the manufacturer's instructions. The investigator is responsible for maintaining documentation showing the amount of study drug provided to the investigational site and dispensed to each study patients (see Appendix 17.4). Discrepancies in study drug accountability must be explained and documented. An inventory of study drugs will be maintained. The monitor is responsible for verifying the investigator's documentation on receipt, use and return of study drug. The monitor will prepare a final report of the accountability of the study drug for filing in the investigator's file. Unused medication will be returned to the sponsor for destruction.

7 Therapy

7.1 Treatment Schedule

Patients will receive study mediation on Days 1, 2, 4, and 6 of the study as follows:

Administration and Dosage on Day 1

On Day 1 (Visit 1) study medication will be administered as intravenous infusion. The duration of the infusion will be adjusted according to the screening fibrinogen concentration as assessed prior to the infusion in the local laboratory:

- 2-hour infusion of 0.167 IU/kg/h ancrod-fibrinogenase or placebo at a screening fibrinogen concentration of ≥ 180 to ≤ 360 mg/dL, corresponding to a total dose of 0.33 IU/kg.
- 3-hour infusion of 0.167 IU/kg/h ancrod-fibrinogenase or placebo at a screening fibrinogen concentration of > 360 mg/dL, corresponding to a total dose of 0.50 IU/kg.

The maximum single dose applied intravenously is 70 IE.

It would apply for a 140 kg patient having a screening fibrinogen concentration of > 360 mg/dL and receiving a 3-hour infusion of 0.167 IU/kg/h ancrod-fibrinogenase.

Study drug will be administered through a dedicated intravenous line using an infusion pump. Patients with a baseline fibrinogen level of <180 mg/dL must not receive the study drug.

If the infusion has been interrupted for any reason (e.g., infiltration), it should be resumed at the same infusion rate as before, i.e., the infusion rate may not be increased in an effort to administer the full dose within the original time frame.

Any deviation from the planned infusion duration has to be documented.

Administration and Dosage on Days 2, 4, and 6

On Day 2, patients will receive a maintenance subcutaneous injection of investigational drug or placebo at a fixed dose of 1 IU/kg using undiluted solution. On Day 4 and Day 6 patients will receive maintenance subcutaneous injections only if the plasma fibrinogen concentration, measured on Day 2 is ≥ 50 mg/dl. If the fibrinogen concentration on Day 2 is lower than 50 mg/dl this patient may not receive further injections of ancrod.

The maximum single dose applied subcutaneously is 140 IE.

It would apply for a 140 kg patient on treatment day 2, and optionally on day 4 and day 6.

7.2 Compliance

Not applicable.

7.3 Continuation of Therapy after the End of the Study

If no improvement in efficacy measurements compared to the baseline value is achieved by the end of the double-blind treatment phase (Day 8; Visit 5), the patient will be allowed to be treated according to accepted Standard of Care.

8 Assessments, Materials and Methods

All assessments will be performed in an outpatient setting.

8.1 Clinical Assessments

8.1.1 Efficacy Assessments

Clinical efficacy assessments will include:

- Audiometric evaluation
- Occurrence and severity of tinnitus
- Video- or electronystagmography or Frenzel goggles
- Patient assessment of change in hearing impairment

– Physician assessment of change in hearing impairment

Audiometric evaluation

Audiometric tests will include standard air and bone conduction PTA.

Speech intelligibility test: Specific national test per country to determine the dB value where 50 % of single words will be understood.

Tinnitus

The occurrence and the degree of problems that tinnitus will be quantified using a numerical rating scale.

Vertigo

For assessment of vertigo, oculomotor function will be evaluated using videonystagmography or electronystagmography over 30 seconds. Frencl's goggles for assessment of vertigo are possible as well.

Patient Assessment of Change

At Visits 1, 5, 6, and 7 (Days 1, 8, 30, 90, or early withdrawal visit), the patient will assess his/her change in hearing ability using a numerical rating scale.

Physician Assessment of Change

At Visits 1, 5, 6, and 7 (Days 1, 8, 30, 90, or early withdrawal visit), the physician will assess the patient's change in hearing ability using a numerical rating scale.

8.1.2 Safety Assessments

Clinical safety assessments will include:

– AE reporting.

– Changes from screening (Day 1; Visit 1) to Day 8 in general physical and otolaryngological examination findings.

– Changes from screening (Day 1; Visit 1) to Day 8 in vital signs.

Adverse Event Reporting

Incidences and types of AEs will be recorded in the CRF through Day 90 after initiation of study drug as described in Section 9.1.

General Physical and Otolaryngological Examination

The following assessments will be performed on Day 1 (Visit 1) and Day 8 (Visit 5):

Physical examination

The examination should include at least the following:

- General appearance (including lymphatic nodes)
- HEENT (head, eyes, ears, nose and throat)
- Cardiovascular
- Respiratory
- Gastrointestinal/Abdomen
- Musculoskeletal
- Skin/Dermatological
- Neurologic
- Other examinations

The physical examination is appropriate to record typical and possible adverse events (e.g. dermatorrhagia, bronchoconstriction). The patient has to be informed, how to detect clinical signs of adverse events and to contact the investigator in case of upcoming events.

Otolaryngological examination

- Otoacustic emissions
- Tympanometry
- Stapedius reflex measurement
- Ear microscopy

Vital Signs

- Blood pressure
- Pulse rate
- Body weight
- Height (only at screening)

8.2 Laboratory Assessments

8.2.1 Efficacy Assessments

Inflammatory and cellular markers will be determined as efficacy laboratory assessments in a central laboratory. Blood samples for inflammatory and cellular markers will be drawn prior to study drug infusion (baseline value at screening) and 8 days (Visit 5) after initiation of study drug infusion.

The following parameters will be analysed as surrogate parameters for efficacy:

- Inflammatory markers:, interleukin-6, TNF-α
- Molecular markers of leucocyte activation: CD38+, CD40+ cells

Inflammatory and cellular markers will be correlated with clinical effects.

8.2.2 Safety Assessments

Laboratory safety assessments will be performed on the changes in the following laboratory parameters.

Coagulation

Blood samples for coagulation (Fibrinogen according to Clauss, TT, PT and aPTT) will be drawn prior to study drug infusion (baseline value at screening), two days (Visit 2) and 8 days (Visit 5) after initiation of study drug. Coagulation parameters during screening are used for the determination of the duration of study drug infusion and will be assessed in the local laboratory. The following measurements will be performed in the selected laboratory (local or central) of the site.

Hematology, Clinical Chemistry, Urinalysis, and Pregnancy Test

Venous blood samples will be drawn for the assessment of safety laboratory parameters and biomarkers on Day 1 (Visit 1) and Day 8 (Visit 5). The individual parameters will be assessed in the responsible laboratory as follows:

Hematology

Venous blood samples will be drawn for the assessment of the following parameters on Day 1 (Visit 1) and Day 8 (Visit 5): erythrocytes, hematocrit, MCV, leucocytes, erythrocyte sedimentation rate, hemoglobin, thrombocytes.

Clinical Chemistry

Venous blood samples will be drawn for the assessment of the following parameters on Day 1 (Visit 1) and Day 8 (Visit 5): creatinine, aspartate aminotransferase (SGOT; AST), alanine aminotransferase (SGPT; ALT), sodium, potassium, chloride, glucose, urea, uric acid, phosphorus, total bilirubin, total protein, albumin, lactate dehydrogenase (LDH), gamma-glutamyltransferase (γ-GT), alkaline phosphatase, creatinine phosphokinase, triglyceride, cholesterol, high density lipoprotein (HDL), low density lipoprotein (LDL), and very low density lipoprotein (VLDL).

Urinalysis

At screening and on day 8: Hemoglobin or blood in urine, pH, specific gravity, protein, glucose, ketones, bilirubin, and leucocytes.

Pregnancy test

A pregnancy test will be performed in premenopausal women who have not been surgically sterilized. The test will be performed on the first available urine sample or in serum. Study drug may not be started before obtaining pregnancy test results.

8.2.3 Special Assays or Procedures

The occurrence of neutralizing anti-ancrod antibodies will be determined in blood samples collected at screening, Day 8, and Day 30. Antibody titers will be quantified according to the method described by Vinazzer, 1973.

8.2.4 Biohazard Containment

Not applicable.

8.2.5 Specimen Preparation, Handling and Shipping

Preparation of blood samples for neutralizing anti-ancrod antibodies and for cellular markers and shipment to the central laboratory will be performed according to the analytical requirements of the specific methods.

8.2.6 Instructions for Specimen Storage and Shipment

See separate laboratory instructions

9 Safety Assessments

9.1 Adverse Events

9.1.1 Definition of an Adverse Event (AE) and Adverse Drug Reaction (ADR)

An adverse event is defined as any untoward medical occurrence in a patient or clinical investigation patient administered a pharmaceutical product and which does not necessarily have to have a causal relationship with this treatment.

An AE can therefore be any unfavorable and unintended sign (including an abnormal laboratory finding, for example), symptom, or disease temporally associated with the use of a medicinal product, whether or not considered related to the medicinal product (ICH E2A).

All AEs should be recorded in the CRFs. If an AE has occurred during the period concerned, this should be noted in the appropriate place.

AEs should be carefully monitored during the entire study.

Examples of AEs include one of the following or a combination of 2 or more of the following factors:

- A new sign, symptom, illness, or syndrome.
- Abnormal laboratory values, if judged clinically significant in the opinion of the investigator. (The parameters fibrinogen, TT, PT, aPTT, are to be excluded)
- Worsening (change in nature, severity or frequency) of a concomitant or pre-existing illness.
- An adverse effect of an invasive procedure required by the protocol.
- An accident or injury.

All AEs fall into the categories “non-serious” and “serious”. The judgment “serious” or “non-serious” should be given by the investigator (refer to 9.1.2)

Surgical procedures or other therapeutic interventions themselves are not AEs, but the condition for which the surgery/intervention is required is an AE and should be documented accordingly. Planned surgical measures and the condition(s) leading to these measures are not AEs, if the condition(s) was (were) known before the period of observation and did not worsen during study. In the latter case the condition should be reported as medical history.

Definition of an adverse drug reaction (ADR)

All noxious and unintended responses to an investigational medicinal product related to any dose should be considered adverse drug reactions (ADRs). The phrase "responses to investigational medicinal products" means that a causal relationship between the investigational medicinal product

and an AE is at least a reasonable possibility that means that there are facts (evidence) or arguments to suggest a causal relationship.

9.1.2 Definition of a Serious Adverse Event (SAE)

A serious adverse event (SAE) or reaction is any untoward medical occurrence that at any dose:

- Results in death.
- Is life-threatening.

  NOTE: The term "life-threatening" in the definition of "serious" refers to an event in which the patient was at risk of death at the time of the event; it does not refer to an event which hypothetically might have caused death if it were more severe.
- Requires inpatient hospitalization or prolongation of existing hospitalization.
- Results in persistent or significant disability/incapacity,
- Or is a congenital anomaly/birth defect.
- Some medical events may jeopardize the patient or may require an intervention to prevent one of the above characteristics/consequences. Such events (hereinafter referred to as ‘important medical events’) should also be considered as “serious” in accordance with the definition.

9.1.3 Adverse Event Reporting Period

Subjects will be monitored for AEs after first administration of the study drug through Day 90. All reportable AEs will be followed up until satisfactory resolution or until the investigator considers the event to be chronic or the participant to be stable.

SAEs occurring to a patient after the treatment of that patient has ended should be reported to the sponsor if the investigator becomes aware of them.

9.1.4 Seriousness and Intensity

The assessment “serious” or “non-serious” should be given by the investigator according to the definitions above (9.1.2).

The assessment should include the intensity of the event, whether clinical or laboratory.

Intensity will be assigned using a protocol-defined grading system:

– Mild: events require minimal or no treatment and do not interfere with the patient’s daily activities.
– Moderate: events result in a low level of inconvenience or concern with the therapeutic measures. Moderate events may cause some interference with functioning.

- Severe: events interrupt a patient's usual daily activity and may require systemic drug therapy or other treatment. Severe events are usually incapacitating.
- Life threatening: Any adverse drug experience that places the patient or participant, in the view of the investigator, at immediate risk of death from the reaction as it occurred, i.e., it does not include a reaction that had it occurred in a more severe form, might have caused death.
- Death.

Changes in the severity of an AE should be documented to allow an assessment of the duration of the event at each level of intensity to be performed. AEs characterized as intermittent require documentation of the onset and duration of each episode.

9.1.5 Causality (Relationship) Assessment

Assessment of a relationship between an AE/SAE and the clinical study's medicine is required. The assessment should be done by the investigator and the sponsor.

- A suspected causal relationship ('YES') is given if there is a reasonable possibility of a causal relationship between the event and the study medication. This means that there are facts (evidence) or arguments to suggest a causal relationship.
- If a causal relationship can be ruled out, e.g. because of time sequence, dose-response relationship, Dechallenge and Rechallenge information, pharmacological properties of the drug or alternative explanations (suspected causal relationship = 'NO') the alternative explanation for the event should be provided.
- If the necessary information for assessment of the relationship is not available at the time of the initial report, the SAE can be classified as "not assessable" In that case, the Sponsor will contact the investigator to make up for the assessment.

9.1.6 Eliciting Adverse Event Information

The investigator should report all directly observed AEs and all AEs spontaneously reported by the patient using concise medical terminology. In addition, each study patient will be questioned about AEs at each visit following initiation of treatment. The question asked will be: "Since your last visit/you began taking the investigational medication have you had any health problems?"

9.1.7 Reporting

Complete description of all AEs must be available in the source documents. All AEs including local and systemic reactions not meeting the criteria for SAEs should be captured on the appropriate CRF or electronic data system.

Information to be recorded includes:

- Event description
- Time of onset
- Investigator assessment of seriousness
- Relationship to study product(s)/intervention(s)
- Time of resolution/stabilization of the event.

All AEs occurring while on study must be documented appropriately regardless of relationship.

Any medical condition that is present at the time that the patient is screened should be considered as baseline and not recorded as an AE. However, if the condition deteriorates at any time during the study it should be recorded and reported as an AE.

Changes in the severity of an AE should be documented to allow an assessment of the duration of the event at each level of intensity to be performed. AEs characterized as intermittent require documentation of the onset and duration of each episode.

Changes in the assessment of relationship to the study product(s)/interventions should also be clearly documented.

9.1.8 Recording Instructions

The investigator must document all AEs that occur during the AE reporting period (see Section 9.1.3) on the pages provided in the CRF in accordance with the instructions for the completion of AE reports in clinical studies. These instructions are provided in the investigator's file and the CRF itself.

The following approach will be taken for documentation:

- All AEs including local and systemic reactions must be documented on the "Adverse Event" page of the CRF or electronic data system.
- If the AE is serious, the investigator must complete, in addition, an "SAE Report Form" at the time the SAE is detected. This form must be sent immediately, i.e. within 24 hours after knowledge of the event, to Pharmacovigilance at Nordmark (see Section 9.1.9).

9.1.9 Specific Serious Adverse Event Requirements

The investigator has to report all SAEs immediately (within 24 hours after knowledge of the event) to the sponsor with the exception of those that are identified as not requiring immediate reporting in the protocol or the IB. In that case, the investigator should notify the sponsor within 3 working days.

An event need not be reported as an SAE if it represents only a relapse or an expected change or progression of the condition that was the cause of treatment without any other symptoms and signs than those present before treatment. This type of event needs only to be reported as an AE.

All other (non-serious) AEs and laboratory abnormalities identified in the protocol as critical to safety evaluations should be reported to the sponsor after finishing of the study.

All SAEs will be:

- Recorded on the appropriate SAE Report Form, and in the CRF
- Followed through resolution by a study physician.
- Reviewed by a study physician.

Any AE that meets the protocol-specific expedited (serious) AE reporting criteria must be submitted to the sponsor's pharmacovigilance contractor, at the following address, using the appropriate form*:*

Nordmark Arzneimittel GmbH & Co. KG

Clinical Development / Pharmacovigilance

Pinnauallee 4, 25436 Uetersen, Germany

Mr. Bjoern-Christoph Bartsch

E-Mail: uaw@nordmark-pharma.de

The Sponsor will notify the receipt of the SAE information within 24 hours. Questions about expedited SAE reporting can be referred to the Responsible Person of the Sponsor:

Dr. med. Winrich Rauschning

Phone number: +49 4122 712 926

E-mail: winrich.rauschning@nordmark-pharma.de

or to the Pharmacovigilance officer of the Sponsor

Bjoern-Christoph Bartsch

Phone number: +49 4122 712 555

Mobile: +49 170 566 8789

E-Mail: bjoern-christoph.bartsch@nordmark-pharma.de

At least the following information must be given: Patient code, reporter name, study center, study number, name of the investigational medicinal product, description of the adverse drug reaction or event, duration (start, end), intensity, action taken, outcome, causality and seriousness.

No distinction should be made between the investigational treatment and the reference treatment for AE reporting purposes. In case of suspected serious ADR (refer to 9.1.1), the key data elements as of ICH Guideline E2A (see Annex 17.7) should be collected.

The study clinician will complete an Expedited or Serious Adverse Event Form within the following timelines:

- All SAEs, whether related or unrelated, will be recorded on the Expedited or Serious Adverse Event Form and electronically communicated within 24 hours of site awareness.
- Other supporting documentation of the event may be requested by Pharmacovigilance Nordmark and should be provided as soon as possible.
- All reportable AEs will be followed until satisfactory resolution or until the principal investigator or sub-investigator deems the event to be chronic or the participant to be stable.

9.1.10 Record keeping by the Sponsor

The sponsor will keep detailed records of all adverse events which are reported to him by the investigators.

9.1.11 Regulatory Reporting to the Competent Authorities and the Ethics Committee (EC)

The sponsor is responsible for informing competent authority, ECs and other investigators of SUSAR that have occurred. He will inform the competent authority, ethics committee in expedited manner according to item 37 of the Europeans Commissions guidance CT-3. He will inform other investigators if applicable.

The sponsor will provide once a year during the clinical trial the competent authorities and ethics committees with a listing of all SUSAR which have occurred over this period. This report will follow the guideline ICH E2F on Development Safety Update Report (DSUR).

9.1.12 Exposure in Utero (EIU)

Exposure in-utero (EIU) for investigational products and for marketed products has to be reported and is defined if:

- A female becomes, or is found to be, pregnant either while receiving or having been directly exposed to (e.g., environmental exposure) the investigational product, or the female becomes, or is found to be, pregnant after discontinuing and/or being directly exposed to the investigational product (maternal exposure).
- A male has been exposed, either due to treatment or environmental, to the investigational product prior to or around the time of conception and/or is exposed during his partner's pregnancy (paternal exposure).

If any study patient or study patient's partner becomes or is found to be pregnant during the study patient's treatment with the investigational product, the investigator must submit this information to the sponsor on an “Exposure in Utero Form” provided by the sponsor. In addition, the investigator must submit information regarding environmental exposure to a sponsor product in a pregnant woman (e.g., a nurse reports that she is pregnant and has been exposed to a cytotoxic product by inhalation or spillage) using the Exposure in Utero Form. This must be done irrespective of whether an AE has occurred and immediately after awareness of the pregnancy. The information submitted should include the anticipated date of delivery (see below for information related to induced termination of pregnancy).

Follow-up is conducted to obtain pregnancy outcome information on all EIU reports with an unknown outcome. The investigator will follow the pregnancy until completion or until pregnancy termination (i.e. induced abortion) and then notify the sponsor about the outcome. The investigator will provide this information as a follow up to the initial Exposure in Utero Form. The reason(s) for an induced abortion should be specified. An EIU report is not created when an ectopic pregnancy report is received since this pregnancy is not usually viable. Rather, an SAE case is created with the event of ectopic pregnancy.

If the outcome of the pregnancy meets the criteria for immediate classification as an SAE (i.e. spontaneous abortion, stillbirth, neonatal death, or congenital anomaly [including that in an aborted fetus, stillbirth or neonatal death]), the investigator should follow the procedures for SAE reporting.

In the case of a live birth, the "normality" of the newborn can be assessed at the time of birth (i.e. no minimum follow-up period of a presumably normal infant is required before an exposure in utero form can be completed). The "normality" of an aborted fetus can be assessed by gross visual inspection, unless pre-abortion test findings are suggestive of a congenital anomaly.

Additional information about pregnancy outcomes that are classified as SAEs as follows:

- "Spontaneous abortion" includes miscarriage and missed abortion.
- All neonatal deaths that occur within 1 month of birth should be reported.

In addition, any infant death after 1 month that the investigator assesses as possibly related to the EIU to the investigational medication should be reported.

Additional information regarding the EIU may be requested by the investigator. Further follow-up of birth outcomes will be handled on a case-by-case basis (e. g, follow-up on preterm infants to identify developmental delays). In the case of paternal exposure, the investigator must obtain permission from the patient's partner in order to conduct any follow-up or collect any information.

9.1.13 Follow-Up of Adverse Events

All AEs should be followed until they are resolved or the investigator assesses them as chronic or stable or the patient's participation in the study ends i.e. until a final report is completed for that patient. Instructions for reporting changes in an ongoing AE during a patient's participation in the study are provided in the instructions that accompany the "Adverse Event Case Report Forms".

In addition, all SAEs and those non-serious events assessed by the investigator as possibly related to the investigational medication/product should continue to be followed even after the patient's participation in the study is over. Such events should be followed until they resolve or until the investigator assesses them as "chronic" or "stable." Resolution of such events is to be documented on the appropriate CRF.

9.2 Halting Rules for the Protocol

The sponsor reserves the right to discontinue the study prior to inclusion of the intended number of patients, but intends only to exercise this right for valid scientific or administrative reasons. The study may be prematurely terminated if, in the opinion of the investigator or the sponsor, there is sufficient reasonable cause. Written notification, documenting the reason for study termination, will be provided to the investigator or sponsor by the terminating party.

Circumstances that may warrant termination include, but are not limited to:

- Determination of unexpected, significant, or unacceptable risk to patients.
- Insufficient adherence to protocol requirements.
- Data that are not sufficiently complete and/or evaluable.
- Plans to modify, suspend, or discontinue the development of the study drug.

If the study is prematurely terminated or suspended, the sponsor will promptly inform the investigators/institutions, and the applicable regulatory authority(ies) of the termination or suspension and the reason(s) for the termination or suspension. The IRB/IEC will also be informed

promptly and provided the reason(s) for the termination or suspension by the sponsor or by the investigator/institution, as specified by the applicable regulatory requirement(s).

Further, after such a decision, the investigator must call in all participating patients within 2 weeks for a final examination. At this visit all delivered unused study drug and other study materials must be collected and all case report forms (CRFs) completed as far as possible.

9.3 Stopping Rules for an Individual Participant/Cohort

Patients may be withdrawn from further participation in the study for any the following reasons:

- At their own request without giving reasons (including drop-outs and patients lost to follow-up).
- At the discretion of the investigator, at any time during the study period also in case the investigator considers the patient is not treated appropriately during the course of the study.
- Development of AE, laboratory abnormality, inter-current illness, or other medical condition or situation such that continued participation in the study would not be in the best interest of the patient.
- Meeting any exclusion criteria (either newly developed or not previously recognized).

IMP administration should be interrupted in case of worsening of the health status; medical conditions to be taken into consideration may include but not be limited to pain, gastrointestinal disorders, skin reactions, bleeding of the skin, excretion of blood from intestine or urinary tract or profuse bruises.

If a patient is prematurely withdrawn, a complete final examination is to be performed at the time of withdrawal and the reason for withdrawal has to be documented. Withdrawn patients will not be replaced.

The investigator is responsible for assuring that there are procedures and expertise available to cope with medical emergencies during the study. In the event of severe hemorrhage due to a ancrod overdose, or if major surgery becomes necessary in the course of ancrod therapy, cryoprecipitate or plasma can be administered as a first-line emergency procedure to increase fibrinogen concentrations to safe levels.

To raise fibrinogen levels, the following procedures are recommended:

- Stop administration of ancrod; 50% of the ancrod dose is eliminated within 3-5 hours. The fibrinogen concentration will rise slowly, reaching normal levels within a few days.
- Cryoprecipitate may be administered to raise the plasma fibrinogen concentration to normal levels. The amount of cryoprecipitate required will depend upon the level of ancrod

circulating in the patient. It can be titrated against the fibrinogen concentration, which should be measured within one hour of cryoprecipitate administration.

Decoding is restricted to emergency situations and should only be used under circumstances where knowledge of the treatment is necessary for the proper handling of the patient. If the treatment code envelopes are broken, the reason and the date should be recorded and signed by the investigator.

10 Statistics

10.1 Sample Size Determination

Sample size determination for this study is based on 2-sided t-tests with a global alpha level of 5% and a statistical power of 90%. A standard deviation of 20 dB for the test statistic is assumed. In order to be able to confirm a treatment difference of 15 dB PTA of active treatment versus placebo with a 2:1 randomization, a total number of 87 evaluable patients will be required, 58 patients in the group receiving ancrod, and 29 patients receiving placebo. To compensate for dropouts, a total number of 99 patients is planned to be enrolled, 66 patients receiving ancrod, and 33 patients receiving placebo.

10.2 Statistical Methods

The statistical analysis will be conducted following the principles as specified in the effective ICH Guideline (ICH E9). Complete details of the statistical analyses and methods, including data conventions, will be described in a separate statistical analysis plan (SAP) which will be finalized before unblinding.

For all variables measured at the randomization visit (day 1; visit 1) the last available value prior to the first intake of study medication will be considered as the baseline value. The primary endpoint value is the value measured at day 8 (visit 5).

All efficacy and safety variables will be summarized by treatment group using descriptive statistics (mean, standard deviation [SD], median, minimum, and maximum for continuous data and absolute and relative frequencies for categorical data). Data will be summarized for baseline, endpoint and by visit. The level of significance will be $\alpha = 0.05$.

10.2.1 Method to Evaluate the Primary Efficacy Measure

The primary analysis will be based on the intent-to-treat population (full analysis set); in addition, a per protocol analysis (excluding patients not fulfilling the evaluability criteria) will be done. Evaluability criteria are:

- Have SSHL as defined per inclusion criterion 1
- Provide valid PTA data (Air) for Visit 1 and Visit 5
- Adhere reasonably well to the study protocol without major protocol deviations.

Last-observation-carried-forward (LOCF) will be applied for post-baseline measurements. An analysis of covariance (ANCOVA) model with treatment as fixed effect, center as random effect, and baseline PTA as covariate will be calculated. According to the data structure, it may be advisable to analyze the differences endpoint minus baseline value with a general linear model (GLM).

Underlying assumptions, such as normality and homogeneity of variance will be assessed. Should these assumptions be violated, appropriate data transformations or the choice of a non-parametric analysis method will be considered. Baseline comparability will be evaluated based on pooled data from all study sites. To determine comparability of the ancrod and placebo groups at baseline, demographic and pre-treatment characteristics that are continuous will be analyzed by general linear model (GLM). If treatment group differences are seen at the 0.10 level of significance, these variables may be added as stratification variables or covariates to the efficacy analyses.

All randomized participants in the analysis will be grouped according to their originally assigned treatment.

Additional Analyses of Primary Outcome Measure

The mean change at the affected frequencies will be classified into the following categories:

Definition*	Improvement
Recovery	Hearing level recovers within 30 dB at 250, 500, 1000, 2000 Hz and within 25 dB at 4000 Hz, or<br>Hearing level recovers to that of contralateral intact ear if hearing in intact ear can be considered to be stable
Good	Average hearing improvement for five frequencies ≥ 30 dB
Fair	Average hearing improvement for five frequencies ≥ 10 and ≤ 30 dB
No change	Average hearing improvement for five frequencies is within 10 dB

*Hearing recovery as defined by the Ad Hoc Committee of the Ministry of Health and Welfare in Japan

A comparison of mean changes of participants who completed the study will be made. Multiple linear regression analysis will be performed to evaluate the magnitude of the treatment effects after adjustment for participant characteristics such as age, gender, duration of SSHL prior to treatment, and baseline degree of hearing loss. The response and characteristics of participants who do not complete treatment will be evaluated to assess the missing response pattern.

The mean changes in PTA will be compared for the following subgroups:

- Gender (males or females)
- Degree of SSHL
- SSHL with or without associated vestibular symptoms
- Presence of tinnitus
- Age (above vs. below median, quartiles)
- Status of the contralateral ear
- Duration of SSHL before start of treatment (above vs. below median)
- Baseline fibrinogen level (($\leq$360 mg/dL; >360 mg/dL, quartiles)
- Use of rescue medication.

In addition, the effect of diabetes mellitus, hypertension, and ischemic heart disease will be included in the analysis.

Because the study is not powered to detect significant treatment effects in subgroups, the limitations of drawing conclusions if no treatment effect is observed in a subgroup is recognized.

Methods to evaluate Secondary Efficacy Measures

The quantitative parameters will be analyzed in analogy to the primary efficacy variable. For 2x2-tables, the qualitative parameters will be analyzed with the exact Fisher-test; the Freeman-Halton test will be used for tables that are larger than 2x2 and if a table is very large, analysis will be carried out with the asymptotic chi-square-test.

The semi-quantitative parameters patient and physician assessment of change of hearing ability will be transformed to three qualitative categories. Further details and analyses depend on the CRF structure and will be described in detail in the Statistical Analysis Plan.

Safety Measures

Safety data will be analyzed descriptively by treatment group. Absolute and relative frequencies will be calculated for AEs by system organ class and preferred term. The comparison of AEs between groups will be done by the exact Fisher test.

10.2.2 Expected Side Effects

Reports of bleeding AEs, as well as major bleeding, will be identified and summarized through Day 90 after initiation of study drug. Bleeding AEs will be identified using the standard MedDRA query for hemorrhages. A logistic model will be used to test for treatment group differences in the incidence of major bleeding. The logistic regression model will include terms for treatment group, age, baseline fibrinogen level, and severity of SSHL.

10.2.3 Interim Analysis

The recruitment of the patients will take a longer period than initially expected. For this reason a comparison of groups will be performed as interim analysis for the primary objective if 30 evaluable patients are included in the study.

11 Quality Control (QC) and Quality Assurance (QA)

11.1 Study Monitoring and Auditing

Monitoring and auditing procedures developed or endorsed by the Sponsor will be followed in accordance with GCP guidelines. Direct access to the on-site study documentation and medical records must be ensured.

11.1.1 Study Monitoring and Source Data Verification

Monitoring will be done by personal visits from a representative of the Sponsor or CRO (clinical monitor) that will check the CRFs for completeness and clarity, and crosscheck them with source documents (100% source data verification). Questionnaires completed by patients will be included in the CRF, and there will be no other source documentation available. In addition to the monitoring visits, frequent communications (letter, telephone, and fax) by the clinical monitor will ensure that the investigation is conducted according to protocol design and regulatory requirements.

Study close-out will be performed by the clinical monitor upon closure of the study.

11.1.2 On-Site Audits

Domestic and foreign regulatory authorities, the EC or IRB, and an auditor authorized by the sponsor may request access to all source documents, CRFs, and other study documentation for on-site audit or inspection. Direct access to these documents must be guaranteed by the investigator, who must provide support at all times for these activities. Medical records and other study documents may be copied during audit or inspection provided that patient names are removed on the copies to ensure confidentiality.

12 Ethical and Regulatory Considerations

12.1 Declaration of Helsinki and other regulations

This study is to be conducted according to globally accepted standards of GCP (as defined in the ICH E6 Guideline for GCP), in agreement with the Declaration of Helsinki from 2008 (Appendix 17.8) and in keeping with local regulations.

12.2 Obtaining an EudraCT Number

The EudraCT Number 2012-000066-37 was obtained at the European Agency by the sponsor for this study.

12.3 Institutional Review Board (IRB) / Independent Ethics Committee (IEC)

It is the responsibility of the sponsor to obtain approval of the study protocol and to keep the EC/IRB informed of any SAEs and amendments to the protocol. All correspondence with the ethics EC/IRB should be filed by the sponsor in the study master file and copies will be forwarded to the principal investigators of participating centers for filing in the site master file.

12.4 Patient Information and Informed Consent

It is the responsibility of the investigator to give each patient (or the patient's legally authorized representative) prior to inclusion in the study, full and adequate verbal and written information regarding the objective and procedures of the study and the possible risks and benefits involved. The patients must be informed about their right to withdraw from the study at any time. Written patient information should be given to each patient before enrollment. The written patient information must not be changed without prior discussion with the sponsor/coordinating investigator. Furthermore, it is the responsibility of the investigator to obtain signed informed consent from all patients prior to inclusion in the study.

The signed informed consent forms should be filed by the investigator for possible review by monitors or audits by competent authorities or Sponsor. The investigator will confirm the receipt of informed consent from each patient by signing the appropriate page of the CRF.

12.5 Liability and Insurance

Liability and insurance provisions for patients and investigators participating in this study are given in separate agreements.

12.6 Approval of competent authorities and Announcement to Local Authority

The sponsor will obtain approval of the study protocol/amendments from the responsible competent authorities (Germany - Bundesinstitut für Arzneimittel und Medizinprodukte [BfArM], Czech Republic- Státní ústav pro kontrolu léčiv [SUKL]) before starting any study-related procedures. Correspondence with competent authority and the written approval will be filed by the sponsor in the trial master file.

In Germany the clinical study will be announced to the local authority (Bezirksregierung) in accordance with § 67 German Drug Law (AMG) by the sponsor. A copy of this letter will be filed in the trial master file. An announcement to local authorities in the other countries is not necessary.

12.7 Curriculum Vitae and other Documentation

Curriculum vitae must be obtained from all investigators. It should include name, title, occupation, education (including valid GCP-trainings, clinical and research experience, date of license to practice medicine “Approbation”), present and former positions and should be signed and dated.

All documentation required for ethical, administrative, regulatory and other purposes not described elsewhere in the protocol, e.g. curriculum vitae, statement of investigator, signature and delegation list etc.

13 Data Handling and Record Keeping

13.1 Protocol Review

The study will not be started until approval of the protocol, the patient information and the informed consent form has been obtained from the appropriate ECs/IRBs and the competent authorities. It is the responsibility of the sponsor to forward a copy of the written approval and, where possible, a list of the members, their titles or occupation, and their institutional affiliations, to the principal

investigators of participating centers and to the coordinating investigator. The approval should include the study identification and the date of review.

The clinical study requires application for approval/notification from/to the competent authorities. The study will not be started until receipt by Nordmark of written approval/copy of application letters. Nordmark should provide the investigators with a copy of the relevant document.

13.2 Changes to Final Study Protocol

Any variation in procedure from that specified in the final study protocol may lead to the results of the study being questioned and in some cases rejected. Any proposed protocol change must therefore be discussed with Nordmark and approved by Nordmark and submitted for EC/IRB and competent authority approval. Any relevant protocol change should be documented in a protocol amendment."

13.3 Case Report Forms (CRFs)

CRFs of a design mutually agreed upon by the coordinating investigator and the sponsor were compiled and supplied by Medizinisches Wirtschaftsinstitut GmbH (MWI). X-act took over the supply. With Protocol Amendment 7.0, patient study data will be documented in a paper CRF (NCR = No Carbon Required) instead of using mCRF®s.

A CRF is required and should be completed for each included patient and signed by the investigator. Data management services were also provided by the sponsor's contracted biometric institute MWI and handed over to X-act. After receipt of the mCRF®s the prompt runback and the electronic signature will be checked by X-Act. For paper CRFs, the Investigator's signature will be verified by the monitor. After monitoring, one copy of the paper CRF will be filed at the site, one copy will be sent to X-act, and the original CRF will be filed in the sponsor's Site Master file.

Before data entry, all CRFs will be examined for complete and thorough content. The data from the signed and dated mCRF®s/paper CRFs will be transferred to the data bank by GCP certified data entry workers. Following the data entry, the efficacy and validity review will utilize query processes to identify non-efficacy or invalid data from the data bank (i.e. CRFs). Any implausibility will be discussed with the study site, if necessary. Corrections and revisions of the electronically entered data will be documented on the data log file, along with the revising person's name and reason for data change/revision. If a center changes the data contained in a mCRF® file after its having been sent, the investigator has to resend said mCRF® file in a signed and dated version. If corrections are performed by another member of the staff, the investigator has to approve the correction. Upon

completion of the last data changes and revisions, the completed data bank with be closed and released for statistical analyses.

The completed original CRFs are the sole property of Nordmark and should not be made available in any form to third parties, except for authorized representatives of competent authorities, without permission. The paper CRFs should be made available for collection by the monitor as agreed.

13.4 Patient Data Protection

The investigator should keep a patient identification list, not to be made available to the sponsor, including sufficient information to link records, i.e. CRF and hospital records.

The patients should be informed that the data will be stored in pseudonymized form and analyzed by computer that international and local regulations for the handling of computerized data will be followed and described in the written patient information and that identification of individual patient data will only be possible for the investigator. Furthermore, the patients should be informed about the possibility of inspection of relevant parts of the hospital records by representatives of Nordmark or a contracted agent/CRO (monitors, auditors) and/or competent authority.

13.5 Record Retention

According to ICH-GCP study records as defined in ICH-GCP (section 8 essential documents) should be retained by the permit holder until at least 2 years after the last approval of a marketing application in an ICH region and until there are no pending or contemplated marketing applications in an ICH region or at least 2 years have elapsed since the formal discontinuation of clinical development of the investigational product. However, these documents should be retained for a longer period if required by the applicable regulatory requirements.

To enable any further evaluations and/or audits from competent authorities/CRO/the Sponsor, the investigator agrees to keep records, including the identity of all participating patients (sufficient information to link records, i.e. CRF and hospital records) like identification code list and enrolment log and all original signed informed consent forms for at least 15 years after the completion or discontinuation of the study whatever is the longest period in time. All other essential documents (defined in section 8, ICH-GCP) like copies of CRFs and detailed records of drug disposition must be retained by the investigator for 10 years after study termination.

The investigator must obtain approval in writing from the Sponsor before destruction of any records, and must document any change of ownership.

It is the responsibility of the Sponsor to inform the investigator/institution as to when these documents no longer need to be retained.

If an investigator leaves an investigational site, the responsibility for archiving of all study related records has to be transferred to another person (e.g. other investigator). The Sponsor has to be informed about any change in responsibility.

14 Publications

All information not previously published concerning the test product and Nordmark's research, including patent applications, manufacturing processes, basic scientific data etc. is considered confidential and should remain the sole property of the sponsor. Each investigator agrees to use this information only in connection with this study and will not use it for other purposes without written permission from the sponsor.

After completion of the study, the statistical analysis will be performed by X-act. The results will be presented to the investigator. Based on these data a clinical study report will be prepared in cooperation with the investigator. The report should be submitted to competent authorities and ECs/IRBs according to local regulations (within 1 year after completion of the study) and will form the basis for a manuscript intended for publication in a medical journal.

The first publication should be a joint publication, reporting the combined results from all centers. It is agreed that prior to publication the sponsor will be given the opportunity to review and comment upon the manuscript. The time for review should not exceed 30 days after receipt of the manuscript. If the investigator has not submitted the results for publication within 6 months after completion of the final clinical study report, the sponsor will have the right to publish. In this case the investigators will be given 30 days to review and comment on the manuscript prior to submission to the publisher.

It is agreed between the investigator and the sponsor that data from the study will be used in connection with the development of the study drug. Information may therefore be disclosed as required to other investigators and to competent authority.

The study will be registered at www.clinicaltrials.gov to enable publication of the study results.

15 Financial Support

The study will be supported and funded by Nordmark Arzneimittel GmbH & Co. KG.

16 Reference List

Bell W; 1987; Defibrinogenating enzymes. Coleman R, et al. (eds.), Hemostasis and Thrombosis, J.P. Lippincott, Philadelphia, Chapter 54, 886-900.

Chau J, Lin J, Atashband S, Irvine R, Westerberg B; 2010; Systematic review of the evidence for the etiology of adult sudden sensorineural hearing loss. The Laryngoscope;120:1011-1021.

Conlin A, Parnes L; 2007a; Treatment of sudden sensorineural hearing loss: I. A systematic review. Archives of Otolaryngology--Head & Neck Surgery;133:573-581.

Conlin A, Parnes L; 2007b; Treatment of sudden sensorineural hearing loss II. A Meta-analysis. Archives of Otolaryngology--Head & Neck Surgery;133:582-586.

Ehrly A; 1973; Influence of Arwin on the flow properties of blood. Biorheology;10(3):453-456.

Ernst E, Dormandy J; 1981; The effects of Arvin and surgery on red cell filterability. Scand J Clin Lab Invest Suppl;156:317-319.

DIN 45621 Sprache für Gehörprüfung Teil 1: Ein-und mehrsilbige Wörter

Finger R P, Gostian AO; 2006; Apheresis for idiopathic sudden hearing loss: reviewing the evidence. Journal of Clinical Apheresis;21:241-245.

ICH E2A: Clinical safety data management. Definitions and standards for expedited reporting.

ISO 7029; Akustik – Statistische Verteilung von Hörschwellen als eine Funktion des Alters

ISO 8253-1; Akustik – Audiometrische Prüfverfahren – Teil 1: Grundlegende Verfahren der Luft- und Knochenleitungs-Schwellenaudiometrie mit reinen Tönen

Kassner SS, Schöttler S, Bonaterra GA, Stern-Sträter J, Sommer U, Hörmann K, Kinscherf R, Gössler UR; 2011; Audiology & Neurotology:16:254-262.

Kubo T, Matsunaga T, Asai H, Kawamoto K, Kusakari J, Nomura Y, Oda M, Yanagita N, Niwa H, Uemura T. et al.; 1988; Efficacy of defibrinogenation and steroid therapies on sudden deafness. Archives of Otolaryngology--Head & Neck Surgery;114:649-652.

Latallo Z S; 1983; Retrospective study on complications and adverse effects of treatment with thrombin-like enzymes -- a multicentre trial. Thrombosis and Haemostasis;50:604-609.

Michel O; 1994; Der Hörsturz. Thieme.

Lowe GDO, Campbell AF, Meek DR, Forbes CD; 1978; Subcutaneous ancrod in prevention of deep vein thrombosis after operation for fractured neck of femur. Lancet;30:698-700.

NIH publication; 2000; Sudden deafness. Bethesda: National Institute of Health; http://www.nidcd.nih.gov/health/hearing/sudden.asp, NIH publication 00-4757.

Quaranta,N, Ramunni A, DeLuca C, Brescia P, Dambra P, DeTullio G, Vacca A, Quaranta A; 2011; Endothelial progenitor cells in sudden sensorineural hearing loss. Acta Oto-Laryngologica:131:347-350.

Quaranta,N, Ramunni A, Brescia P, D'Elia A, Vacca A, Ria R. 2008;Soluble intercellular adhesion molecule 1 and soluble vascular cell adhesion molecule 1 in sudden hearing loss. Otol Neurotol:29:470-474

Ryals B, Rubel E; 1988; Hair cell regeneration after acoustic trauma in adult Coturnix quail. Science;240:1774-1776.

Shiraishi T, Kubo T, Okumura S, Naramura H, Nishimura M, Okusa M, Matsunaga T; 1993; Hearing recovery in sudden deafness patients using a modified defibrinogenation therapy. Acta Oto-Laryngologica. Suppl.;501:46-50.

Suckfüll M, Wimmer C, Reichel O, Mees K, Schorn, K; 2002; Hyperfibrinogenemia as a risk factor for sudden hearing loss. Otology & Neurotology : Official Publication of the American Otological Society, American Neurotology Society [and] European Academy of Otology and Neurotology;23: 309-311.

Suckfüll M; 2009; Perspectives on the pathophysiology and treatment of sudden idiopathic sensorineural hearing loss. Dtsch Arztebl Int;106:669-675;quiz 676.

Suzuki H, Furukawa M, Kumagai M, Takahashi E, Matsuura K, Katori Y, Shimomura A, Kobayashi T; 2003; Defibrinogenation therapy for idiopathic sudden sensorineural hearing loss in comparison with high-dose steroid therapy. Acta Oto-Laryngologica;123:46-50.

Vinazzer H; 1973; Acquired resistance to ancrod. Its evaluation and clinical occurrence. Thromb Diath Haemorrh: 29(2):339-46

Wei BPC, Mubiru S, O'Leary S; 2006; Steroids for idiopathic sudden sensorineural hearing loss. Cochrane Database of Systematic Reviews;1:CD003998.

Wei, BPC, Mubiru S., O'Leary, S., (2006, last assessed as up-to-date July 6, 2009) Steroids for idiopathic sensorineural hearing loss. Cochrane Database of Systematic Reviews 2006, Issue 1 Art. No: CD003998. DOI: 10.1002/14651858. CD003998.pub2.

Wilson W, Byl F, Laird N; 1980; The efficacy of steroids in the treatment of idiopathic sudden hearing loss. Archives of Otolaryngology;106:772-776.

Wolf GK; 1976: Arwin in peripheral arterial circulatory disorders: Controlled multicenter trials. Europ J Clin Pharmacol; 9:387-392.

Zhang L, Hong Lu S,Li L, Tao Y-G, Wan YL, Senga H, Yang R, Han ZH; 2011:Batroxobin mobilizes circulating endothelial progenitor cells in patients with deep vein thrombosis. Clin Appl Thromb Hemost.;17(1):75-79.

17 List of Appendices

17.1 Patient Log/Identification List

17.2 Patient Screening and Enrollment Log

17.3 Signature and Delegation List/Log

17.4 Dosing Worksheet

17.5 Drug Accountability Form

17.6 Study Schedule

17.7 Key data elements for inclusion in expedited reports of serious adverse drug reactions (attachment 1 of ICH Guideline E2A)

17.8 World Medical Association, Declaration of Helsinki

17.1 Patient Log/Identification List

Title of the study	Double-blind, randomized, placebo-controlled study on efficacy, safety and tolerability of ancrod in patients with sudden sensorineural hearing loss (SSHL)
Study Number: NM V-101	EudraCT number: 2012-000066-37
Center (Number and address):	

**Pat. No.**	**Full name**	**Birth date**	**Sex f/m**	**Date of informed consent**	**Comments**

17.2 Patient Screening and Enrollment Log

Title of the study	Double-blind, randomized, placebo-controlled study on efficacy, safety and tolerability of ancrod in patients with sudden sensorineural hearing loss (SSHL)
Study Number: NM V-101	EudraCT number: 2012-000066-37
Center (Number and address):	

**Screening No.**	**Date of Screening**	**Date of IC**	**Sex f/m**	**Inclusion Yes/no**	**Reason for not including**

17.3 Signature and Delegation List/Log

Title of the study	Double-blind, randomized, placebo-controlled study on efficacy, safety and tolerability of ancrod in patients with sudden sensorineural hearing loss (SSHL)
Study Number: NM V-101	EudraCT number: 2012-000066-37
Center (Number and address):	

Signature list with full names and initials of all persons authorized to sign or make corrections in CRFs or other study documents. The list has to be updated regularly.

Delegated tasks are documented in the form: "Qualification of study group members"

**Principal or Coordinating Investigator:**	
Title and name:	Signature:
Initials:	Date:
**Co-investigator:**	
Title and name:	Signature:
Initials:	Date:
**Co-investigator:**	
Title and name:	Signature:
Initials:	Date:
**Co-investigator.**	
Title and name:	Signature:
Initials:	Date:

**Other members of the study team:**	
Title and name:	Signature:
Initials:	Date:
Specify the function:	

Title of the study	Double-blind, randomized, placebo-controlled study on efficacy, safety and tolerability of ancrod in patients with sudden sensorineural hearing loss (SSHL)
Study Number: NM V-101	EudraCT number: 2012-000066-37
Center (Number and address):	

17.4 Dosing Worksheet

**Patient No**							
**Patient weight (Kg)**							
	**Date**	**Infusion Rate (ml/h) 0.1193 x Weight**	**Injection Volume (ml) 0.0143 x Weight**	**Time of preparation**	**Time of start infusion**	**Time of injection**	**Initials**
**Infusion Day 1**			NA			NA	
**s.c. Injection Day 2**		NA			NA		
**s.c. Injection Day 4**		NA			NA		
**s.c. Injection Day 6**		NA			NA		

17.5 Drug Accountability Form

Title of the study	Double-blind, randomized, placebo-controlled study on efficacy, safety and tolerability of ancrod in patients with sudden sensorineural hearing loss (SSHL)
Study Number: NM V-101	EudraCT number: 2012-000066-37
Center (Number and address):	

Drug Accountability for Patient No.

Delivery from **to:**

**Date of receipt**	**Batch Number**	**Patient-Number**	**Study episode (e.g. Blind phase)**	**Number of boxes**	**Expiry Date**	**Condition (OK/damaged))**	**Replace-ment delivery necessary (Y/N)***	**Name and Signature (Investigator)**

Study Drug dispensing from name and function: **to patient**

Date of dispensing	Batch Number	Patient-Number	Study episode (e.g. Blind phase)	Number of boxes	Expiry Date	Other information (e.g. Number of tablets etc)	Name an Signature (Investigator)

Return of Study Drug dispensing patient to

name and function:

Date of Return	Batch Number	Patient-Number	Study episode (e.g. Blind phase)	Number of boxes	Expiry Date	Used/unused	Name an Signature (Investigator)

17.6 Study Schedule

	Screening	Randomi-zation	Start treatment	Double-blind Treatment Phase				Follow-up	
**Day**	1 (may take 36 hours)			2	4 (+1)	6 (+1)	8 (±1)	30 (±one week	90 (±one week
**Visit**	1			2	3	4[a]	5[a]	6	7
Informed consent	✓								
Inclusion/exclusion criteria	✓								
Demographics, baseline characteristics	✓								
Pregnancy test[b]	✓								
Medical history	✓								
Previous medication[c]	✓								
Physical and otolaryngological examination[d]	✓						✓		
Coagulation[e]	✓*			✓**[f]			✓[f]		
Clinical Chemistry[g]	✓						✓		
Hematology[h]	✓						✓		
Urinalysis[i]	✓						✓		
Audiometric evaluation[j]	✓						✓	✓	✓
Randomization		✓							
Study drug administration			✓	✓	✓[k]	✓[k]			
Concomitant medication	✓			✓	✓	✓	✓	✓	✓
Biomarker[l]	✓**						✓**		
neutralizing anti-ancrod antibodies[m]	✓**						✓**	✓**	
Vital signs	✓						✓		
Nystagmography/Frencl's goggles	✓						✓		
Tinnitus	✓						✓	✓	✓
NRS Patient/Physician assessment	✓						✓	✓	✓
Adverse events[n]			✓	✓	✓	✓	✓	✓	✓

a: Visit 4 and 5 are to be performed on different days

b: Performed in premenopausal women who have not been surgically sterilized (documented complete hysterectomy or bi-tubal ligations) or post-menopausal >1 year. Analysis in urine or serum, study drug may not be administered before obtaining pregnancy test results

c: All medication taken within the last 30 days before Visit 1 should be documented

d: Physical examination including general appearance (including lymphatic nodes),HEENT (head, eyes, ears, nose and throat), cardiovascular, respiratory, gastrointestinal/abdomen, musculoskeletal, skin/dermatological, neurologic and other examinations

Otolaryngological examination including otoacustic emissions, tympanometry, stapedius reflex measurement and ear microscopy

e: Parameters to be determined in the selected laboratory (local or central) of the site for safety reasons: Fibrinogen, TT, PT, aPTT.

*The initial dose of study drug will be adjusted according to the screening fibrinogen concentration assessed in the local laboratory.

**The assay on day 2 will be performed within 36 hours in the selected laboratory of the site.

f: The investigator must announce the taking of the blood sample on day 2 for fibrinogen one day before per fax Nordmark and if necessary to the courier company. Nordmark has to be immediately informed in writing (fax) from the responsible laboratory or designee on the result of the coagulation assays on day 2 and 8. The result of the coagulation assay may in principle not be communicated from the laboratory to the investigator or any other blinded study personnel.

g: Responsible laboratory: Creatinine, aspartate aminotransferase (SGOT; AST), alanine aminotransferase (SGPT; ALT), sodium, potassium, chloride, glucose, urea, uric acid, phosphorus, total bilirubin, total protein, albumin, lactate dehydrogenase (LDH), gamma-glutamyltransferase (γ-GT), alkaline phosphatase, creatinine phosphokinase, triglyceride, cholesterol, high density lipoprotein (HDL), low density lipoprotein (LDL), and very low density lipoprotein (VLDL).

h: Responsible laboratory: Erythrocytes, hematocrit, MCV, leucocytes, erythrocyte sedimentation rate, hemoglobin, thrombocytes.

i: Responsible laboratory: Hemoglobin or blood in urine, pH, specific gravity, protein, glucose, ketones, bilirubin, leucocytes.

j: Air/bone PTA and speech intelligibility test inclusive word recognition

k: Study drug on Day 4 and 6 may only be administered after the investigator has been informed by ProjectPharm that the fibrinogen concentration on Day 2 is ≥ 50 mg/dl.

l: **Central laboratory

m: **Central laboratory

n: If an AE is reported as ongoing at day 90, an additional follow-up will be performed four weeks (± 1 week) later by telephone. If the ongoing AE at day 90 involves a laboratory abnormality, an extra visit will occur four weeks (± 1 week) later for assessment of laboratory results.

17.7 Key data elements for inclusion in expedited reports of serious adverse drug reactions (taken from attachment 1 of ICH Guideline E2A)

The following list of items has its foundation in several established precedents, including those of CIOMS-I, the WHO International Drug Monitoring Centre, and various regulatory authority forms and guidelines. Some items may not be relevant depending on the circumstances. The minimum information required for expedited reporting purposes is: an identifiable patient, the name of a suspect medicinal product, an identifiable reporting source, and an event or outcome that can be identified as serious and unexpected and for which, in clinical investigation cases, there is a reasonable suspected causal relationship. Attempts should be made to obtain follow-up information on as many other listed items pertinent to the case.

1. Patient Details

- Other relevant identifier (clinical investigation number, for example)
- Gender
- date of birth
- Weight

2. Suspected Investigational Medicinal Product(s)

- International Non-Proprietary Name (INN)
- Batch number
- Daily dose and regimen (specify units - e.g., mg, ml, mg/kg)
- Route of administration
- Starting date and time of day
- Stopping date and time, or duration of treatment

3. Other Treatment(s)

- For concomitant medicinal products (including non-prescription/OTC medicinal products) and non-medicinal product therapies, provide the same information as for the suspected product.

4. Details of Suspected Adverse Drug Reaction(s)

- Full description of reaction(s) including body site and intensity, as well as the criterion (or criteria) for regarding the report as serious should be given. In addition to a

description of the reported signs and symptoms, whenever possible, attempts should be made to establish a specific diagnosis for the reaction.

- Start date (and time) of onset of reaction
- Stop date (and time) or duration of reaction
- Dechallenge and rechallenge information
- Setting (e.g., hospital, out-patient clinic, home, nursing home)
- Outcome: information on recovery and any sequelae; what specific tests and/or treatment may have been required and their results; for a fatal outcome, cause of death and a comment on its possible relationship to the suspected reaction should be provided. Any autopsy or other post-mortem findings (including a coroner's report) should also be provided when available. Other information: anything relevant to facilitate assessment of the case, such as medical history including allergy, drug or alcohol abuse; family history; findings from special investigations.

5. Details on Reporter (Investigator) of Event (Suspected ADR)

- Name
- Address
- Telephone number
- Profession (speciality)

17.8 World Medical Association Declaration of Helsinki

Ethical Principles for Medical Research Involving Human Subjects

Adopted by the 18th WMA General Assembly, Helsinki, Finland, June 1964, and amended by the:
29th WMA General Assembly, Tokyo, Japan, October 1975
35th WMA General Assembly, Venice, Italy, October 1983
41st WMA General Assembly, Hong Kong, September 1989
48th WMA General Assembly, Somerset West, Republic of South Africa, October 1996
52nd WMA General Assembly, Edinburgh, Scotland, October 2000
53rd WMA General Assembly, Washington 2002 (Note of Clarification on paragraph 29 added)
55th WMA General Assembly, Tokyo 2004 (Note of Clarification on Paragraph 30 added)
59th WMA General Assembly, Seoul, October 2008

A. INTRODUCTION

1. The World Medical Association (WMA) has developed the Declaration of Helsinki as a statement of ethical principles for medical research involving human subjects, including research on identifiable human material and data.
   The Declaration is intended to be read as a whole and each of its constituent paragraphs should not be applied without consideration of all other relevant paragraphs.
2. Although the Declaration is addressed primarily to physicians, the WMA encourages other participants in medical research involving human subjects to adopt these principles.
3. It is the duty of the physician to promote and safeguard the health of patients, including those who are involved in medical research. The physician's knowledge and conscience are dedicated to the fulfilment of this duty.
4. The Declaration of Geneva of the WMA binds the physician with the words, “The health of my patient will be my first consideration,” and the International Code of Medical Ethics declares that, “A physician shall act in the patient's best interest when providing medical care.”
5. Medical progress is based on research that ultimately must include studies involving human subjects. Populations that are underrepresented in medical research should be provided appropriate access to participation in research.
6. In medical research involving human subjects, the well-being of the individual research subject must take precedence over all other interests.
7. The primary purpose of medical research involving human subjects is to understand the causes, development and effects of diseases and improve preventive, diagnostic and therapeutic interventions (methods, procedures and treatments). Even the best current

interventions must be evaluated continually through research for their safety, effectiveness, efficiency, accessibility and quality.

8. In medical practice and in medical research, most interventions involve risks and burdens.
9. Medical research is subject to ethical standards that promote respect for all human subjects and protect their health and rights. Some research populations are particularly vulnerable and need special protection. These include those who cannot give or refuse consent for themselves and those who may be vulnerable to coercion or undue influence.
10. Physicians should consider the ethical, legal and regulatory norms and standards for research involving human subjects in their own countries as well as applicable international norms and standards. No national or international ethical, legal or regulatory requirement should reduce or eliminate any of the protections for research subjects set forth in this Declaration.

B. PRINCIPLES FOR ALL MEDICAL RESEARCH

11. It is the duty of physicians who participate in medical research to protect the life, health, dignity, integrity, right to self-determination, privacy, and confidentiality of personal information of research subjects.
12. Medical research involving human subjects must conform to generally accepted scientific principles, be based on a thorough knowledge of the scientific literature, other relevant sources of information, and adequate laboratory and, as appropriate, animal experimentation. The welfare of animals used for research must be respected.
13. Appropriate caution must be exercised in the conduct of medical research that may harm the environment.
14. The design and performance of each research study involving human subjects must be clearly described in a research protocol. The protocol should contain a statement of the ethical considerations involved and should indicate how the principles in this Declaration have been addressed. The protocol should include information regarding funding, sponsors, institutional affiliations, other potential conflicts of interest, incentives for subjects and provisions for treating and/or compensating subjects who are harmed as a consequence of participation in the research study. The protocol should describe arrangements for post-study access by study subjects to interventions identified as beneficial in the study or access to other appropriate care or benefits.
15. The research protocol must be submitted for consideration, comment, guidance and approval to a research ethics committee before the study begins. This committee must be

independent of the researcher, the sponsor and any other undue influence. It must take into consideration the laws and regulations of the country or countries in which the research is to be performed as well as applicable international norms and standards but these must not be allowed to reduce or eliminate any of the protections for research subjects set forth in this Declaration. The committee must have the right to monitor ongoing studies. The researcher must provide monitoring information to the committee, especially information about any serious adverse events. No change to the protocol may be made without consideration and approval by the committee.

16. Medical research involving human subjects must be conducted only by individuals with the appropriate scientific training and qualifications. Research on patients or healthy volunteers requires the supervision of a competent and appropriately qualified physician or other health care professional. The responsibility for the protection of research subjects must always rest with the physician or other health care professional and never the research subjects, even though they have given consent.
17. Medical research involving a disadvantaged or vulnerable population or community is only justified if the research is responsive to the health needs and priorities of this population or community and if there is a reasonable likelihood that this population or community stands to benefit from the results of the research.
18. Every medical research study involving human subjects must be preceded by careful assessment of predictable risks and burdens to the individuals and communities involved in the research in comparison with foreseeable benefits to them and to other individuals or communities affected by the condition under investigation.
19. Every clinical trial must be registered in a publicly accessible database before recruitment of the first subject.
20. Physicians may not participate in a research study involving human subjects unless they are confident that the risks involved have been adequately assessed and can be satisfactorily managed. Physicians must immediately stop a study when the risks are found to outweigh the potential benefits or when there is conclusive proof of positive and beneficial results.
21. Medical research involving human subjects may only be conducted if the importance of the objective outweighs the inherent risks and burdens to the research subjects.
22. Participation by competent individuals as subjects in medical research must be voluntary. Although it may be appropriate to consult family members or community leaders, no competent individual may be enrolled in a research study unless he or she freely agrees.

23. Every precaution must be taken to protect the privacy of research subjects and the confidentiality of their personal information and to minimize the impact of the study on their physical, mental and social integrity.
24. In medical research involving competent human subjects, each potential subject must be adequately informed of the aims, methods, sources of funding, any possible conflicts of interest, institutional affiliations of the researcher, the anticipated benefits and potential risks of the study and the discomfort it may entail, and any other relevant aspects of the study. The potential subject must be informed of the right to refuse to participate in the study or to withdraw consent to participate at any time without reprisal. Special attention should be given to the specific information needs of individual potential subjects as well as to the methods used to deliver the information. After ensuring that the potential subject has understood the information, the physician or another appropriately qualified individual must then seek the potential subject's freely-given informed consent, preferably in writing. If the consent cannot be expressed in writing, the non-written consent must be formally documented and witnessed.
25. For medical research using identifiable human material or data, physicians must normally seek consent for the collection, analysis, storage and/or reuse. There may be situations where consent would be impossible or impractical to obtain for such research or would pose a threat to the validity of the research. In such situations the research may be done only after consideration and approval of a research ethics committee.
26. When seeking informed consent for participation in a research study the physician should be particularly cautious if the potential subject is in a dependent relationship with the physician or may consent under duress. In such situations the informed consent should be sought by an appropriately qualified individual who is completely independent of this relationship.
27. For a potential research subject who is incompetent, the physician must seek informed consent from the legally authorized representative. These individuals must not be included in a research study that has no likelihood of benefit for them unless it is intended to promote the health of the population represented by the potential subject, the research cannot instead be performed with competent persons, and the research entails only minimal risk and minimal burden.
28. When a potential research subject who is deemed incompetent is able to give assent to decisions about participation in research, the physician must seek that assent in addition to

the consent of the legally authorized representative. The potential subject's dissent should be respected.

29. Research involving subjects who are physically or mentally incapable of giving consent, for example, unconscious patients, may be done only if the physical or mental condition that prevents giving informed consent is a necessary characteristic of the research population. In such circumstances the physician should seek informed consent from the legally authorized representative. If no such representative is available and if the research cannot be delayed, the study may proceed without informed consent provided that the specific reasons for involving subjects with a condition that renders them unable to give informed consent have been stated in the research protocol and the study has been approved by a research ethics committee. Consent to remain in the research should be obtained as soon as possible from the subject or a legally authorized representative.
30. Authors, editors and publishers all have ethical obligations with regard to the publication of the results of research. Authors have a duty to make publicly available the results of their research on human subjects and are accountable for the completeness and accuracy of their reports. They should adhere to accepted guidelines for ethical reporting. Negative and inconclusive as well as positive results should be published or otherwise made publicly available. Sources of funding, institutional affiliations and conflicts of interest should be declared in the publication. Reports of research not in accordance with the principles of this Declaration should not be accepted for publication.

C. ADDITIONAL PRINCIPLES FOR MEDICAL RESEARCH COMBINED WITH MEDICAL CARE

31. The physician may combine medical research with medical care only to the extent that the research is justified by its potential preventive, diagnostic or therapeutic value and if the physician has good reason to believe that participation in the research study will not adversely affect the health of the patients who serve as research subjects.
32. The benefits, risks, burdens and effectiveness of a new intervention must be tested against those of the best current proven intervention, except in the following circumstances:
    • The use of placebo, or no treatment, is acceptable in studies where no current proven intervention exists; or
    • Where for compelling and scientifically sound methodological reasons the use of placebo is necessary to determine the efficacy or safety of an intervention and the patients who receive

placebo or no treatment will not be subject to any risk of serious or irreversible harm. Extreme care must be taken to avoid abuse of this option.

33. At the conclusion of the study, patients entered into the study are entitled to be informed about the outcome of the study and to share any benefits that result from it, for example, access to interventions identified as beneficial in the study or to other appropriate care or benefits.
34. The physician must fully inform the patient which aspects of the care are related to the research. The refusal of a patient to participate in a study or the patient's decision to withdraw from the study must never interfere with the patient-physician relationship.
35. In the treatment of a patient, where proven interventions do not exist or have been ineffective, the physician, after seeking expert advice, with informed consent from the patient or a legally authorized representative, may use an unproven intervention if in the physician's judgement it offers hope of saving life, re-establishing health or alleviating suffering. Where possible, this intervention should be made the object of research, designed to evaluate its safety and efficacy. In all cases, new information should be recorded and, where appropriate, made publicly available.

Statistical Analysis Plan

**Protocol No.**	**NM-V-101**
**Short Name**	--
**Title**	Double-blind, randomized, placebo-controlled study on efficacy, safety and tolerability of ancrod in patients with sudden sensorineural hearing loss (SSHL)
**EudraCT No.**	2012-000066-37
**Protocol Version**	Final version 2.0, 08-May-2012
**Protocol Amendment(s)**	Study protocol dated 19-Jul-2017 includes Amendments 1.0, 2.0, 3.1, 3.2, 4.1, 4.2, 5.0, 6.0, and 7.0
**SAP Version and Date**	**Final version 1.0**<br>**23-Aug-2018**

CONFIDENTIALITY STATEMENT

The following confidential information is the property of Nordmark Arzneimittel GmbH & Co. KG. All rights are strictly reserved. Reproductions of all or of single sections of this document are not permitted. The acceptance of this document constitutes the agreement by the recipient that no unpublished information contained within this document will be published or disclosed to third parties without prior written approval, except that this document may be disclosed to the appropriate ethics committee and regulatory authorities under the condition that they have been requested to keep this document confidential.

Dates & Signatures

By signature we declare our approval to the statistical analyses described in this SAP.

[dd-mon-yyyy]	xxx
[dd-mon-yyyy]	xxx
[dd-mon-yyyy]	xxx

Table of Contents

0. List of Abbreviations 5
1. Introduction 6
2. Changes from Protocol including Amendments 6
3. Study Overview 7
3.1. Study Objectives 7
3.2. Study Design 7
3.2.1. Target Population 8
3.2.2. Blinding 9
3.2.3. Randomization 9
3.3. Study Conduct 9
3.4. Study Variables 10
3.4.1. Primary Efficacy Variable 10
3.4.2. Secondary Efficacy Variables 10
3.4.3. Safety Variables 10
3.5. Sample Size Determination 10
4. Data Analysis Considerations 11
4.1. Analysis Populations 11
4.2. Definition of Subgroups 11
4.3. Definition of Derived Variables 12
4.4. Coding Dictionaries 14
4.5. Final Data Review and Protocol Deviations 14
4.6. General Presentation of Summaries and Analysis 15
4.7. Statistical Software 16
5. Statistical/Analytical Issues 16
5.1. General Considerations 16
5.2. Adjustments for Covariates 16
5.3. Handling of Dropouts and Missing Data 16
5.4. Interim Analysis and Data Monitoring 17
5.5. Multicenter Studies 17
5.6. Multiple Comparisons/Multiplicity 17
5.7. Examination of Subgroups 17
6. Study Population Characteristics 18
6.1. Population Overview 18
6.2. Protocol Deviations 18
6.3. Patients Excluded from Analysis Populations 19
7. Demographics and Other Baseline Characteristics 19
7.1. Demographics 19
7.2. Disease Characteristics 19
7.3. Previous and Concomitant Diseases 19
7.4. Prior and Concomitant Medications 19
7.5. Other Baseline Characteristics 20
8. Study Treatments 20
8.1. Exposure to IMP 20
8.2. Study Compliance 20
8.3. Rescue Medication 20
9. Efficacy Analyses 20
9.1. Change in PTA (Air) from Visit 1 to Day 8 (Primary Efficacy Variable) 20
9.1.1. Primary Analysis 21
9.1.2. Supportive and Sensitivity Analyses 22
9.2. Analyses of Secondary Efficacy Variables 22
9.2.1. Change in PTA (Air) from Visit 1 to Day 30 and Day 90 22

9.2.2. Change in PTA (Bone) from Visit 1 to Day 8, Day 30 and Day 90 22
9.2.3. Word Recognition Score 22
9.2.4. Fibrinogen Concentration 23
9.2.5. Biomarkers 23
9.2.6. Assessments of Hearing Impairment 23
9.2.7. Tinnitus Severity 23
9.2.8. Vertigo 24
9.3. Unplanned Analysis 24
10. Safety Analyses 24
10.1. Adverse Events 24
10.2. Death and Other Serious Adverse Events 25
10.3. Laboratory Data 25
10.4. Use in Pregnancy and Lactation 27
10.5. General Physical Examination 27
10.6. Otolaryngological Examination 27
10.7. Vital Signs 27
11. Analysis of Other Variables 28
12. References 28
13. Appendices 29
13.1. Schedule of Assessments 29
13.2. Table of 'End-of-Text' Tables / Figures 30
13.3. Table of Patient Data Listings 33
13.4. Layout of End-of-Text Tables and Listings 34
13.5. Documentation of Statistical Methods 35
13.6. Table of 'In Text' TFLs 35
13.7. Shells for TFLs 35
13.8. Raw Statistical Output 35
13.9. Data Deliverables 35

0. List of Abbreviations

AE	Adverse event
ANCOVA	Analysis of covariance
aPTT	Activated partial thromboplastin time
ATC	Anatomic Therapeutic Chemical
BDRM	Blind data review meeting
BMI	Body mass index
CD38+	Cluster of differentiation 38 or higher
CRF	Case report form
CRO	Contract research organization
CSR	Clinical study report
DMP	Data Management Plan
FAS	Full Analysis Set
FPI	First patient in
GGT	Gamma-glutamyltransferase
GOT/ASAT	Aspartate aminotransferase
GPT/ALAT	Alanine aminotransferase
HDL	High density lipoprotein
ICH	International Conference on Harmonization
IMP	Investigator manufactured product
LDH	Lactate dehydrogenase
LDL	Low density lipoprotein
LLN	Lower limit of the normal range
LOCF	Last observation carried forward
LPO	Last patient out
MedDRA	Medical Dictionary for Regulatory Activities
N/A	Not applicable
NRS	Numeric rating scale
pH	potential of hydrogen
PPS	Per Protocol Set
PTA	Pure tone audiometry/audiogram
SAP	Statistical Analysis Plan
SAS	Statistical Analysis System
SFS	Safety Set
SOC	System Organ Class
SSHL	Sudden sensorineural hearing loss
SUSAR	Suspected Unexpected Serious Adverse Reaction
tbd	to be determined
TEAE	Treatment-emergent adverse event
TFLs	Tables, Figures and Listings
TNF-α	Tumor necrosis factor alpha
ULN	Upper limit of the normal range
VLDL	Very low density lipoprotein
WHO	World Health Organization
WHO-DDE	WHO-Drug dictionary enhanced

1. Introduction

This Statistical Analysis Plan (SAP) for the study NM-V-101 is based on the final study protocol 2.0 dated 08-May-2012, its Amendment 7.0 dated 19-Jul-2017, and the corresponding CRF (version 5, dated 26-Sep-2016). The study is conducted by the sponsor Nordmark Arzneimittel GmbH & Co. KG (NORDMARK). The purpose of this SAP is to describe the statistical methods, data derivations and data summaries to be employed in this double-blind, randomized, placebo-controlled phase IIa study to assess the efficacy, safety and tolerability of ancrod in patients with unilateral idiopathic sudden sensorineural hearing loss (SSHL).

The preparation of this SAP has been based on International Conference on Harmonisation (ICH) E3 and E9 Guidelines (and references), and the statistical evaluation of all CRF parameters as well as other data sources (central safety lab data, coagulation, fibrinogen, and biomarkers including antibodies) will be performed by YYY according to this SAP.

Regarding data management and biostatistics activities, these were initially performed by the CRO XXX. With effect as of 12-Jan-2017, XXX was replaced by YYY. YYY is responsible to double-enter and clean study data as well as to perform the final statistical analysis. This includes the preparation of the Data Management Plan (DMP) and the SAP. A corresponding checklist for the SAP preparation has been agreed in advance.

This SAP is developed in a one-step procedure: After review cycles of draft versions the final SAP is generated that includes all details for the analysis with the exception of the points that are clarified at the final data review (final rules and criteria for the identification and classification of protocol deviations and possible consequences due to irresolvable queries with respect to statistical analysis). The final SAP is agreed and signed before a Blind Data Review Meeting (BDRM) takes place; all decisions made at the BDRM will be documented in BDRM meeting minutes that serve as addendum to the final SAP.

2. Changes from Protocol including Amendments

One interim analysis after 30 evaluable patients is planned in the study protocol. As it is expected that – due to expiration of available study medication – in total only a few more evaluable patients will be enrolled than planned for the interim analysis, the interim analysis is skipped. There will only be one final analysis of study data.

The study protocol defines only the Full analysis set for analysis of efficacy but requires in addition evaluability of patients in the sample size estimation. To address this, the Per protocol set is introduced in this SAP as secondary analysis population.

The study protocol states that qualitative parameters spread in very large contingency tables will be analyzed with the asymptotic chi-square-test, but as such data do not occur this test is not used in this SAP.

According to the study protocol, demographic and pre-treatment characteristics that are continuous should be analyzed by a general linear model (GLM) to determine comparability of the ancrod and placebo groups at baseline. If treatment group differences were seen at the 0.10 level of significance, these variables would be added as stratification variables or covariates to the efficacy analyses. As the study is randomized, it was decided to not follow this request and to not assess baseline differences.

The study protocol leaves open whether an analysis of covariance or a general linear model should be used to evaluate the primary efficacy variable and quantitative secondary efficacy variables. As the primary efficacy variable (change from Baseline) may be dependent on the baseline value, the baseline value should be taken into account for the primary analysis as covariate. For this reason, statistical evaluation will be performed using a generalized linear model, without prior investigation at the BDRM.

Introduced by several protocol amendments, various changes have been implemented in the CRF. However, the statistical analysis of patients being included in the study prior to an implemented CRF change is not affected, and values will be missing for added CRF parameters for all patients enrolled under a previous protocol amendment; a footnote for the respective summary tables will clarify the data issues referring to CRF amendments. In detail, the following changes relevant for the statistical analysis were made to the CRF:

- Exclusion criterion 17 was added with Amendment 4.1
- Exclusion criterion 37 was added for Czech centers only with Amendment 4.2 and for German centers with Amendment 5.0
- Exclusion criterion 14 was rephrased with Amendment 4.1
- Exclusion criteria 5 and 11 and 14 were rephrased with Amendment 5.0
- For the module "Speech intelligibility test" (Screening visit, Visit 5, Visit 6, Visit 7), an additional question was added with Amendment 4.1 ("Loudness with which 50 % of single words are understood (affected ear)")
- For the module "Vertigo" (Screening visit, Visit 5), an additional nystagmography method was added with Amendment 4.1 ("Frencl's goggles" synonymously for Frenzel goggles)
- Laboratory values were initially separated in local laboratory and central laboratory (Screening visit, Visit 5); with Amendment 4.1 they were displayed together and various lab parameters from the central laboratory were added to the CRF
- With Amendment 4.1, comment sections were added to the CRF at the end of a visit's documentation.

The database indicates what CRF version is used for a given patient.

3. Study Overview

3.1. Study Objectives

The primary objective of this study is to evaluate the efficacy of ancrod as primary treatment of SSHL compared to placebo in patients with unilateral idiopathic SSHL as determined by pure tone audiometry (PTA).

The secondary objectives of this study are to evaluate the relationship between biomarkers and clinical efficacy and to investigate the safety and tolerability of ancrod.

3.2. Study Design

This study is performed as a randomized, double-blind, multi-center, placebo-controlled, parallel-group phase II proof-of-concept study.

Study treatment and all study assessments are performed in an outpatient setting at study sites.

After obtaining informed consent, patients presenting with unilateral SSHL within 7 days after onset are screened for eligibility. Patients meeting all in- and exclusion criteria (including the screening audiometric assessments) are randomized into 2 cohorts in a ratio of 2:1 between active treatment (ancrod) and placebo. All screening assessments including randomization and patient enrollment are to be performed within 36 hours.

Immediately after randomization on Day 1 (Visit 1), patients receive the initial intravenous infusion of investigational product. The initial dose of study drug is adjusted according to the screening fibrinogen concentration by using different infusion durations at a constant infusion rate.

On the following day (Day 2, Visit 2), a venous blood sample is taken for coagulation assays. Thereafter, a weight-adjusted ancrod dose of 1 IU/kg (or placebo when randomized to placebo) is administered subcutaneously. The result of the coagulation assay is not communicated from the laboratory to the investigator or any other blinded study personnel; it is received by a dedicated unblinded person only. If the fibrinogen concentration on Day 2 is lower than 50 mg/dL, the unblinded person informs the investigator that the respective patient may not receive further injections of study drug on Day 4 and Day 6. If the fibrinogen concentration on Day 2 is ≥ 50 mg/dL, the investigator is informed by the unblinded person that the patient may receive further subcutaneous injections of 1 IU/kg ancrod (or placebo when randomized to placebo) on Day 4 and Day 6 (Visits 3 and 4).

On Day 8 (Visit 5) the treatment effect is evaluated by means of audiometry and speech recognition, and the presence and intensity of tinnitus and vertigo is determined. If no improvement of clinical signs and symptoms compared to the baseline value is achieved, the patient is allowed to switch to corticosteroid treatment, vasodilators, vitamin C, vitamin B12 or antihistamines if deemed necessary at the discretion of the investigator.

During the follow-up visits on Day 30 and Day 90 (Visits 6 and 7), the treatment effect is again determined by audiometric evaluation and tinnitus intensity to monitor for signs of relapse in the affected ear.

A schedule of assessments is given in Appendix 13.1. The study flowchart illustrates the study design:

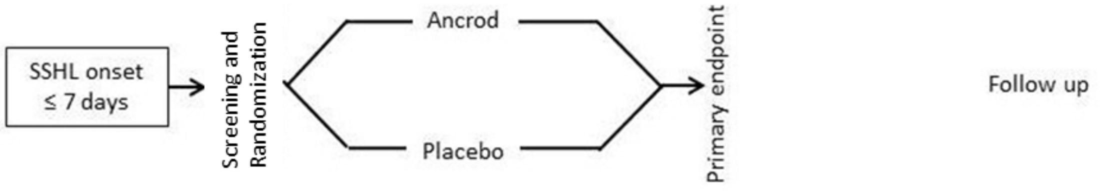

Day	1	2	3	4	5	6	7	8	30	90
Visit	1	2		3		4		5	6	7
Dosing	i.v.	s.c.		s.c.		s.c.				
Audiometry	X							X	X	X

3.2.1. Target Population

Desired study patients for enrolment are patients with unilateral SSHL as determined by audiometry.

3.2.2. Blinding

The study is carried out in a double-blind, placebo-controlled study design; neither the patient nor the investigator nor any staff collecting, reviewing or cleaning study data have knowledge of the treatment a patient receives. This applies also to any staff analyzing study data at YYY until final unblinding.

To ensure the blinding throughout the study, the following measures are taken:

- Both treatments are indistinguishable in appearance
- Appearance and labeling of vials and treatment kits do not provide any information which disclose the treatment group
- The vials and treatment kits are identified via a kit number only.

In case of medical emergency, or when information about a patient's treatment group is required in order to determine the further treatment of the patient, unblinding of individual patient's treatment can be achieved by opening the respective numbered emergency envelope. Unblinding of patients is also performed for notification of a SUSAR. Investigators have to document immediately every premature unblinding of a patient (e.g. accidental unblinding) in the CRF.

Regular unblinding for all patients including transfer of data on coagulation, biomarkers and anti-ancrod antibodies will only be done for the purpose of the final statistical analysis, after determination of the analysis populations in the BDRM and closure of the database.

3.2.3. Randomization

Treatment assignment is pre-planned according to a computer generated randomization schedule in a 2:1 (verum : placebo) ratio. Randomization is not stratified by a baseline parameter, but provides a stratification by center using the block randomization method to achieve approximately a 2:1 allocation within each center and includes the site number and patient number within site with the assigned treatment. The randomization list was generated using commercial software (RANCODE Professional, Version 3.6) by an unblinded statistician at XXX. The unblinded statistician is excluded from any other study operation. The randomization list is kept by XXX and will be provided to YYY upon request after database closure.

Each center was instructed to use the investigational products provided in ascending order, taking care of the chronological order of the patients' inclusion starting with the investigational product with the lowest number available at the site. The first study drug administration is documented in the CRF stating the precise date and time.

In the statistical analysis, treatment groups will be denoted as "Ancrod" or "Placebo", respectively.

3.3. Study Conduct

Duration of trial:

- Per patient: Screening period of up to 36 hours, then 1-week double-blind treatment phase, then 12-week follow-up phase without study treatment
- First patient in (FPI): August 2013
- Last patient out (LPO): planned for September 2018.

The study is conducted in 24 sites in Germany and Czech Republic; whereas 8 sites enrolled actively patients.

3.4. Study Variables

3.4.1. Primary Efficacy Variable

The primary outcome measure of the study is:

- Change in PTA (Air) in the affected ear from Screening (Day 1; Visit 1) until Day 8 (Visit 5). The PTA will be calculated as the arithmetic mean of air conduction thresholds at the affected consecutive frequencies in the frequency range of 0.125 to 8 kHz. A non-affected frequency within two affected frequencies will be included.

3.4.2. Secondary Efficacy Variables

Secondary outcome measures include (according to the protocol):

- Change in word recognition score from Screening to Day 8, Day 30 and Day 90
- Change in PTA (Bone) in the affected ear from Screening to Day 8, Day 30 and Day 90
- Change in PTA (Air) in the affected ear from Screening to Day 30 and Day 90
- Change in fibrinogen concentration from Screening to Day 2 and Day 8
- Change in biomarkers from Screening to Day 8
- Patient assessment of change in hearing impairment
- Physician assessment of change in hearing impairment
- Change in tinnitus severity
- Nystagmography change from Screening to Day 8.

The exact definition of the respective secondary efficacy variables is given in Section 9.2.

3.4.3. Safety Variables

Clinical safety assessments include:

- Adverse event reporting
- Change from Screening to Day 8 in general physical examination findings
- Change from Screening to Day 8 in otolaryngological examination findings
- Change from Screening to Day 8 in vital signs
- Change in laboratory parameters
- Occurrence of neutralizing anti-ancrod antibodies at Screening, Day 8, and Day 30.

3.5. Sample Size Determination

Sample size determination for this study is based on 2-sided t-tests with a global alpha level of 5 % and a statistical power of 90 %. A standard deviation of 20 dB for the test statistic is assumed. In order to be able to confirm a treatment difference of 15 dB PTA of active treatment versus placebo with a 2:1 randomization, a total number of 87 evaluable patients will be required, 58 patients in the group receiving ancrod, and 29 patients receiving placebo. To compensate for dropouts, a total number of 99 patients is planned to be enrolled, 66 patients receiving ancrod, and 33 patients receiving placebo.

4. Data Analysis Considerations

4.1. Analysis Populations

All patients having signed informed consent and provide any data in the CRF, whether receiving study treatment or not, will be considered for analysis ("*Enrolled patients*"). All patients who have been given a patient number for randomization at Visit 1 are included in the *Randomized set* independently of having received IMP. The following analysis populations that will be used for statistical evaluation will be defined:

- *Safety Set* (SFS): It includes all patients who take at least one IMP dose and for whom any safety-related data or information after the first dose of IMP are available.
  Patients will be analyzed as treated. Analysis of safety variables will be based on the SFS.
- *Full Analysis Set* (FAS): It includes all patients being randomized and receiving at least one IMP dose, regardless of any protocol deviations.
  The intention-to-treat principle is preserved despite the exclusion of patients who took no IMP, as the decision of whether or not to begin treatment could not be influenced by knowledge of the assigned treatment.
  Patients will be analyzed as randomized. The FAS is the primary population and will be used to analyze all baseline/background and efficacy variables.
- *Per Protocol Set* (PPS): It includes only patients who receive at least one IMP dose and fulfil the following evaluability criteria: have SSHL as defined per inclusion criterion 1, provide valid PTA data for Visit 1 and Visit 5, and adhere reasonably well to the study protocol without major protocol deviations (see Section 4.5).
  The PPS is a subset of the FAS and represents the secondary analysis set for baseline/ background and efficacy variables.

A definite allocation of patients to analysis sets is made at the BDRM (see Section 4.5).

4.2. Definition of Subgroups

In the study protocol, the following subgroups of patients are defined for this trial:

- Gender (male patients; female patients)
- Age of patient (below or equal to median; above median)
  Patients will be grouped for each analysis population separately
- Age of patient (4 subgroups according to the quartiles (values up to quartile))
  Patients will be grouped for each analysis population separately
- Degree of SSHL (PTA summary value at Screening >30 dB; ≤ 30 dB)
- Duration of SSHL before start of treatment (below or equal to median; above median)
  For the computation of SSHL duration, see Section 4.3; patients will be grouped for each analysis population separately
- SSHL with / without associated vestibular symptoms (spontaneous nystagmus; no spontaneous nystagmus beats) at Screening
- Presence of tinnitus (tinnitus; no tinnitus)
  If at Screening the answer to the question "Occurrence of Tinnitus?" is 'Yes', the patient will be categorized into the subgroup 'Tinnitus'
- Status of the contralateral ear at Screening (as mean of hearing loss in the frequencies 500 Hz, 1 kHz, 2 kHz, and 4 kHz):
  0 – normal hearing (25 dB or better)
  1 – mild hearing loss (>25 to 40 dB)

2 – moderate hearing loss (>40 to 60 dB)
3 – severe hearing loss (>60 to 80 dB)

- Baseline fibrinogen level (≤360 mg/dL; >360 mg/dL)
  For the computation of the baseline fibrinogen level, see Section 4.3
- Baseline fibrinogen level (4 subgroups according to the quartiles (values up to quartile))
  For the computation of the baseline fibrinogen level, see Section 4.3; patients will be grouped for each analysis population separately
- Use of rescue medication (yes; no)
  For the identification of rescue medication, see Section 8.3
- Isolated low and/or high frequency sensorineural hearing loss (PTA air):
  – With/without isolated hearing loss at the low frequencies 125 Hz, 250 Hz, or 500 Hz
  – With/without isolated hearing loss at the high frequencies 4 kHz, 6 kHz, or 8 kHz
  – With/without isolated hearing loss at the low and high frequencies.

The statistical analysis of subgroups is addressed in Section 5.7.

4.3. Definition of Derived Variables

In case of a value being not available for a target visit, imputation rules as described in Section 5.3 apply.

In this trial, the term 'baseline' is defined as the time of Visit 1 (Day 1, Screening), precisely the last available value prior to the first intake of study medication. It is expected that all data recorded for Visit 1 be obtained before application of first study treatment (exception: CRF modules "Study drug administration" and "Adverse events"). Hence, no value with date/time after start of the first study drug infusion will be regarded as baseline value; this applies also for laboratory assessments.

Patient age will be estimated as:

- Age (years) = year of Visit 1 – year of birth – 1 if month of Visit 1 is lower than month of birth
  year of Visit 1 – year of birth if month of Visit 1 is equal or higher than month of birth.

Patient's body mass index (BMI) will be computed as:

- BMI (kg/m²) = 10000 × body weight (kg) / (height (cm) × height (cm)).

For the primary efficacy variable, a complex PTA summary value will be derived. This computation is included in Section 9.1.1.

Changes from Baseline will be computed as follows:

- Absolute change (original unit) = post-baseline value – baseline value
- Relative change (%) = 100 × (post-baseline value – baseline value) / baseline value
  If the baseline value is 0, no relative change will be computed.

Relative days for study assessments will be presented in patient data listings.
Study Day 1 is the day of first IMP application as recorded in the CRF documentation for Visit 1. It should be identical with the date of Visit 1 (Screening). Relative days will be calculated as follows:

- If the (assessment/start/stop) date given is prior to Day 1, the relative day is calculated as date given minus the date of Day 1. In patient data listings, relative days based on this situation will be preceded by a '-' sign.

- If the date given is on or after Day 1, the relative day is calculated as date given minus the date of Day 1 plus 1.

The duration of SSHL will be computed as:

- Duration (days) = date of screening Visit 1 – date of SSHL + 1.

The SSHL type will be assessed by means of the following rules (to be verified at the BDRM):

- Low-tone: the two frequencies 6 kHz and 8 kHz are not affected at Screening and at least two of the three frequencies 125 Hz, 250 Hz and 500 Hz are affected at Screening
- High-tone: the two frequencies 125 Hz and 250 Hz are not affected at Screening and at least two of the three frequencies 4 kHz, 6 kHz and 8 kHz are affected at Screening
- Pantonal: 7 or all 8 frequencies are affected at Screening
- Unspecified: any other constellation of affected frequencies at Screening.

A frequency is 'affected' when the threshold value of the affected ear is at least 20 dB lower than the threshold value for the same frequency of the contralateral ear. Minimum requirement is three subsequent frequencies with at least 20 dB difference or two subsequent frequencies with at least 30 dB difference between contralateral and affected ear.

Up to now, there are two patients not meeting the affectedness criteria who however were randomized and treated. Both patients are part of the FAS, and their SSHL type is unspecified. For the evaluation of their PTA summary value, see Section 9.1.1.

- Patient 0201 has only one frequency with a threshold difference of 20 dB or more
- Patient 0302 has no frequency with a threshold difference of 20 dB or more.

Flag variables will be used in the analysis to classify below data points. They will be added to the patient data listings, if applicable:

- A flag variable will be derived to classify adverse events:
  Pre-treatment adverse events are all findings with onset before the first infusion with study drug starts.
  Treatment-emergent adverse events (TEAEs) are all findings with onset during and after first administration of the study drug until Visit 7. If an event starts on Day 1 and the onset time of the event or the start time of the infusion is missing, the event will be classified as TEAE.
  Data source for adverse events are all findings being recorded in the CRF module "Adverse Events".
- A flag variable will be derived to classify medications:
  Prior medications are all treatments where the stop date is on or before the date of Day 1.
  Concomitant medications are all treatments with either a stop date after the date of Day 1 or the answer 'No' is given as 'Date stopped'. If start date and stop date are identical and equal the date of Day 1, the treatment will only be evaluated as concomitant medication.
  Data source for prior and concomitant medications are all findings being recorded in the CRF module "Concomitant Medication" located in the CRF for each study visit.
- A flag variable will be derived to indicate the status of laboratory values:
  Conversions to the standardized unit are marked in listings by a '&' sign.
  A value *below* the lower limit of the normal range (LLN) will be indicated by a '<' mark;
  a value *above* the upper limit of the normal range (ULN) will be indicated by a '>' mark.
  Normal ranges are available only for lab values provided by the central lab (as part of the transferred data) and will be used for flag derivation; for lab values from local laboratories as entered in the CRF no normal ranges are available.

- A flag variable will be derived indicating visits or *assessments being out of the scheduled time frame* by '#'. The following time frames will be used to identify out-of-range assessments:
  - Screening (Visit 1): Start time of visit is earlier than 36 hours before the start time of first study infusion
  - Visit 2, Day 2): Not on study Day 2
  - Visit 3 (Day 4): Not on study Day 4 or 5
  - Visit 4 (Day 6): Not on study Day 6 or 7, or the day before Visit 4 is a day with IMP dose
  - Visit 5 (Day 8): Not on study Day 7 to 9, or the day of Visit 5 is a day with IMP dose
  - Visit 6 (Day 30): Earlier than Day 23 or later than Day 37
  - Visit 7 (Day 90): Earlier than Day 83 or later than Day 97.

  Deviations from the visit schedule represent a protocol deviation and are reviewed at the BDRM to determine their consequence.

4.4. Coding Dictionaries

Coding of medical terms is performed by the Data Management department of YYY as described in the DMP, using internationally recognized and accepted dictionaries.

- The coding of adverse events is done using MedDRA.
- Medications are coded using the WHO-Drug Dictionary Enhanced (WHO-DDE).

The dictionary versions finally used for analysis will be indicated in the tables and listings.

4.5. Final Data Review and Protocol Deviations

The primary purposes of a Blind Data Review Meeting (BDRM) are to discuss any statistical analysis issues and to finally review the protocol deviations detected among the randomized patients in order to classify them as minor (not relevant) or major (relevant impact on the evaluability). Each protocol deviation that may influence the results of the primary efficacy variable is rated as major. Taking the rating of protocol deviations into consideration, each patient is assigned to the corresponding analysis population (see Section 4.1).

After finishing the data cleaning process, a BDRM is arranged prior to locking the database. The conduct details of the BDRM, meeting participants and required data listings to be reviewed during the BDRM (and their suppliers) are detailed in a separate Data Review Plan.

Protocol deviations are identified during monitoring, the medical review process, the data cleaning process, or systematically by SAS programming performed by YYY. Protocol deviations are categorized with respect to: Inclusion and exclusion criteria, audiological measurements, laboratory, study drug administration, concomitant medication, visit date deviation, reporting of SAEs, GCP-relevant deviations, and other.

In addition, specific data listings are prepared for the BDRM in order to check for protocol deviations visually among the recorded medications.

Major protocol deviations include the following situations:

- Inacceptable deviation of the in- and exclusion criteria as assessed at the BDRM
- Premature unblinding of the patient's treatment allocation
- Substantial use of prohibited concomitant treatment as assessed at the BDRM
- Poor compliance to treatment or the study procedures as assessed at the BDRM
- Discontinuation of IMP due to missing fibrinogen on Day 2.

In addition, there may be other circumstances leading to exclusion from the PPS, as decided at the BDRM.

To summarize all decisions taken at the BDRM, a detailed meeting protocol will be written and signed. The BDRM meeting minutes will include a complete list of all rules and criteria on the identification and relevance assessment of actually arisen protocol deviations as agreed in the BDRM. All patients excluded from any analysis population will also be listed together with the reason for exclusion.

4.6. General Presentation of Summaries and Analysis

All study data will be presented in individual patient data listings. Data will be sorted by treatment, patient number, and visit. Where appropriate, the listings will include derived variables as per Section 4.3.

Each statistical table will contain a reference to the related patient data listing.

All study variables will first be analyzed in a descriptive way. Descriptive statistics will be displayed by treatment group (and overall if applicable) as follows:

- Continuous variables will be described using the number of non-missing values, arithmetic mean, standard deviation, minimum value, median, and maximum value ('summary statistics')
- Categorical variables will be presented by the absolute frequency (n) and the relative percentage (%) for each observed modality, whereas missing values will be displayed as own category ('frequency table').

Statistical tables will show results for each treatment group. For baseline and background variables, an additional 'Overall' column will be included, but not for efficacy and safety variables.

In general, the analysis of safety data will be based on the Safety set (SFS). The analysis of efficacy data will be based primarily on the Full analysis set (FAS), while alternative summary tables on efficacy variables will be done on the Per protocol set (PPS) as sensitivity analysis. Each analysis population will contain patients of both treatment groups in approximately a 2:1 ratio.

Usually, all patients within a given analysis population will be considered for analysis; descriptive by-visit analyses will be based upon only those patients who did not terminate the study prior to a given visit. Each table will display the total number of patients per treatment group (and overall, if applicable).

Further specifications regarding the layout of end-of-text tables and listings are given in Appendix 13.4 of this SAP. A list of all statistical tables and listings to be produced can be found in Appendices 13.2 and 13.3. These overviews also indicate the analysis population(s) for which a respective parameter will be evaluated. Moreover, the generation of mock TFLs is addressed in Section 13.7.

Any figures/graphics produced must be legible after been copied in black even if the original version is colored.

The layout of in-text tables for a subsequent report is covered in Appendix 13.6.
Raw SAS procedural outputs will be handed over to NORDMARK for the primary efficacy variable, as stated in Appendix 13.8.

4.7. Statistical Software

All statistical analyses will be performed by programming using the software SAS® Version 9.3 or higher. (STATISTICAL ANALYSIS SYSTEM, SAS Institute, Cary, NC, USA).

5. Statistical/Analytical Issues

5.1. General Considerations

Based on the raw database containing the recorded CRF data and on other data such as protocol deviations and central lab data, analysis data sets according to internal standards will be programmed that will be used for the generation of summary tables, figures and listings (TFLs). To meet common quality control requirements, the analysis of the following variables will be double-programmed by a second biostatistician:

- Change in PTA (Air) in the affected ear from Visit 1 to Visit 5 (including the corresponding analysis data set needed for the primary efficacy variable)
- Adverse event rates.

For all other TFLs, a second biostatistician will review the SAS program codes and will check the results for consistency at least visually. All SAS programs used for the evaluation will be stored in a study-specific directory on the YYY file server together with the corresponding 'list' and 'log' files.

Quantitative efficacy variables (including the primary efficacy variable) will be analyzed by means of an analysis of covariance (ANCOVA) based on the generalized linear model (SAS procedure PROC GLM). Underlying model assumptions, such as normality and homogeneity of variance, will be assessed at the BDRM. Should these assumptions be violated, appropriate data transformations or the choice of a non-parametric analysis method will be considered. These analyses will be performed for the primary efficacy variable in addition to the originally planned analysis.

For the primary efficacy variable, confirmative testing will be performed with the aim to show superiority in favor of the treatment with ancrod over placebo. Basically, a two-sided 5 % significance level will apply. All other variables will be exploratively compared between treatment groups.

5.2. Adjustments for Covariates

Besides the treatment group as fixed effect, the following covariates will be included in the ANCOVA models for quantitative efficacy variables:

- Study center (random effect)
- Baseline value (covariate).

The study center is employed in the statistical model to compensate for site effects leading to a high variance in endpoint values among centers, and the baseline value is included due to possible disease severity among patients.

5.3. Handling of Dropouts and Missing Data

A screening failure is a patient having signed informed consent but discontinuing before being randomized. Such patients are not kept in the database.

Dropout patients are not explicitly referenced in the study protocol. However, the following definition is provided in this SAP:

- Patients randomized who either discontinue the study prematurely or are withdrawn from study by the investigator before the planned study termination are assessed as **dropouts**.

Dropout patients are not substituted. To compensate for dropouts, a 12 % dropout rate was used in the sample size determination.

Dropout patients will be evaluated like any other patient terminating the study regularly while accounting for missing data. The FAS includes dropout patients.

Missing values will usually be shown as missing in patient data listings. If there is the need to show data imputations, imputed values are flagged appropriately.

For the statistical analysis of efficacy variables, the LOCF (last observation carried forward) method will be applied to impute missing Visit 5 values, i.e, the baseline value will be carried forward. Missing values for Visit 6 or Visit 7 will not be replaced as these visits are far beyond treatment stop.

Regarding PTA, a missing PTA threshold value for a given frequency at Visit 5 is replaced by the respective preceding value available at Screening. Imputations will not use values of the other ear (left/right) or of the other conduction type (air/bone). If a baseline value for a given frequency is missing, the frequency will be excluded from statistical analysis of the respective patient.

Regarding the statistical evaluation of background and safety variables, missing values will not be replaced or imputed; they will be handled as missing by displaying an own category in frequency tables.

However, missing data, outliers and other spurious or implausible data are discussed in the BDRM prior to unblinding the database.

5.4. Interim Analysis and Data Monitoring

No data monitoring committee is established for this trial.

Although introduced by a protocol amendment, an interim analysis will not be performed.

5.5. Multicenter Studies

This study is conducted as a multicenter trial. The data of all centers will be combined for statistical analysis.

The study center will be used as covariate for statistical testing, see Section 5.2. In case of a large variability of enrolment at centers, study centers will be pooled according to external criteria (e.g., country, region, or investigator's experience). The exact pooling rule is established at the BDRM, before unblinding.

5.6. Multiple Comparisons/Multiplicity

As confirmative statistical testing will be done for the primary efficacy variable only, no p-value adjustments for multiple comparisons nor power adjustments are necessary. All p-values gained underlie the experiment-wise significance level without adjustment for a study-wide level.

5.7. Examination of Subgroups

The patient subgroups as defined in Section 4.2 will be evaluated descriptively for the following study variable:

- Change in PTA (Air) in the affected ear from Visit 1 to Visit 5 (see Section 9.1.1).

In addition, to assess the relevant risk factors, a step-down analysis of covariance (ANCOVA) model will be established on above study variable that initially includes the subgroups of Section

4.2 (but not subgroups based on quartiles and the subgroups for rescue medication use) and the following factors at Visit 1:

- Biomarkers (CD38+ and CD42+ cells, interleukin-6, TNF-α)
- Frequencies affected by SSHL (high-tone, low-tone, pantonal, unspecified)
- Diabetes mellitus (yes, no)
- Hypertension (yes, no)
- Ischemic heart disease (yes, no).

Diabetes mellitus, hypertension and ischemic heart disease will be identified during the BDRM based on the entries for physical examination at Visit 1.

In a first step, all variables with low influence ($p>0.5$) will be removed. From the resulting model, successively the factor with lowest influence will be eliminated until a model remains that contains only the significant influence factors ($p<0.05$).

6. Study Population Characteristics

All statistical tables described in this chapter will show aggregated data by treatment group and overall.

6.1. Population Overview

Frequency tables will be prepared presenting the number of patients randomized, eligible (i.e., meeting all inclusion criteria but none of the exclusion criteria), and treated. Moreover, the following information will be given for all patients randomized:

- The date of the FPI (date of informed consent)
- The date of first patient's first IMP intake
- The date of last patient's last treatment
- The date of the LPO (date of last documented visit).

Reasons for exclusion from any analysis population will be shown in a patient data listing.

For all patients randomized, the following information will be summarized in a frequency table:

- The number and percentage of patients completing the trial [1] and of those who terminated the trial prematurely ('Dropouts')
- The number of patients with unblinding performed
- The number of patients per study center
- The patient disposition by visit (attended/missed).

[1] 'Completers' are patients with the answer 'Yes' to the question 'Regular end of observation?' on the CRF's End of Study form.

The reason for leaving the study (applies to dropouts only) will be given in a patient data listing.

6.2. Protocol Deviations

The number of patients who are member of the SFS, FAS and PPS will be included in a frequency table together with the number and percentage of all randomized patients with

- at least one deviation from the study protocol
- at least one major deviation

- at least one minor deviation but no major deviation.

In addition, the number and percentage of all randomized patients with major deviations will be summarized by type of deviation. Correspondingly, the number and percentage of all randomized patients with minor deviations will be summarized by type of deviation.

A patient data listing will be prepared displaying each randomized patient's protocol deviations.

6.3. Patients Excluded from Analysis Populations

The BDRM meeting minutes depict all patients excluded from any analysis set as determined during the BDRM. A patient data listing will be prepared displaying for patients excluded the reason(s) for exclusion.

7. Demographics and Other Baseline Characteristics

Demographic characteristics and other baseline/background data as listed below will be evaluated descriptively for the SFS, FAS, and PPS; all results will be given by treatment group and overall.

7.1. Demographics

Summary statistics or frequency tables will be computed for age, sex, and BMI. Age will be tabulated in addition with the categories <18 years, 18 to 30 years, >30 to 50 years, >50 to 70 years, and >70 years.

7.2. Disease Characteristics

The following data from the Screening visit will be summarized to describe the underlying study conditions:

- Duration of SSHL (days) – summary statistics
- Affected ear (right ear, left ear) – frequency table
- Occurrence of tinnitus (yes, no) – frequency table.

All other disease characteristics recorded in the CRF will be included in the evaluation of efficacy variables.

7.3. Previous and Concomitant Diseases

N/A – diseases are not captured explicitly in the CRF.
Findings of the general physical examinations include diseases and are analyzed as described in Section 10.5.

7.4. Prior and Concomitant Medications

For the definition of prior and concomitant medications, refer to Section 4.3. Recorded treatments are coded by medication groups as described in the DMP. The allocation of reported terms to higher-level classifications is made using the preferred ATC code.

Prior and concomitant medications will be summarized separately using Level 2 (3-digit) and Level 3 (4-digit) of the ATC code. Frequency tables will be generated on a patient basis: Patients are counted only once for each ATC class when they have more than one medication within the same ATC class. Patients with more than one medication within a level 2 ATC class will be counted for each corresponding level 3 term, but only once for the level 2 class. The number and percentage of patients with at least one medication reported will also be displayed. All tabulations will be sorted alphabetically by level 2 ATC decode and by frequency of level 3 decodes within level 2 class.

Specific findings on medications will be presented in a corresponding patient data listing.

7.5. Other Baseline Characteristics

For female patients, birth control data from Visit 1 (pregnancy test performed yes/no and result) will be listed only.

8. Study Treatments

8.1. Exposure to IMP

To describe IMP exposure during study, the following summary tables will be presented by treatment group for the SFS, FAS, and PPS:

- Number of study drug administrations (intravenously and subcutaneously separately)
- Total amount of IMP given (I.U. by administration and overall).

8.2. Study Compliance

N/A – as the study treatment is applied via infusion or injection at the site, consideration of compliance will not be meaningful.

8.3. Rescue Medication

Corticosteroids, vasodilators, vitamin C, vitamin B12 or antihistamines are allowed if deemed necessary at the discretion of the investigator, if, on Day 8, no improvement or worsening of symptoms has been observed. Rescue medication among the concomitant medications will be identified in the BDRM and will be analyzed in analogy to the evaluation of concomitant medications presented in Section 7.4.

9. Efficacy Analyses

The primary analysis of all efficacy variables will be based on the Full analysis set (FAS). For the following efficacy variables, the evaluation will be repeated for the PPS as sensitivity analysis:

- Change in PTA (Air) in the affected ear from Screening to Day 8 (primary efficacy variable)
- Change in PTA (Bone) in the affected ear from Screening to Day 8, Day 30 and Day 90
- Change in PTA (Air) in the affected ear from Screening to Day 30 and Day 90
- Change in word recognition score from Screening to Day 8, Day 30 and Day 90.

Besides descriptive analyses, inferential statistics will be computed that are confirmatory for the primary efficacy variables and of explorative nature only for all secondary variables. All statistical tables will show data aggregations by treatment group but not for overall.

9.1. Change in PTA (Air) from Visit 1 to Day 8 (Primary Efficacy Variable)

A pure tone audiogram (PTA) is conducted for each ear separately at Screening (Visit 1), Day 8 (Visit 5), Day 30 (Visit 6), and Day 90 (Visit 7). Auditory thresholds for the methods 'PTA Air' and 'PTA Bone' are recorded in the CRF for the frequencies 0.125, 0.25, 0.5, 1, 2, 4, 6, and 8 kHz.

In a first step, the audiograms (right ear, left ear) for air conduction at Screening will be compared to identify the ear affected by SSHL and the affected frequencies (a decrease of ≥20 dB in the affected ear compared to the contralateral ear).

9.1.1. Primary Analysis

Primary efficacy variable is the (absolute) change in PTA (Air) in the affected ear from Visit 1 to Visit 5. The PTA summary value for a given visit will be calculated as the arithmetic mean of air conduction thresholds at the affected consecutive frequencies in the frequency range of 0.125 to 8 kHz. One non-affected frequency within two affected frequencies will be included in the PTA summary value; two or more non-affected frequencies will not be taken into account.

Patient 1303 has an unspecified SSHL type. While only the four high-tone frequencies 2 kHz to 8 kHz meet the criteria for affectedness, also the two low-tone frequencies 125 Hz and 250 Hz with differences of 25 or 20 dB will be included to compute the PTA summary value. The two non-affected frequencies 500 Hz and 1 kHz will be omitted.

Patient 0201 has only one frequency with a threshold difference of 20 dB or more. The PTA summary value will be based on all eight frequencies.

Patient 0302 has no frequency with a threshold difference of 20 dB or more. The PTA summary value will be based on all eight frequencies.

For post-baseline visits, the PTA summary value will be based on those frequencies identified as affected at Screening. If one or more threshold values for a frequency are missing at Visit 5, LOCF imputation routines as described in Section 5.3 apply. In case a frequency shows a difference of 20 dB or more at a post-baseline visit but the frequency was not affected at Screening, the frequency will not be taken into account for any PTA summary value.

For the FAS, the PTA summary values by visit and the absolute and relative change in PTA (Air) in the affected ear from Visit 1 to Visit 5 will be presented by treatment group. PTA values over time will additionally be presented by means of a boxplot.

The primary statistical analysis will be performed with the aim to show superiority of ancrod over placebo. The following two-sided study hypothesis at the 5% significance level will be evaluated:

H_0: $Chg_{Ancrod} = Chg_{Placebo}$ vs
H_1: $Chg_{Ancrod} \neq CCR_{Placebo}$ where Chg denotes the absolute change in PTA.

A general linear model (GLM) with treatment as fixed effect, center (after applied pooling) as random effect, and baseline PTA as covariate will be calculated. The resulting two-sided p-values will be displayed. The model is:

Change in PTA = treatment + center + PTA at Visit 1.

Underlying assumptions, such as normality and homogeneity of variance, are assessed at the BDRM. Should these assumptions be violated, appropriate data transformations or the choice of a non-parametric analysis method will be considered.

In addition, the mean change at the affected frequencies will be classified into the following categories and evaluated in a frequency table using the Freeman-Halton test:

Definition*	Improvement
Recovery	Hearing level recovers within 30 dB at 250, 500, 1000, 2000 Hz and within 25 dB at 4000 Hz, or<br>Hearing level recovers to that of contralateral intact ear if hearing in intact ear can be considered to be stable**
Good	Average hearing improvement for the five frequencies ≥ 30 dB

Fair	Average hearing improvement for the five frequencies ≥ 10 and ≤ 30 dB
No change	Average hearing improvement for the five frequencies is within 10 dB

* Hearing recovery as defined by the Ad Hoc Committee of the Ministry of Health and Welfare in Japan
** The contralateral intact ear is regarded as stable when threshold values for Visit 1 and Visit 5 differ at most by 10 dB.

Besides, a comparison of mean changes of participants who completed the study will be made: Multiple linear regression analysis will be performed to evaluate the magnitude of the treatment effects after adjustment for participant characteristics such as age, duration of SSHL prior to treatment, and baseline degree of hearing loss (mild ≤ 30 dB; moderate to profound > 30 dB).

Moreover, for the subgroups defined in Section 4.2, the mean changes in PTA will be analyzed for the FAS as described in Section 5.7.

9.1.2. Supportive and Sensitivity Analyses

As a sensitivity analysis, the statistical evaluation as shown in Section 9.1.1 will be repeated for the PPS.

9.2. Analyses of Secondary Efficacy Variables

For secondary efficacy variables (except for fibrinogen) also, LOCF imputation will be done for missing post-baseline values up to Visit 5.

9.2.1. Change in PTA (Air) from Visit 1 to Day 30 and Day 90

The change in PTA (Air) in the affected ear from Visit 1 to Day 30 (Visit 6) and Day 90 (Visit 7) is a secondary efficacy variable. The statistical analysis will be conducted for the FAS and the PPS in analogy to the primary efficacy variable, excluding the categorical analysis, the multiple linear regression, and the subgroup analyses.

9.2.2. Change in PTA (Bone) from Visit 1 to Day 8, Day 30 and Day 90

For PTA (Bone), the definition of the affected frequencies and the computation of the PTA summary value per visit will be based on the bone-conducted thresholds in the same manner as described above for PTA (Air). The statistical analysis of the changes in PTA (Bone) will be conducted for the FAS and the PPS in analogy to the primary efficacy variable, excluding the categorical analysis, the multiple linear regression, and the subgroup analyses.

9.2.3. Word Recognition Score

A speech intelligibility test is conducted at Screening (Visit 1), Day 8 (Visit 5), Day 30 (Visit 6), and Day 90 (Visit 7). Country specific test results are given as percentage or dB value of the following four items:

- Percentage of words understood in affected ear out of 40 words (at a loudness of 60 dB)
- Percentage of words understood in affected ear out of 40 words (at a loudness of 80 dB)
- Loudness (dB) with which 50 % of digits are understood in affected ear
- Loudness (dB) with which 50 % of single words are understood in affected ear.

The change in word recognition from Screening to Day 8, Day 30 and Day 90 will be analyzed for the FAS and the PPS in analogy to the primary efficacy variable separately by country, excluding the categorical analysis, the multiple linear regression, and the subgroup analyses.

9.2.4. Fibrinogen Concentration

The blood coagulation parameter fibrinogen is determined at Screening (Visit 1), Day 2 (Visit 2), and Day 8 (Visit 5). Fibrinogen concentrations at Screening and Day 2 influence the duration of infusion and the dosage of study medication.

The change in fibrinogen level from Screening to Day 2 and Day 8 will be analyzed for the FAS in analogy to the primary efficacy variable, excluding the categorical analysis, the multiple linear regression, and the subgroup analyses. If a value is available twice (in the CRF and in the central lab data), both will be listed but only the CRF value will be used for statistical analysis.

9.2.5. Biomarkers

The following biomarkers are determined at Screening (Visit 1) and Day 8 (Visit 5) in venous blood samples as surrogate parameters for efficacy:

- Inflammatory markers: interleukin-6, TNF-α
- Molecular markers of leucocyte activation: CD38+, CD40+ cells.

The change in biomarkers from Screening to Day 8 will be analyzed for the FAS in analogy to the primary efficacy variable, excluding the categorical analysis, the multiple linear regression, and the subgroup analyses.

Moreover, the inflammatory and cellular markers' relative change at Visit 5 will be correlated (Spearman's correlation coefficient) with the following effects:

- PTA (Air) absolute change at Visit 5
- Fibrinogen relative change at Visit 5.

9.2.6. Assessments of Hearing Impairment

During the otolaryngological examination at Screening (Visit 1), Day 8 (Visit 5), Day 30 (Visit 6), and Day 90 (Visit 7), the patients and the investigators are asked to estimate the current hearing loss by means of a numeric rating scale (NRS) with 11 items ranging from 0 to 10 points. It is understood that 0 refers to 'no hearing loss' and 10 to 'complete hearing loss'.

Separately for the patient assessment and physician assessment, the change in NRS values from Screening to Day 8, Day 30 and Day 90 will be analyzed for the FAS in analogy to the primary efficacy variable, excluding the categorical analysis, the multiple linear regression, and the subgroup analyses.

In addition, for each visit, NRS values will be transformed to three qualitative categories:

- Mild hearing loss (NRS value of 0, 1, 2, or 3)
- Moderate hearing loss (NRS value of 4, 5, or 6)
- Severe hearing loss (NRS value of 7, 8, 9, or 10).

For each visit, the absolute and relative frequency of patients within each category will be presented by treatment group. For Day 8, Day 30 and Day 50, the 3×2 contingency tables will be analyzed with the Freeman-Halton test.

9.2.7. Tinnitus Severity

Tinnitus is assessed at Screening (Visit 1), Day 8 (Visit 5), Day 30 (Visit 6), and Day 90 (Visit 7). Patients rate the current tinnitus severity (ringing) by means of a numeric rating scale (NRS) with 11 items ranging from 0 to 10 points. It is understood that 0 refers to 'no tinnitus' and 10 to 'worst

tinnitus'. If the NRS is not completed and the answer 'no' to the question 'Occurrence of Tinnitus?' is provided, the NRS value 0 will be used for analysis.

The change in tinnitus severity from Screening to Day 8, Day 30 and Day 90 will be analyzed for the FAS in analogy to the primary efficacy variable, excluding the categorical analysis, the multiple linear regression, and the subgroup analyses.

9.2.8. Vertigo

Data on vertigo (method of nystagmography, number of spontaneous nystagmus beats per minute) are collected at Visit 1 and Visit 5 and will be presented in patient data listings.

9.3. Unplanned Analysis

All analyses described above are included in the study protocol with the exception of the introduction of the Per protocol set and additional subgroup analyses (use of rescue medication, isolated low and/or high frequency sensorineural hearing loss).

10. Safety Analyses

All safety parameters will be evaluated for all patients treated (Safety set, SFS). All statistical tables will show data by treatment group but not for overall. If not indicated otherwise, only descriptive statistics will apply.

10.1. Adverse Events

All adverse events (AEs) as recorded in the CRF module "Adverse Events" will be analyzed. Initially, an overview will be generated tabulating the number and percentage of patients (and the total number of events) with

- adverse events
- treatment-emergent AEs (for definition, see Section 4.3)
- treatment-emergent AEs with causal relationship (to IMP) given as 'Yes', or missing
  Note: When the answer to causality is not specified the AE will be analyzed as IMP-related
- treatment-emergent AEs leading to discontinuation ('Measures' is 'Treatment stopped')
- serious treatment-emergent AEs
- serious treatment-emergent AEs with causal relationship ('Causality' as above)
- serious treatment-emergent AEs leading to discontinuation ('Measures' as above).

For each category, AE rates of both treatment groups will be compared by Fisher's exact test.

Adverse events are coded according to the MedDRA terminology as described in the DMP. The following summaries for adverse events will be provided broken down by System Organ Class (SOC) and Preferred Term within SOC, separately for each treatment group:

- All treatment-emergent AEs (TEAEs)
- All TEAEs by maximum intensity ('Mild', 'Moderate', 'Severe', 'Life threatening', 'Death')
- All TEAEs related to IMP ('Causality' is given as 'Yes' or missing)
- All TEAEs leading to discontinuation ('Measures' is 'Treatment stopped')
- All TEAEs by action taken ('None', 'Treatment stopped', 'Dose reduced', 'Other treatment', 'Hospitalization') [1]
- All TEAEs by outcome ('Disappeared', 'Ongoing', 'Sequelae', 'Unknown', 'Death')
- All serious TEAEs

- All serious TEAEs related to IMP (causality as above)
- All serious TEAEs leading to discontinuation (measures as above).

[1] In case multiple responses per event are given, the event is counted for each response.

These summaries will generally provide the number and percentage of patients with TEAEs (and the total number of events) within each preferred term and within each SOC. Patients are counted only once for each preferred term when they have more than one AE with the same preferred term. Patients with more than one AE within a SOC will be counted for each corresponding preferred term within the SOC, but are counted only once for the SOC. The number of patients with at least one TEAE and the total number of events will also be displayed.

All tabulations will be sorted alphabetically by SOC and by frequency of preferred terms within each SOC.

A glossary of adverse events will be presented showing how investigator reported terms are actually coded to preferred terms and body systems.

All adverse events and associated collected information (including reported term, start and stop date/time, relative days and duration, treatment applied, seriousness, intensity, causality, measures taken, and outcome) will be presented by patient in a corresponding patient data listing.

10.2. Death and Other Serious Adverse Events

In addition to the general AE listing, a separate patient listing will be generated for serious adverse events and death cases. Summary tables on serious TEAEs are described in Section 10.1.

10.3. Laboratory Data

Data on laboratory parameters are provided in the CRF or electronically by the central lab. They refer to blood coagulation, clinical chemistry, hematology, biomarkers, antibodies, and urinalysis; relevant for safety purposes are all except biomarkers. If a value is available twice (in the CRF and in the central lab data), both will be listed but only the CRF value will be used for statistical analysis.

All results of laboratory tests will be listed, including reference ranges (available only for central lab data) and possible details on conversion to the standardized units shown in below table. Unusual lab values are reviewed at the BDRM. Values recorded as lower or upper border only will be evaluated as the border (e.g. >140 as 140); however, if all values of a parameter are recorded as border value, the analysis will only be categorical.

The laboratory parameters will be displayed in the analysis in the order as shown in Table 2:

TABLE 2: LABORATORY PARAMETERS

Group	Parameter (standardized unit)
Hema-tology	Hemoglobin (g/dL)
	Hematocrit (%)
	Erythrocytes (10^6/µL)
	Leukocytes (10^3/µL)
	Thrombocytes (10^3/µL)
	Mean cell volume (µm³)

Group	Parameter (standardized unit)
	ESR (mm/h)
Blood chemistry	Creatinine (µmol/L)
	GOT/ASAT (U/L)
	GPT/ALAT (U/L)
	Sodium (mmol/L)
	Potassium (mmol/L)
	Chloride (mmol/L)
	Glucose (mmol/L)
	Urea (mmol/L)
	Uric acid (µmol/L)
	Phosphorus (mmol/L)
	Total bilirubin (µmol/L)
	Total protein (g/L)
	Albumin (g/L)
	LDH (U/L)
	GGT (U/L)
	Alkaline phosphatase (U/L)
	Creatinine phosphokinase (µkat/L)
	Triglycerides (mmol/L)
	Cholesterol (mmol/L)
	HDL (mmol/L)
	LDL (mmol/L)
	VLDL (mmol/L)
Coagulation #	Thrombin time (sec)
	Prothrombin time (INR)
	aPTT (sec)
Urinalysis	Hemoglobin or blood in urine * °
	pH (no unit)
	Specific gravity (no unit)
	Protein (mg/L)
	Glucose (mmol/L)
	Ketones *
	Bilirubin *
	Leukocytes *
Antibodies	Neutralizing anti-ancrod antibodies *

# The coagulation parameter fibrinogen is analyzed as efficacy variable.
* Categorical analysis with 'Positive and 'Negative'.
° Before CRF version 4.1, the parameter titled "Blood in urine".

For each numeric parameter, the baseline value will be determined as the latest value before or at Day 1. Then, values at Baseline and Day 8 as well as the absolute change will be given in a summary table by treatment group.

Moreover, a shift analysis will be presented for each numeric parameter. In this, the categorized change from Baseline to Day 8 will be displayed. Possible categories are 'Below the LLN', 'Within the normal range', and 'Above the ULN', according to the flag variable introduced in Section 4.3.

Categorical parameters will be presented in frequency tables by visit (Baseline, Day 8).

The number of patients with clinically relevant laboratory values will be given by parameter in a frequency table.

10.4. Use in Pregnancy and Lactation

The use of IMP during pregnancy or breastfeeding is not allowed. Hence, only male patients or females being in non-childbearing age, performing birth control or showing a negative pregnancy test result are to be included. To document birth control, a urine or serum pregnancy test is performed at Screening (Visit 1) for each female patient of childbearing potential and premenarches. Pregnancy test data will be presented in a patient data listing.

10.5. General Physical Examination

A physical examination is performed at Screening (Visit 1) to document the patient's medical history and at Day 8 (Visit 5) to document changes since Screening.

Separately for each body system listed in the CRF, the categories at Screening ('Not done', 'Normal', 'Abnormal') and at Day 8 ('Not done', 'No change', 'Change') will be given in a frequency table. Any specifications for abnormality and change will be presented in a patient data listing.

10.6. Otolaryngological Examination

An otolaryngological examination is performed at Screening (Visit 1), Day 8 (Visit 5), Day 30 (Visit 6), and Day 90 (Visit 7). While the assessments to estimate hearing loss are evaluated for efficacy, the following items collected at Screening and Day 8 represent safety parameters:

- Ear microscopy results (affected ear)
- Tympanometry results (affected ear)
- Stapedius reflex measurements results (affected ear)
- Otoacoustic emissions examination results (affected ear)

Separately for each item, the categories 'Normal' and 'Finding' will be given in a frequency table by visit. Any specifications on findings will be presented in a patient data listing.

10.7. Vital Signs

Vital sign parameters (body weight, systolic and diastolic blood pressure, and pulse rate) are recorded for Screening (Visit 1) and Day 8 (Visit 5). All data will be listed. Values of above four parameters for Screening and Day 8 and the absolute change from Screening to Day 8 will be displayed in a summary table.

In addition, a frequency table will be provided for pulse pattern ('Regular', 'Not regular') by visit.

Vital signs will also be tabulated in shift tables providing the number of patients whose values are within or outside the normal ranges. The following normal ranges (orientating at WHO definitions) will be used:

- BMI: 18.5 to <25.0 kg/m²
- Systolic blood pressure: 105 to <140 mmHg
- Diastolic blood pressure: 65 to <90 mmHg
- Pulse rate: 60 to <85 beats per minute.

11. Analysis of Other Variables

N/A.

12. References

ICH E3, Structure and Content of Clinical Study Reports, 1996

ICH E9, Statistical principles for clinical trials, 1988

SAS, Statistical Analysis System, SAS Institute, Cary, NC, USA

Study protocol for study NM-V-101, final version including Amendment 7.0, dated 19-Jul-2017, with associated CRF

Data Management Plan for study NM-V-101, version 1.0, dated 05-Jul-2017

Data Review Plan for study NM-V-101, version 1.0, dated 04-Apr-2018.

13. Appendices

13.1. Schedule of Assessments

TABLE 1: SUMMARY OF STUDY VISITS AND PROCEDURES

	**Scree-ning**	**Rando-miza-tion**	**Start treat-ment**	**Double-blind Treatment Phase**				**Follow-up**	
**Day**	**1 (may take 36 hours)**			**2**	**4 (+1)**	**6 (+1)**	**8 (±1)**	**30 (±7)**	**90 (±7)**
**Visit**	**1**			**2**	**3**	**4[a]**	**5[a]**	**6**	**7**
Informed consent	✓								
Inclusion/exclusion criteria	✓								
Demographics, baseline characteristics	✓								
Pregnancy test[b]	✓								
Medical history	✓								
Previous medication[c]	✓								
Physical and otolaryngo-logical examination[d]	✓						✓		
Coagulation[e]	✓*			✓ **f			✓[f]		
Clinical Chemistry[g]	✓						✓		
Hematology[h]	✓						✓		
Urinalysis[i]	✓						✓		
Audiometric evaluation[j]	✓						✓	✓	✓
Randomization		✓							
Study drug administration			✓	✓	✓[k]	✓[k]			
Concomitant medication	✓			✓	✓	✓	✓	✓	✓
Biomarker[l]	✓**						✓**		
Neutralizing anti-ancrod antibodies[m]	✓**						✓**	✓**	
Vital signs	✓						✓		
Nystagmography / Frenzel goggles	✓						✓		
Tinnitus	✓						✓	✓	✓
NRS Patient/Physician assessment	✓						✓	✓	✓
Adverse events[n]			✓	✓	✓	✓	✓	✓	✓

a: Visit 4 and 5 are to be performed on different days.

b: Performed in premenopausal women who have not been surgically sterilized (documented complete hysterectomy or bi-tubal ligations) or post-menopausal >1 year. Analysis in urine or serum, study drug may not be administered before obtaining pregnancy test results.

c: All medication taken within the last 30 days before Visit 1 should be documented.

d: Physical examination including general appearance (including lymphatic nodes), HEENT (head, eyes, ears, nose and throat), cardiovascular, respiratory, gastrointestinal/abdomen, musculoskeletal, skin/dermatological, neurologic and other examinations.

Otolaryngological examination including otoacoustic emissions, tympanometry, stapedius reflex measurement and ear microscopy.

e: Parameters to be determined in the selected laboratory (local or central) of the site for safety reasons: Fibrinogen, Thrombin time, Prothrombin time, aPTT.

*The initial dose of study drug will be adjusted according to the screening fibrinogen concentration assessed in the local laboratory.

**The assay on Day 2 will be performed within 36 hours in the selected laboratory of the site.

f: The investigator must announce the taking of the blood sample on Day 2 for fibrinogen one day before per fax to ProjectPharm and if necessary to the courier company. ProjectPharm has to be immediately informed in writing (fax) from the responsible laboratory or designee on the result of the coagulation assays on Days 2 and 8. The result of the coagulation assay may in principle not be communicated from the laboratory to the investigator or any other blinded study personnel.

g: Responsible laboratory: Creatinine, aspartate aminotransferase (SGOT; AST), alanine aminotransferase (SGPT; ALT), sodium, potassium, chloride, glucose, urea, uric acid, phosphorus, total bilirubin, total protein, albumin, lactate dehydrogenase (LDH), gamma-glutamyltransferase (γ-GT), alkaline phosphatase, creatinine phosphokinase, triglyceride, cholesterol, high density lipoprotein (HDL), low density lipoprotein (LDL), and very low density lipoprotein (VLDL).

h: Responsible laboratory: Erythrocytes, hematocrit, MCV, leucocytes, erythrocyte sedimentation rate (ESR), hemoglobin, thrombocytes.

i: Responsible laboratory: Hemoglobin or blood in urine, pH, specific gravity, protein, glucose, ketones, bilirubin, leucocytes.

j: Air/bone pure tone audiogram (PTA) and speech intelligibility test inclusive word recognition.

k: Study drug on Days 4 and 6 may only be administered after the investigator has been informed by ProjectPharm that the fibrinogen concentration on Day 2 is ≥ 50 mg/dl.

l: **Central laboratory.

m: **Central laboratory.

n: If an AE is reported as ongoing at Day 90, an additional follow-up will be performed four weeks (± 1 week) later by telephone. If the ongoing AE at Day 90 involves a laboratory abnormality, an extra visit will occur four weeks (± 1 week) later for assessment of laboratory results.

13.2. Table of 'End-of-Text' Tables / Figures

[To be included in Appendix 14.1-3.x of the CSR]
The numbering of statistical tables follows the ICH E3 guideline. Shown abbreviations denote the analysis populations for which a variable will be evaluated. Please note that some tables may be split into subtables covering different aspects of the evaluation. Besides, an additional number for analysis population will be attached to the shown table/figure number.

Number	Title	Analysis Population
Table 14.1.1	Patient Disposition	Randomized set
Table 14.1.2	Attendance of Study Visits	Randomized set
Table 14.1.3	Patients by Study Center	Randomized set
Table 14.1.4	Summary of Patient Populations and Protocol Deviations	Randomized set
Table 14.1.5	Demographic Data	SFS, FAS, PPS
Table 14.1.6	Disease Characteristics	SFS, FAS, PPS
Table 14.1.7.1	Prior Medication	SFS, FAS, PPS
Table 14.1.7.2	Concomitant Medication	SFS, FAS, PPS
Table 14.1.7.3	Rescue Medication	SFS, FAS, PPS
Table 14.1.8	Exposure to IMP	SFS, FAS, PPS

Number	Title	Analysis Population
Table 14.2.1.1	Pure Tone Audiogram (PTA Air) Values over Time	FAS, PPS
Figure 14.2.1.1	Boxplots of Pure Tone Audiogram (PTA Air) Values	FAS, PPS
Table 14.2.1.2	Change in PTA Air from Screening to Day 8	FAS, PPS
Table 14.2.1.3	Categorical Changes in PTA Air from Screening to Day 8	FAS, PPS
Table 14.2.1.4	Multiple Linear Regression on Change in PTA Air from Screening to Day 8	FAS, PPS
Table 14.2.1.5	Change in PTA Air from Screening to Day 8 by Subgroups	FAS
Table 14.2.1.6	Step-Down ANCOVA on Change in PTA Air from Screening to Day 8 to Identify Risk Factors	FAS
Table 14.2.2.1	Change in PTA Air from Screening to Day 30	FAS, PPS
Table 14.2.2.2	Change in PTA Air from Screening to Day 90	FAS, PPS
Table 14.2.2.3	Pure Tone Audiogram (PTA Bone) Values over Time	FAS, PPS
Table 14.2.2.4	Change in PTA Bone from Screening to Day 8	FAS, PPS
Table 14.2.2.5	Change in PTA Bone from Screening to Day 30	FAS, PPS
Table 14.2.2.6	Change in PTA Bone from Screening to Day 90	FAS, PPS
Table 14.2.3.1	Word Recognition Scores over Time	FAS, PPS
Table 14.2.3.2	Change in Word Recognition Score from Screening to Day 8	FAS, PPS
Table 14.2.3.3	Change in Word Recognition Score from Screening to Day 30	FAS, PPS
Table 14.2.3.4	Change in Word Recognition Score from Screening to Day 90	FAS, PPS
Table 14.2.4.1	Fibrinogen Values over Time	FAS
Table 14.2.4.2	Change in Fibrinogen from Screening to Day 2	FAS
Table 14.2.4.3	Change in Fibrinogen from Screening to Day 8	FAS
Table 14.2.5.1	Biomarker Values over Time	FAS
Table 14.2.5.2	Change in Biomarkers from Screening to Day 8	FAS
Table 14.2.5.3	Correlation of Biomarkers with Clinical Effects	FAS
Table 14.2.6.1	Patient Assessment on Hearing Impairment over Time	FAS
Table 14.2.6.2	Change in Patient Assessment from Screening to Day 8	FAS
Table 14.2.6.3	Change in Patient Assessment from Screening to Day 30	FAS
Table 14.2.6.4	Change in Patient Assessment from Screening to Day 90	FAS
Table 14.2.6.5	Categorical Analysis of Patient Assessment on Hearing Impairment	FAS
Table 14.2.7.1	Physician Assessment on Hearing Impairment over Time	FAS
Table 14.2.7.2	Change in Physician Assessment from Screening to Day 8	FAS
Table 14.2.7.3	Change in Physician Assessment from Screening to Day 30	FAS

Number	Title	Analysis Population
Table 14.2.7.4	Change in Physician Assessment from Screening to Day 90	FAS
Table 14.2.7.5	Categorical Analysis of Physician Assessment on Hearing Impairment	FAS
Table 14.2.8.1	Tinnitus Severity over Time	FAS
Table 14.2.8.2	Change in Tinnitus Severity from Screening to Day 8	FAS
Table 14.2.8.3	Change in Tinnitus Severity from Screening to Day 30	FAS
Table 14.2.8.4	Change in Tinnitus Severity from Screening to Day 90	FAS
Table 14.3.1.1	Overview on Adverse Events	SFS
Table 14.3.1.2	Treatment-Emergent Adverse Events	SFS
Table 14.3.1.3	Treatment-Emergent Adverse Events by Maximum Intensity	SFS
Table 14.3.1.4	Treatment-Emergent Adverse Events Related to IMP	SFS
Table 14.3.1.5	Treatment-Emergent Adverse Events Leading to Discontinuation	SFS
Table 14.3.1.6	Treatment-Emergent Adverse Events by Action Taken	SFS
Table 14.3.1.7	Treatment-Emergent Adverse Events by Outcome	SFS
Table 14.3.1.8	Glossary of all Adverse Events	SFS
Table 14.3.2.1	Serious Treatment-Emergent Adverse Events	SFS
Table 14.3.2.2	Serious Treatment-Emergent Adverse Events Related to IMP	SFS
Table 14.3.2.3	Serious Treatment-Emergent Adverse Events Leading to Discontinuation	SFS
Table 14.3.4.1	Numeric Laboratory Values at Baseline and Changes	SFS
Table 14.3.4.2	Shift Analysis of Numeric Laboratory Values	SFS
Table 14.3.4.3	Analysis of Categorical Laboratory Values	SFS
Table 14.3.4.4	Clinically Relevant Laboratory Values	SFS
Table 14.3.5	General Physical Examination	SFS
Table 14.3.6	Otolaryngological Examination	SFS
Table 14.3.7.1	Vital Signs – Values per Visit and Absolute Change	SFS
Table 14.3.7.2	Pulse Pattern by Visit	SFS
Table 14.3.7.3	Shift Tables for Vital Signs	SFS

13.3. Table of Patient Data Listings

[To be included in Appendix 16.2.x of the CSR]
The numbering of data listings follows the ICH E3 guideline.

Number	Title	Analysis Population
Listing 16.2.1.1	Patient Disposition	Randomized set
Listing 16.2.1.2	Study Termination	Randomized set
Listing 16.2.2	Protocol Deviations	Randomized set
Listing 16.2.3.1	Patients Excluded from Analysis Sets	Randomized set
Listing 16.2.3.2	Description of Inclusion and Exclusion Criteria	-
Listing 16.2.3.3	Fulfilment of Inclusion and Exclusion Criteria	Randomized set
Listing 16.2.4.1	Demographic Data and Birth Control	Randomized set
Listing 16.2.4.2	Prior and Concomitant Medication	Randomized set
Listing 16.2.5.1	Visit Dates	Randomized set
Listing 16.2.5.2	Unscheduled Visits	Randomized set
Listing 16.2.5.3	Study Drug Administration	Randomized set
Listing 16.2.6.1	Pure Tone Audiograms (Air and Bone)	Randomized set
Listing 16.2.6.2	Speech Intelligibility Test	Randomized set
Listing 16.2.6.3	Otolaryngological Examination	Randomized set
Listing 16.2.6.4	Tinnitus and Vertigo Assessment	Randomized set
Listing 16.2.7.1	Adverse Events	Randomized set
Listing 16.2.7.2	Serious Adverse Events	Randomized set
Listing 16.2.8.1	Laboratory Data: Hematology	Randomized Set
Listing 16.2.8.2	Laboratory Data: Blood Chemistry	Randomized Set
Listing 16.2.8.3	Laboratory Data: Coagulation	Randomized Set
Listing 16.2.8.4	Laboratory Data: Urinalysis	Randomized Set
Listing 16.2.8.5	Laboratory Data: Antibodies	Randomized Set
Listing 16.2.8.6	Laboratory Data: Biomarkers	Randomized Set
Listing 16.2.9.1	General Physical Examination	Randomized set
Listing 16.2.9.2	Vital Signs	Randomized set

13.4. Layout of End-of-Text Tables and Listings

Issue	Specification
Basic layout of 'End-of-text' tables and listings (header, footnote)	3 horizontal lines per page: one above column headers, one below column headers, one below the body of the table or listing (footnotes below this last line).<br>Footnotes for all tables and listings:<br>`Program xxx.SAS, executed on DDMONYYYY at HH:MM`<br>`PAGE 1 OF X`
Page margins<br>based on DIN-A4 sized page	Landscape format:<br>Top 3 cm, bottom 2 cm, left 2.5 cm, right 2 cm
Font and font size	Courier New 9
Column header	First letter of column capitalized (if applicable)
Subject or Patient as label?	Patient
Subject identification numbers	Use Patient number as shown in the CRF
Order in subject data listings	Randomized patients by Treatment group, Patient number and Visit (if applicable)
Labels and order of treatment groups	First 'Ancrod', then 'Placebo', then 'Overall'
Labels and order of visits	Visit 1 (Screening)<br>Visit 2 (Day 2)<br>Visit 3 (Day 4)<br>Visit 4 (Day 6)<br>Visit 5 (Day 8)<br>Visit 6 (Day 30)<br>Visit 7 (Day 90)
Labels for descriptive summary statistics for continuous variables	Use n, Mean, SD, Min, Median, Max<br>(instead of number of non-missing values, arithmetic mean, standard deviation, minimum value, median, and maximum value)
Display of categorical variables in frequency tables	Display those categories present in the data, including missing values; omit categories that do not occur
In case a category does not occur within one treatment group	Display absolute frequency 0 (instead of "-")<br>"-" must only be used if the category or table cell is impossible
Display of absolute frequencies and percentages in tables	Frequencies are presented right aligned, followed by left aligned percentages in brackets, without % sign.<br>Percentages are presented with one decimal place, 0 and 100 percentages without any decimal place:<br>xx (xx.x)<br>x (x.x)<br>0 (0)<br>xxx (100)<br>Percentages greater than 0 but rounded to 0.0 will be written as '<0.1'; percentages smaller than 100 but rounded to 100.0 will be written as '>99.9'.
P-values	P-values will be quoted with four decimal places;<br>p-values which round to 0 will be reported as '<0.0001',<br>p-values rounding to 1 will be displayed as '>0.9999'.
Display of ranges	xx – yy
Display of 2-sided confidence intervals	[lower limit ; upper limit]
Display of units	Presentation case-sensitive and in round brackets, e.g. '(kg)'

13.5. Documentation of Statistical Methods

[To be included in Appendix 16.1.9 of the CSR]

For the primary efficacy variable, confirmative testing is performed with the aim to show superiority in favor of the treatment with ancrod over placebo, applying a two-sided 5 % significance level. All other variables are exploratively compared between treatment groups.

For quantitative efficacy variables, statistical testing to compare both treatment groups is done using an analysis of covariance (ANCOVA) based on the generalized linear model. Qualitative parameters are analyzed with Fisher's exact test (2×2-tables) or the Freeman-Halton test (tables larger than 2×2).

13.6. Table of 'In Text' TFLs

The generation of a clinical study report (CSR) or a statistical report is not contracted. Beside the end-of-text TFLs listed in Sections 13.2 and 13.3, separate specific in-text tables will be generated by YYY upon request only.

13.7. Shells for TFLs

For the general layout of end-of-text tables and listings, see Section 13.4. Shells for TFLs (so-called 'mock TFLs') will not be generated.

13.8. Raw Statistical Output

[To be included in Appendix 16.1.9 of the CSR]
Raw output of all applied SAS procedures will be stored in study-specific folders. It is planned to hand over SAS raw outputs referring to the primary efficacy analysis. For all other variables, only the agreed end-of-text tables that are based on the raw output will be compiled.

13.9. Data Deliverables

The following deliverables will be handed over to NORDMARK after study completion:

- A document containing all end-of-text tables and figures
- A document containing all patient data listings
- A document containing the raw output from SAS procedures.
