# Supplementary material for: Randomized, placebo-controlled study on efficacy, safety and tolerability of drug-induced defibrinogenation for sudden sensorineural hearing loss: the lessons learned
Source: Eur Arch Otorhinolaryngol. 2023 Mar 7;280(9):4009–18. doi: 10.1007/s00405-023-07896-z (PMC10382375; doi:10.1007/s00405-023-07896-z)
Supplement: Supplementary file 2 — Supplementary file2 Adverse events with absolute and relative frequencies by system organ class and preferred term (PDF 120 KB) [file 405_2023_7896_MOESM2_ESM.pdf]

Supplemental Material 2

eTable. Adverse Events with Absolute and Relative Frequencies by System Organ Class and Preferred Term

**Adverse Event**	**Ancrod (n=22)**		**Placebo (n=9)**		**Total (n=31)**	
**System Organ Class** Preferred Term[a]	**related, n (%)**	**not related, n (%)**	**related, n (%)**	**not related, n (%)**	**related, n (%)**	**not related, n (%)**
EAR AND LABYRINTH DISORDERS						
Deafness neurosensory	0	1 (4.5)	0	0	0	1 (3.2)
Deafness unilateral	0	1 (4.5)	0	0	0	1 (3.2)
Tinnitus	1 (4.5)	0	0	0	1 (3.2)	0
GASTROINTESTINAL DISORDERS						
Abdominal pain upper	0	0	1 (11.1)	0	1 (3.2)	0
Diarrhea	0	0	0	1 (11.1)	0	1 (3.2)
Dyspepsia	0	1 (4.5)	0	0	0	1 (3.2)
Nausea	1 (4.5)	1 (4.5)	0	0	1 (3.2)	1 (3.2)
GENERAL DISORDERS AND ADMINISTRATION SITE DISORDERS						
Chest pain	0	0	1 (11.1)	0	1 (3.2)	0
Chills	1 (4.5)	0	0	0	1 (3.2)	0
Fatigue	1 (4.5)	1 (4.5)	0	0	1 (3.2)	1 (3.2)
Feeling hot	1 (4.5)	0	1 (11.1)	0	2 (6.5)	0
INFECTIONS AND INFESTATIONS						
Acute sinusitis	0	1 (4.5)	0	0	0	1 (3.2)
Upper respiratory tract infection	0	1 (4.5)	0	0	0	1 (3.2)
Varicella zoster virus infection	0	1 (4.5)	0	0	0	1 (3.2)
INJURY, POISONING AND PROCEDURAL COMPLICATIONS						
Limb injury	0	1 (4.5)	0	1 (11.1)	0	2 (6.5)
INVESTIGATIONS						
Body temperature increased	1 (4.5)	0	0	0	1 (3.2)	0
MUSCULOSKELETAL AND CONNECTIVE TISSUE DISORDERS						
Back pain	0	2 (9.1)	0	0	0	2 (6.5)
NERVOUS SYSTEM DISORDERS						
Balance Disorder	0	0	0	1 (11.1)	0	1 (3.2)
Dizziness	0	1 (4.5)	0	0	0	1 (3.2)
Headache	0	3 (13.6)	1 (11.1)	0	1 (3.2)	3 (9.7)
Hypoesthesia	0	1 (4.5)	0	0	0	1 (3.2)
Muscle contractions involuntary	0	0	1 (11.1)	0	1 (3.2)	0
Syncope	0	1 (4.5)	0	0	0	1 (3.2)
REPRODUCTIVE SYSTEM AND BREAST DISORDERS						
Vaginal hemorrhage	1 (4.5)	0	0	0	1 (3.2)	0
RESPIRATORY, THORACIC AND BREAST DISORDERS						
Asthma	0	1 (4.5)	0	0	0	1 (3.2)
Oropharyngeal pain	0	1 (4.5)	0	0	0	1 (3.2)
SKIN AND SUBCUTANEOUS TISSUE DISORDERS						
Erythema	1 (4.5)	0	0	0	1 (3.2)	0
Hyperhidrosis	0	0	1 (11.1)	0	1 (3.2)	0
VASCULAR DISORDERS						
Flushing	0	1 (4.5)	0	0	0	1 (3.2)
Hematoma	1 (4.5)	0	0	1 (11.1)	1 (3.2)	1 (3.2)
Hypertension	0	1 (4.5)	0	0	0	1 (3.2)
Hypotension	1 (4.5)	0	0	0	1 (3.2)	0

[a] Medical Dictionary for Regulatory Activities (MedDRA) V 20.1
